# The Bacterial Outer Membrane Vesicle-Cloaked Immunostimulatory Nanoplatform Reinvigorates T Cell Function and Reprograms Tumor Immunity

**DOI:** 10.1021/acsnano.5c02541

**Published:** 2025-05-20

**Authors:** Yu-Han Lin, Chia-Wei Chen, Mei-Yi Chen, Li Xu, Xuejiao Tian, Siu-Hung Cheung, Yen-Ling Wu, Natnaree Siriwon, Si-Han Wu, Kurt Yun Mou

**Affiliations:** † Taiwan International Graduate Program in Molecular Medicine, National Yang Ming Chiao Tung University and Academia Sinica, Taipei 11529, Taiwan; ‡ Graduate Institute of Nanomedicine and Medical Engineering, 38032 Taipei Medical University, Taipei 11031, Taiwan; § Institute of Biomedical Sciences, Academia Sinica, Taipei 11529, Taiwan; ∥ Research Center for Applied Sciences, Academia Sinica, Taipei 11529, Taiwan; ⊥ Chakri Naruebodindra Medical Institute, Faculty of Medicine Ramathibodi Hospital, 615959 Mahidol University, Samutprakarn 10540, Thailand; # International Ph.D. Program in Biomedical Engineering, Taipei Medical University, Taipei 11031, Taiwan; ∞ Nano Science and Technology Program, Taiwan International Graduate Program, Academia Sinica, Taipei, 11529, Taiwan; 8 Department of Engineering and System Science, National Tsing Hua University, Hsinchu, 30013, Taiwan

**Keywords:** tumor microenvironment, bacterial outer membrane vesicles, mesoporous silica nanoparticles, mitochondrial activity, T cell exhaustion phenotypes

## Abstract

Bacterial outer membrane vesicles (OMVs) represent powerful immunoadjuvant nanocarriers with the capacity to reprogram the tumor microenvironment (TME) and activate immune responses. Here, we investigate a nanotherapeutic platform integrating immunostimulatory cytosine–phosphate–guanine oligodeoxynucleotides (CpG-ODNs, hereafter termed CpG) into mesoporous silica nanoparticles cloaked with OMVs (CpG@MSN-PEG/PEI@OMVs) for cancer immunotherapy. Systemic administration of these nanohybrids facilitates precise tumor targeting, induces antitumor cytokines such as IFNγ, and suppresses immunosuppressive cytokine TGF-β, reshaping the TME. Additionally, CpG@MSN-PEG/PEI@OMVs promote M1 macrophage polarization, dendritic cell maturation, and the generation of durable tumor-specific immune memory, resulting in pronounced tumor regression with minimal systemic toxicity. The platform demonstrates efficacy against metastatic and solid tumor models including 4T1 breast and MC38 colorectal cancers. Transcriptomic analyses reveal that CpG@MSN-PEG/PEI@OMVs enhance mitochondrial oxidative phosphorylation in T cells within tumor-draining lymph nodes, mitigating T cell exhaustion and restoring metabolic fitness. These results support the potential of CpG@MSN-PEG/PEI@OMVs as a modular nanoplatform to modulate innate and adaptive immunity in cancer immunotherapy.

## Graphical abstract

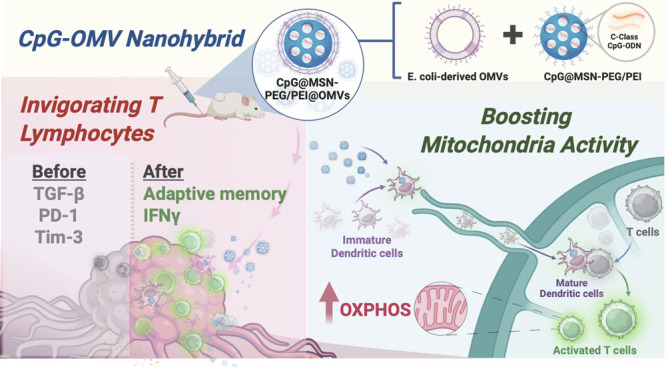

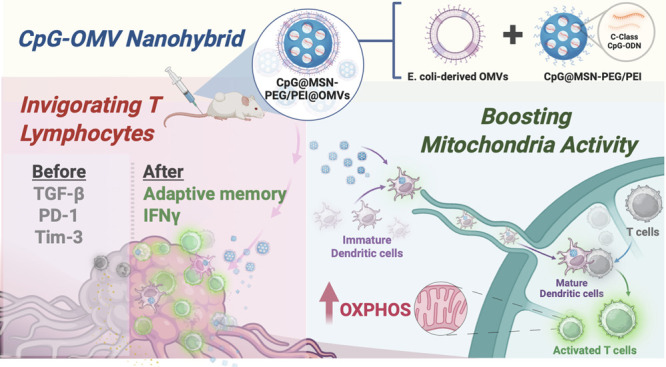

## 1 Introduction

Harnessing bacterial outer membrane vesicles (OMVs) serves as an effective strategy in cancer immunotherapy due to their ability to modulate immune responses within the tumor microenvironment (TME). OMVs, enriched with pathogen-associated molecular patterns (PAMPs), can effectively trigger innate immunity and act as robust immunoadjuvants.^1^ Although OMVs demonstrate considerable potential in preclinical settings, current cancer immunotherapy primarily focuses on well-established strategies, such as immune checkpoint blockade (ICB) therapies targeting PD-1/PD-L1, and neoantigen-based treatments, which have shown remarkable efficacy over the past decade.^2,3^ However, approximately 20% of patients fail to benefit.^4–6^ This limitation is attributed to factors such as aberrant extracellular matrix deposition in tumors and systemic immunosuppressive cytokines, particularly transforming growth factor-β (TGF-β). TGF-β plays a significant role in creating an immunosuppressive TME by impairing the antigen presentation and inducing metabolic dysfunction in T cells. Specifically, it downregulates mitochondrial respiratory chain components, limiting oxidative phosphorylation (OXPHOS) and causing T cells to rely on glycolysis for energy. This metabolic shift is less efficient and insufficient to sustain the proper T cell activation and function. These factors, along with chronic antigenic stimulation, collectively lead to dysfunctional tumor-infiltrating dendritic cells (DCs) and exhausted tumor-specific T lymphocytes. Such conditions promote tumor immune evasion by restricting antigen presentation and supporting the heterogeneous expression of negative immunological checkpoints on T lymphocytes within the TME.^7–10^

To address these challenges, a wide range of nanoparticle (NP) systems—including liposomes, polymeric NPs, and DC-targeted nanocarriers—have been developed to enhance antigen delivery, lymph node targeting, and immune activation. Notably, lipid NPs delivering neoantigen-encoding mRNA and polymer-based NPs encapsulating immune adjuvants have shown promise in eliciting antigen-specific T cell responses.^11,12^ However, these strategies often rely on tumor-specific antigens (TSAs) and may fall short in reprogramming the immunosuppressive TME, largely due to their limited capacity to diversify the antigenic repertoire within the tumor milieu—an issue that remains a significant barrier in cancer immunotherapy.

In contrast to TSA-dependent platforms, bacterial OMVs offer a TSA-independent mechanism for immune activation. Their inherent immunogenicity—characterized by a broad range of PAMPs and bacterial antigens—enriches immunostimulatory signals within the TME, enhancing local immunogenicity. OMVs are the following: they promote phagocytosis, facilitate efficient antigen presentation by DCs, and stimulate robust innate and adaptive immune responses. These features enable OMVs to modulate the immunosuppressive TME, address limitations of conventional NP systems, and support antitumor immunity.^13^ Moreover, this localized immune activation within the TME significantly promotes the infiltration and activation of immune cells, enabling targeted and sustained anticancer responses.^14–16^ Recent studies have explored OMVs as carriers to boost immune responses against tumors; for example, Liang et al. developed calcium phosphate (CaP) hybrid OMVs expressing SIRPα to enhance phagocytosis by antigen-presenting cells (APCs) while encapsulating GM-CSF to stimulate tumor-specific immune activation.^15^ These findings support further investigation of integrating the nanomedicine with the OMVs to address immune dysfunction within the TME.

In parallel, cell membrane (CM) wrapping technology facilitates the integration of synthetic NPs with various CMs.^17^ This approach preserves the natural functions of the cell’s outer membrane while retaining the physicochemical properties of the core NPs, resulting in CM-NPs with enhanced biocompatibility and functionality.^18,19^ Based on these principles, the use of OMVs may enable tumor targeting by their small size and natural composition. This targeting capability likely arises from passive accumulation via the enhanced permeability and retention (EPR) effect, which is facilitated by the leaky vasculature of tumors.^20,21^ However, challenges remain in utilizing OMV-based nanocarriers to modulate innate immunity while mitigating T cell exhaustion within the TME. These limitations stem in part from immune suppression driven by lipopolysaccharide (LPS) tolerance and complications related to systemic administration.^22–24^ Overcoming these barriers is essential to fully harnessing the therapeutic potential of the OMVs.

To address these challenges, we developed an OMV-integrated nanoplatform based on mesoporous silica nanoparticles (MSNs) functionalized with poly­(ethylene glycol) (PEG) and polyethylenimine (PEI), referred to as MSN@PEG/PEI. PEGylation serves to stabilize the composite structure and regulate interactions between PEI and the OMVs, ensuring controlled assembly and optimal biointerface properties. MSN@PEG/PEI also acts as a structural scaffold to support OMV coating, thereby enhancing immunostimulatory effects while minimizing systemic toxicity (Scheme 1).

**Scheme 1:**
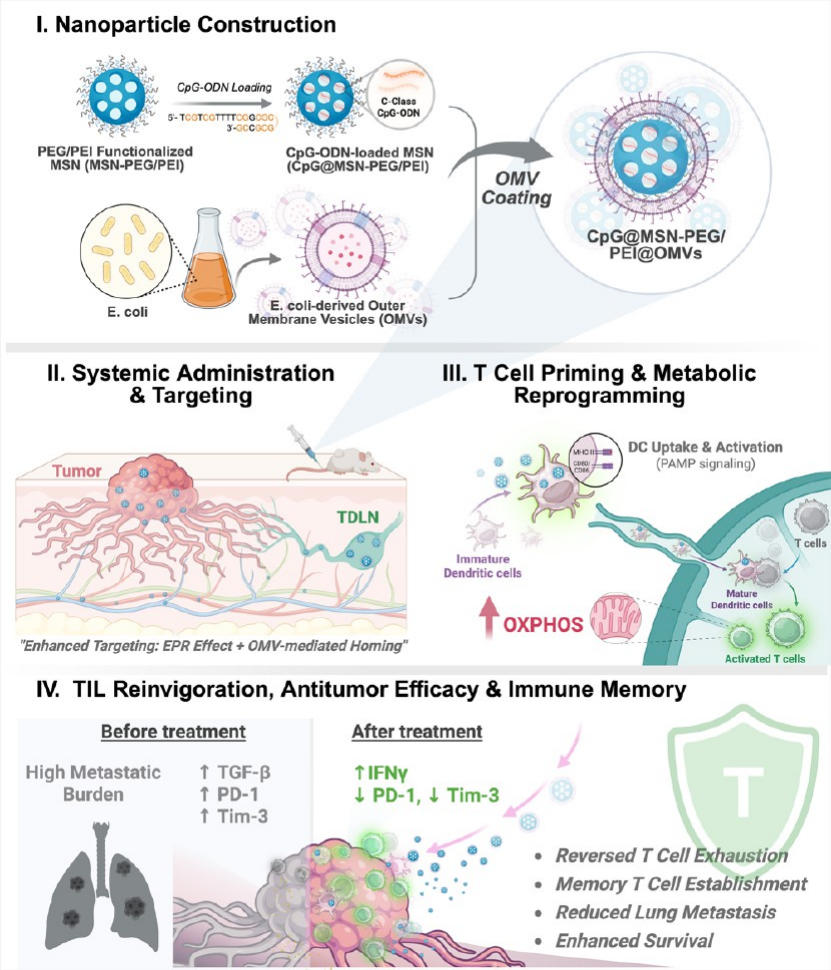
Schematic Overview of CpG@MSN-PEG/PEI@OMVs for Reinvigorating TILs and Enhancing Antitumor Immunity^a^

a (I) NP Construction: mesoporous silica nanoparticles (MSNs) are functionalized with PEG/PEI, loaded with C-class CpG-ODNs, and coated with Escherichia coli-derived OMVs. (II) Systemic Administration and Targeting: upon IV injection, the nanohybrids home to tumors and tumor-draining lymph nodes (TDLNs) via the EPR effect and OMV-mediated targeting. (III) T Cell Priming and Metabolic Reprogramming: APCs (e.g., DCs) internalize the nanohybrids, upregulate costimulatory molecules, and foster T cell activation with increased oxidative phosphorylation (OXPHOS). (IV) TIL Reinvigoration, Antitumor Efficacy, and Immune Memory: in the TME, tumor-infiltrating lymphocytes (TILs) are reinvigorated through downregulation of TGF-β, PD-1, and Tim-3, while IFNγ production is enhanced. These changes reverse T cell exhaustion, reduce metastasis, and support long-lasting immune memory.

First, unmethylated cytosine–phosphate–guanine oligodeoxynucleotides (CpG-ODN)—short, synthetic single-stranded DNA molecules—are incorporated as potent immune adjuvants to boost OMV-induced innate immunity and to shift APC-T cell interactions toward positive checkpoint signaling rather than tolerance.^25^ Second, encapsulating CpG-ODN within MSN@PEG/PEI NPs enhances biosafety, prevents premature degradation, prolongs tumor retention, and enables codelivery of immunostimulatory DNA and bacterial virulence factors to tumor-infiltrating APCs.^26^ Finally, membrane-coated nanomaterials help mitigate the adverse effects typically associated with OMV administration, supporting safe and efficient intravenous delivery.^23^ Collectively, these findings support the potential utility of the use of OMV-based nanotherapeutics in modulating immunosuppressive TMEs and alleviating T cell exhaustion, providing a feasible strategy to enhance cancer immunotherapy.

In this work, we evaluate the application of integrating bacterial OMVs with CpG-ODN-loaded mesoporous silica NPs (CpG@MSN-PEG/PEI@OMVs) for cancer immunotherapy. These OMV-based nanohybrids exhibit multifunctional immunomodulatory properties, such as synergistic accumulation in tumors and TDLNs, induction of M1 macrophage polarization, promotion of DC maturation, and activation of functional tumor antigen-specific T cells. Notably, CpG@MSN-PEG/PEI@OMVs suppress systemic TGF-β levels—a key mediator of immunosuppression—while enhancing intratumoral interferon γ (IFNγ) production, thereby reshaping the immunosuppressive TME.

In addition, these nanohybrids effectively reverse T cell exhaustion, as evidenced by reduced levels of PD-1 and Tim-3 expression in both tumors and TDLNs, thereby restoring effector T cell functionality. Bulk and single-cell transcriptomic analyses further revealed profound immunometabolic reprogramming characterized by elevated mitochondrial respiration and oxidative phosphorylation in immune cells. These changes were accompanied by the downregulation of immunosuppressive genes and pathways and the upregulation of proinflammatory cytokines, chemokines, and histone modifiers as well as the activation of distinct myeloid and lymphocyte lineages.

Collectively, our findings demonstrate that CpG@MSN-PEG/PEI@OMVs modulate innate immune cells, support T cell function, and alter the TME to promote adaptive immune responses. By addressing barriers in cancer therapy—such as immune evasion and tumor relapse—this strategy supports the integration of innate and adaptive immunity to promote systemic antitumor effects with minimal toxicity.

## 2 Results and Discussion

### 2.1 Design and Characterization of OMV-Coated MSN-PEG/PEI NPs (MSN-PEG/PEI@OMVs)

To exploit the multifaceted interactions of bacterial OMVs with diverse biological entities,^27^ our objective was to integrate their innate tumor-targeting capability into a modular nanocarrier platform. In our previous work, we synthesized MSNs as detailed in the Experimental Section. To enable fluorescence tracking, MSN-PEG was conjugated with rhodamine isothiocyanate (RITC) to generate RMSN-PEG, enhancing its utility for biomedical applications. To enhance the OMV coating efficiency—facilitated by electrostatic interactions between negatively charged OMVs and the immunoadjuvant CpG—a positive surface charge was applied by incorporating cationic PEI, leading to the formation of RMSN-PEG/PEI (Scheme 1I).

As shown in Figure 1A, transmission electron microscopy (TEM) revealed that the NPs were uniformly dispersed and exhibited a well-defined hexagonal mesoporous structure with an average diameter of 25 nm. OMVs displayed a consistent bilayer morphology and spherical shape, with vesicle sizes ranging from 25 to 30 nm. For optimized coating, the particle concentrations of RMSN-PEG/PEI and OMVs were adjusted based on NP tracking analysis (Figure S1-1A,B), and the two components were fused via mechanical extrusion at a 1:50 particle number ratio. After purification, TEM images confirmed effective membrane coverage of RMSN-PEG/PEI by the OMVs, showing preserved lipid bilayer structures (∼5 nm thick) and visible pore arrangements. Dynamic light scattering (DLS) analysis revealed an increase in hydrodynamic diameter from 44.8 ± 0.7 nm (RMSN-PEG/PEI) to 54.0 ± 0.1 nm after the OMV coating in phosphate-buffered saline (PBS). The surface zeta potential shifted from +7.49 ± 0.23 mV to −15.8 ± 1.08 mV, indicating successful charge inversion due to OMV wrapping (Figure 1B). Meanwhile, SDS-PAGE confirmed the retention of characteristic OMV membrane proteins on RMSN-PEG/PEI@OMVs, validating coating integrity (Figure 1C). Importantly, RMSN-PEG/PEI@OMVs exhibited negligible cytotoxicity toward RAW 264.7 macrophage-like cells, even at high concentrations up to 1000 μg/mL (Figure S1-1C). These results indicate the structural uniformity and preliminary biocompatibility of RMSN-PEG/PEI@OMVs, warranting further evaluation as a nanotherapeutic platform.

**Figure 1:**
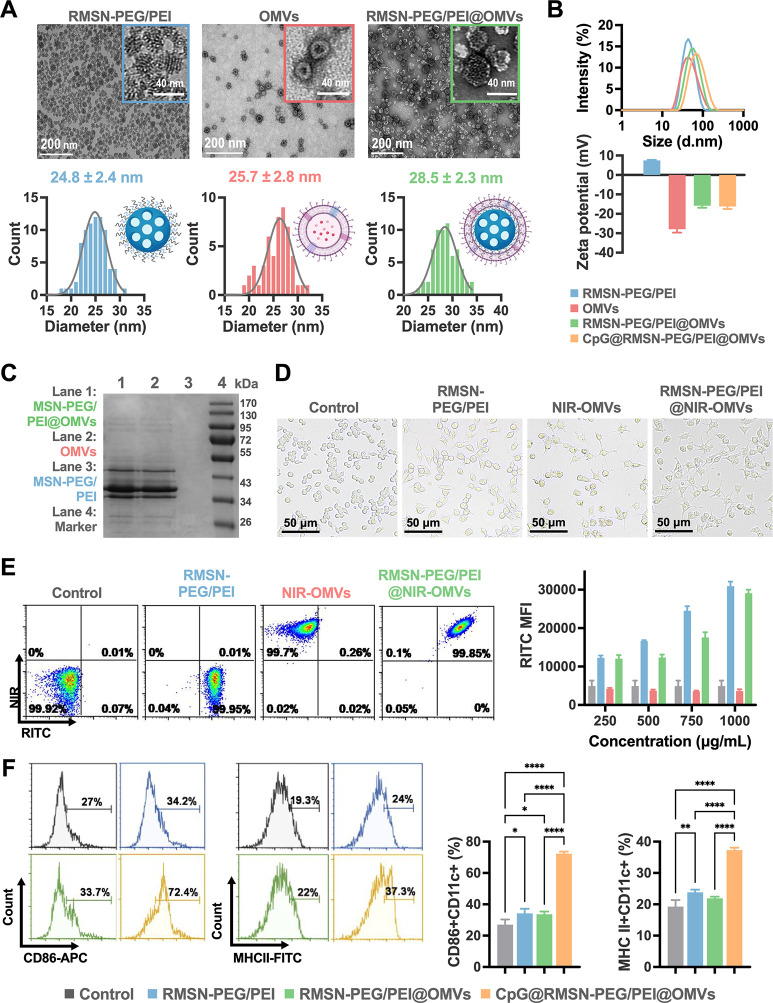
Characteristics and in vitro analysis of RMSN-PEG/PEI@OMVs NPs on the RAW 264.7 cell uptake and BMDC activation. (A) TEM images and particle size distribution histogram of RMSN-PEG/PEI, OMVs, and RMSN-PEG/PEI@OMVs. (B) Hydrodynamic diameters and ζ-potential of RMSN-PEG/PEI, OMVs, RMSN-PEG/PEI@OMVs, and CpG@RMSN-PEG/PEI@OMVs. (C) SDS-PAGE protein analysis showing the protein profiles of (1) MSN-PEG/PEI@OMVs, (2) OMVs, and (3) MSN-PEG/PEI. (D) Bright-field microscopic images depicting morphological changes in RAW 264.7 cells treated with PBS, RMSN-PEG/PEI, NIR-OMVs, and RMSN-PEG/PEI@NIR-OMVs. (E) Flow cytometry quantifying the uptake of RMSN-PEG/PEI, NIR-OMVs, and RMSN-PEG/PEI@NIR-OMVs in Raw 264.7 cells. (F) Expression levels of CD86 and MHC II on CD11c^+^ BMDCs at 24 h post-treatment with PBS (control), MSN-PEG/PEI, MSN-PEG/PEI@OMVs, and CpG@MSN-PEG/PEI@OMVs based on representative flow cytometry plots showing proportions of CD11c^+^CD86^+^ cells and CD11c^+^MHC II^+^ cells (*n* = 3 per group). Results are shown as the average ± standard deviation from selected experiments. Statistical significance was indicated by *p < 0.05, **p < 0.01, ***p < 0.001, and ****p < 0.0001, determined using one-way ANOVA with Tukey’s multiple comparisons test.

### 2.2 Interaction of RMSN-PEG/PEI@OMV with APCs In Vitro: Uptake, Morphology, and Immunostimulatory Effects

To investigate the interaction between MSN-PEG/PEI@OMVs and APCs, we conducted in vitro studies to examine cellular uptake and morphological changes. RMSN-PEG/PEI was synthesized, and the OMVs were stained with CellMask Deep Red (NIR-OMVs) to enable the assessment of uptake efficiency. RAW 264.7 cells were incubated with varying doses of RMSN-PEG/PEI, NIR-OMVs, or RMSN-PEG/PEI@NIR-OMVs for 24 h, and the cell morphology was documented via bright-field microscopy. Uptake was subsequently quantified by flow cytometric analysis of the RITC and NIR fluorescence signals. All formulations—RMSN-PEG/PEI, NIR-OMVs, and RMSN-PEG/PEI@NIR-OMVs—were efficiently internalized by RAW 264.7 cells (Figures 1E and S1-1D). Confocal imaging further confirmed the intracellular localization of RMSN-PEG/PEI@OMVs, with RITC signals clearly distributed around the DAPI-stained nucleus (Figure S1-1E). As shown in Figure 1D, cells treated with RMSN-PEG/PEI maintained a spherical morphology, resembling those of untreated controls. In contrast, treatment with either NIR-OMVs or RMSN-PEG/PEI@NIR-OMVs induced marked morphological changes, characterized by polygonal or dendritic-like shapes—features indicative of macrophage activation, likely triggered by the intrinsic adjuvanticity of OMVs.^28^ Given that Toll-like receptor 9 (TLR9) is an endosomal receptor requiring particle internalization for downstream signaling, the efficient uptake of CpG-loaded RMSN-PEG/PEI@OMVs (CpG@RMSN-PEG/PEI@OMVs) by APCs is essential for immunostimulatory efficacy. Accordingly, CpG@RMSN-PEG/PEI@OMVs were prepared, and DLS measurements indicated an average hydrodynamic diameter of 68.7 ± 0.2 nm, approximately 15 nm larger than RMSN-PEG/PEI@OMVs (Figure 1B). As the principal APCs responsible for initiating adaptive immunity,^29^ DCs were used to further evaluate the immunoactivation potential of these nanohybrids. Bone marrow-derived DCs (BMDCs) were treated with different formulations for 24 h, followed by flow cytometric analysis using antibodies against MHC class II and CD86. Both RMSN-PEG/PEI alone and RMSN-PEG/PEI@OMVs (250 μg/mL) significantly increased the proportion of CD11c^+^CD86^+^ BMDCs relative to untreated controls. Furthermore, the incorporation of CpG into RMSN-PEG/PEI@OMVs amplified activation markers, leading to a further increase in CD11c^+^MHC II^+^ and CD11c^+^CD86^+^ BMDC populations (Figure 1F). Taken together, these results demonstrate that CpG-loaded RMSN-PEG/PEI@OMVs enhance cellular uptake, induce APC morphological changes, and upregulate BMDC activation markers, supporting their immunostimulatory effects in vitro.

### 2.3 Biosafety Analysis of RMSN-PEG/PEI@OMVs

Prior to in vivo evaluation, the biosafety and biocompatibility of RMSN-PEG/PEI@OMVs were assessed in tumor-bearing and healthy mice. Blood samples were collected via cardiac puncture for serum biochemistry and complete blood count (CBC) analyses. Serum biochemistry focused on liver function markers (alkaline phosphatase [ALKP] and alanine aminotransferase [ALT]), renal function (blood urea nitrogen), indicators of hepatic/immune status (total protein [TP] and albumin), and lactate dehydrogenase [LDH] as a broad marker of tissue injury (including heart, liver, muscle, kidney, and lung). In 4T1 tumor-bearing mice, ALKP levels were lower than in healthy controls but remained within the normal range, suggesting only a moderate hepatic impact from tumor burden. Transient elevations in serum LDH and ALT were observed 24 h after administration of RMSN-PEG/PEI@OMVs or CpG@RMSN-PEG/PEI@OMVs, but values remained within normal ranges (Figure S1-2).

CBC analysis focused on red blood cells, neutrophils, lymphocytes, monocytes, and platelets. By day 30, neutrophil, lymphocyte, and monocyte counts were elevated in tumor-bearing mice relative to healthy controls, reflecting a tumor-induced systemic inflammatory response. Interestingly, mice treated with two doses of RMSN-PEG/PEI@OMVs or CpG@RMSN-PEG/PEI@OMVs exhibited reduced neutrophil and lymphocyte counts, potentially reflecting the treatments’ antitumor efficacy (Table S1).

To minimize the risk of systemic inflammatory toxicity or cytokine release syndrome (CRS), a low-volume, spaced dosing regimen was employed: each mouse received two intravenous injections at a four-day interval. Collectively, these observations support the in vivo biosafety of RMSN-PEG/PEI-based nanotherapeutics, enabling their evaluation in tumor models.

### 2.4 Biodistribution and Targeting Efficacy of MSN-PEG/PEI@OMVs in Tumors and TDLNs

After validating the encapsulation of the OMV membrane on RMSN-PEG/PEI and confirming its biosafety, we evaluated the in vivo tumor-targeting capability of RMSN-PEG/PEI@OMVs. Biodistribution was assessed by tracking RITC and NIR signals using an in vivo imaging system (IVIS) following systemic administration in 4T1 tumor-bearing mice. Mice received intravenous injections of RMSN-PEG/PEI, NIR-OMVs, or RMSN-PEG/PEI@NIR-OMVs. 24 h later, whole-body fluorescence imaging and organ-specific analysis were performed, including the heart, liver, spleen, lungs, kidneys, axillary lymph nodes, inguinal lymph nodes, TDLNs, and tumors. Mice treated with RMSN-PEG/PEI@NIR-OMVs exhibited significantly elevated RITC and NIR fluorescence at tumor sites compared to those administered PBS (control), RMSN-PEG/PEI, or NIR-OMVs alone (Figure 2A). Ex vivo imaging of excised tumors further confirmed enhanced dual-fluorescence signals in the RMSN-PEG/PEI@NIR-OMVs group (Figure 2B,C). Notably, the tumor-to-liver fluorescence ratio in this group was 26.8-fold higher than that of RMSN-PEG/PEI and 2.6-fold higher than that of NIR-OMVs, underscoring the superior tumor-targeting efficiency of the RMSN-PEG/PEI@NIR-OMV formulation.

**Figure 2:**
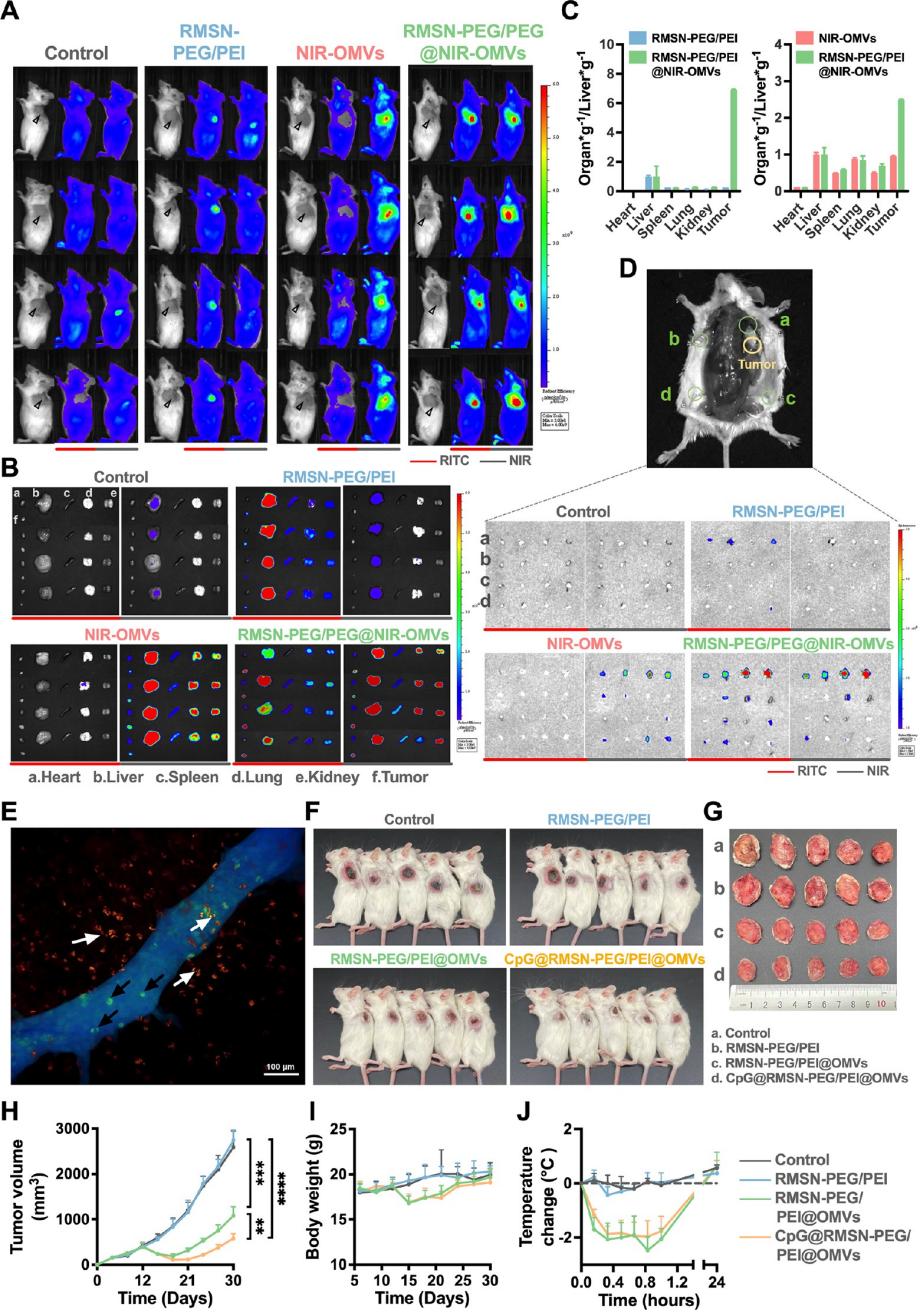
Targeted systemic delivery of RMSN-PEG/PEI@OMVs to tumors and TDLNs for antitumor efficacy. (A) IVIS imaging of 4T1 tumor-bearing mice 24 h postinjection of PBS, RMSN@PEG/PEI, NIR-OMVs, or RMSN-PEG/PEI@NIR-OMVs. (B) Ex vivo fluorescence images of the tumor tissues and major organs from sacrificed mice (*n* = 4 per group). (C) Semiquantitative evaluation of the tumor fluorescent signals. (D) Ex vivo fluorescence images of TDLNs from sacrificed mice (*n* = 4 per group). (E) Visualization of CpG@RMSN-PEG/PEI@OMVs inside and outside the blood vessel. Blue denotes the vessel structure. The green signals (indicated by black arrows) represent MSNs located inside the vessel, whereas the gold signals (indicated by white arrows) represent MSNs outside the vessel. (F) In vivo imaging of mice 7 days after the final injection of PBS, RMSN@PEG/PEI, RMSN-PEG/PEI@OMVs, or CpG@RMSN-PEG/PEI@OMVs. (G) Photographs of tumor collected on day 30. (H) Tumor volume and (I) body weight of 4T1 tumor-bearing mice (*n* = 6 per group) measured after two administrations of the indicated treatments. (J) Rectal temperature changes in mice with 4T1 tumors (*n* = 3 per group) post a single injection of the indicated treatments. Statistical significance was indicated by *p < 0.05, **p < 0.01, ***p < 0.001, and ****p < 0.0001, determined using two-way repeated-measures ANOVA with Tukey’s multiple comparisons test.

Beyond tumor targeting, TDLNs are essential for priming T lymphocytes against tumor-associated antigens.^30^ We thus examined the specific accumulation of RMSN-PEG/PEI@NIR-OMVs in the TDLNs. As shown in Figure 2D, ex vivo imaging revealed significantly elevated NIR signals in TDLNs of mice treated with NIR-OMVs or RMSN-PEG/PEI@NIR-OMVs. Furthermore, colocalization of RITC and NIR fluorescence within the same TDLNs confirmed successful dual-particle homing in the RMSN-PEG/PEI@NIR-OMV group. The near-complete overlap of red (RMSN-PEG/PEI) and deep-red (OMV) signals validated the effective delivery of the nanohybrid to lymphatic sites. These findings demonstrate the combined targeting effects of RMSN-PEG/PEI and OMVs, resulting in efficient and selective deposition in both tumors and TDLNs. Light sheet microscopy (Figure 2E) further revealed that CpG@RMSN-PEG/PEI@OMVs localized both within and beyond tumor vasculature, confirming their capacity to penetrate the TME. In line with these biodistribution patterns, we observed visible darkening of tumor tissue surfaces 24 h postinjection with OMVs or RMSN-PEG/PEI@OMVs, suggestive of necrotic and apoptotic activity. In contrast, tumors from RMSN-PEG/PEI-treated mice displayed no gross or histological alterations (Figure S1-3A–C). This necrosis is likely attributable to immunogenic cell death triggered by OMVs, potentially due to their rich content of PAMPs, including LPS.^20^

### 2.5 Boosting Antitumor Therapeutic Efficacy with CpG@RMSN-PEG/PEI@OMVs

Previous studies have demonstrated that CpG oligonucleotides enhance both innate and adaptive immune responses via TLR9-dependent pathways, contributing to tumor regression.^31^ To leverage these effects, we selected C-class CpG, which combines the immunostimulatory properties of both A- and B-class CpG, thereby enabling balanced activation of immune pathways. This class is particularly well-suited for translational applications due to its potent efficacy and favorable safety profile.^32^ Building on these insights and the efficient tumor-targeting capacity of RMSN-PEG/PEI@OMVs, we evaluated the antitumor efficacy of CpG@RMSN-PEG/PEI@OMVs in vivo. On day 0, BALB/c mice were subcutaneously inoculated with luciferase-expressing 4T1 mammary carcinoma cells. Treatments—including PBS (control), RMSN-PEG/PEI, RMSN-PEG/PEI@OMVs, or CpG@RMSN-PEG/PEI@OMVs—were administered intravenously on days 13 and 16. Tumor growth and body weight were monitored throughout the experiment. As shown in Figure 2F–H, RMSN-PEG/PEI alone did not significantly suppress tumor progression, while both RMSN-PEG/PEI@OMVs and CpG@RMSN-PEG/PEI@OMVs led to marked reductions in the tumor volume. Tumor regrowth was observed on day 19 in the RMSN-PEG/PEI@OMVs group but was delayed until day 23 in the CpG@RMSN-PEG/PEI@OMVs group, indicating a prolonged antitumor effect with CpG incorporation. On day 30, the average tumor weight in the CpG@RMSN-PEG/PEI@OMVs group was approximately 3.2-fold lower than that of the control group and 1.5-fold lower than that of the RMSN-PEG/PEI@OMVs group (Figure S1-3D), confirming that CpG inclusion significantly enhanced therapeutic efficacy. Transient weight loss was observed in mice following administration of RMSN-PEG/PEI@OMVs or CpG@RMSN-PEG/PEI@OMVs, but body weights returned to baseline within 1 week after the final injection (Figure 2I). A similar pattern was observed in body temperature, where 4T1 tumor-bearing mice exhibited a temporary drop in temperature within 1 h of OMV-based treatments, but values normalized within 24 h across all groups (Figures 2J and S1-3E). Histological analyses of major organs—including the heart, liver, spleen, lungs, and kidneys—revealed no pathological abnormalities (Figure S1-4), further supporting the biocompatibility of CpG@RMSN-PEG/PEI@OMVs. Nevertheless, comprehensive toxicological assessments of OMV-based therapeutics of OMV will be essential for future clinical translation.

### 2.6 Enhancing Therapeutic Efficacy against Metastasis Using CpG@RMSN-PEG/PEI@OMVs

Metastasis to distant organs often precedes clinical cancer diagnosis.^33^ The lungs, owing to their extensive vasculature and physiological architecture, are among the most common metastatic sites across various cancer types.^34^ Compared with primary tumors, metastases pose greater treatment challenges, with limited effective therapeutic options currently available. To investigate whether CpG@RMSN-PEG/PEI@OMVs could suppress metastatic progression, we employed a luciferase-expressing 4T1 breast cancer model in female BALB/c mice. Mice were intravenously injected with PBS (control), RMSN-PEG/PEI, RMSN-PEG/PEI@OMVs, or CpG@RMSN-PEG/PEI@OMVs on days 13 and 16. Tumor progression and metastasis were tracked using IVIS bioluminescence imaging on days 6, 17, 27, and 30 (Figure 3A). As shown in Figure 3B, treatment with RMSN-PEG/PEI@OMVs or CpG@RMSN-PEG/PEI@OMVs led to a substantial reduction in systemic bioluminescence signals by day 30, in contrast to that of the control or RMSN-PEG/PEI groups. Given that the lungs are the primary site of metastasis in the 4T1 model, we further quantified metastatic burden at the experimental end point. Lung metastases were visualized using Indian ink perfusion and confirmed via histological analysis, including H&E and Ki67 staining (a marker of proliferation). As illustrated in Figure 3C,D, mice treated with PBS or RMSN-PEG/PEI exhibited extensive lung colonization, while those treated with RMSN-PEG/PEI@OMVs or CpG@RMSN-PEG/PEI@OMVs showed markedly fewer and smaller metastatic lesions. Notably, lung metastasis was nearly eliminated in the CpG@RMSN-PEG/PEI@OMV group.

**Figure 3:**
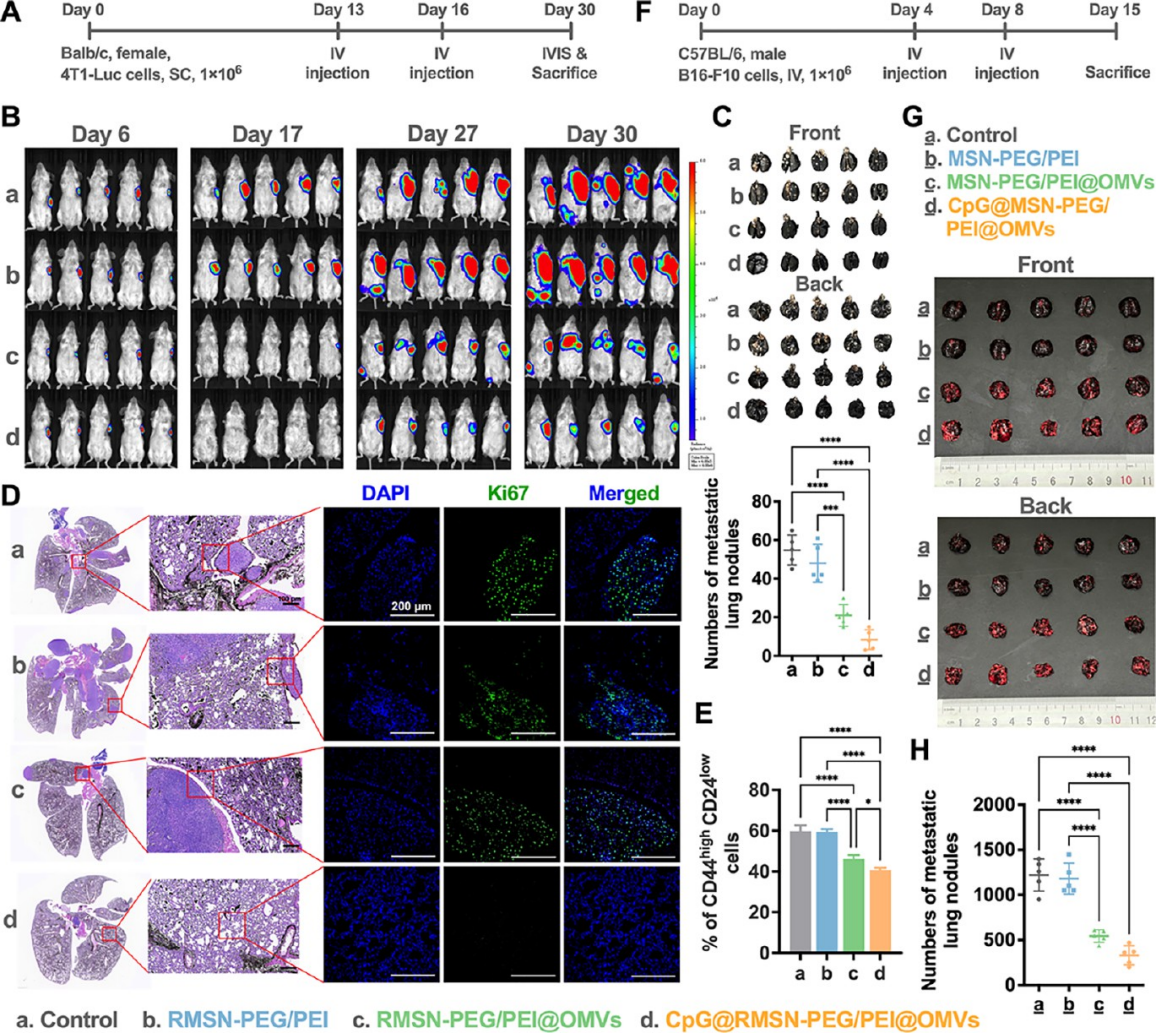
Therapeutic efficacy of CpG@MSN-PEG/PEI@OMVs in controlling metastasis in 4T1 and B16F10 melanoma mouse models. (A) Experimental scheme of mice implanted with Luc-4T1 cells followed by intravenous treatments with PBS, RMSN@PEG/PEI, RMSN-PEG/PEI@OMVs, or CpG@RMSN-PEG/PEI@OMVs. (B) IVIS imaging for tumor distribution analysis in the Luc-4T1 subcutaneous model (*n* = 5 per group). (C) Representative images of white lung metastatic nodules with positive India ink staining and semiquantitative analysis of 4T1 lung metastatic counts per mouse for each group (*n* = 4 per group). (D) Representative photographs of metastatic lung tissue in H&E-stained sections (scale bar: 100 μm) and fluorescent staining of lung sections with *K*_i_-67 in green for metastatic nodules and DAPI in blue for nuclei (scale bar: 200 μm). (E) Flow cytometry analysis of CD44^+^CD24^–^ cell proportions in 4T1 tumors. (F) Experimental scheme for pulmonary metastatic B16-F10 mouse melanoma models. (G) Representative photographs of lung metastatic foci collected 15 days postintravenous B16F10 cell administrations. (H) Semiquantitative analysis of B16F10 lung metastatic counts per mouse for each group (*n* = 5 per group). Statistical significance was indicated by *p < 0.05, **p < 0.01, ***p < 0.001, and ****p < 0.0001 determined using one-way ANOVA with Tukey’s multiple comparisons test.

At the cellular level, CD44^+^CD24^–^ breast cancer cells—classified as cancer stem cells—are known for their migratory potential and role in tumor recurrence and therapeutic resistance.^35^ Flow cytometry revealed that the proportion of CD44^+^CD24^–^ cells in tumors was significantly reduced following two doses of RMSN-PEG/PEI@OMVs or CpG@RMSN-PEG/PEI@OMVs (day 23), compared to that of the control and RMSN-PEG/PEI groups. Strikingly, the CpG@RMSN-PEG/PEI@OMV-treated mice exhibited the lowest levels of this aggressive stem cell population (Figures 3E and S2-1A), likely due to CpG-driven immune activation within the OMV nanohybrid, which disrupts cancer stem cell maintenance and metastasis.^36^

To validate these findings in a second model, we established a B16F10 melanoma lung metastasis model (Figure 3F). Consistent with 4T1 results, both RMSN-PEG/PEI@OMV and CpG@RMSN-PEG/PEI@OMV treatments significantly suppressed lung metastases and reduced metastatic nodule counts relative to controls, as confirmed by IVIS, lung imaging, and H&E staining (Figures 3G,H and S1-3F). Notably, lung metastasis was reduced by 1.7-fold in the CpG-loaded nanohybrid group compared to RMSN-PEG/PEI@OMVs alone, demonstrating the antimetastatic efficacy of CpG incorporation (Figure 3H).

### 2.7 Modulating DC and Macrophage Functions for Tumor Immunity Using CpG@RMSN-PEG/PEI@OMVs

To elucidate the immune mechanisms underlying tumor suppression, we first assessed APC uptake and maturation 24 h after a single intravenous administration of different treatments (Figure 4A). Mice treated with RMSN-PEG/PEI@OMVs or CpG@RMSN-PEG/PEI@OMVs exhibited significantly greater particle uptake by macrophages and DCs in lymph nodes and tumors, relative to other groups (Figures 4B,C and S2-1B,C).

**Figure 4:**
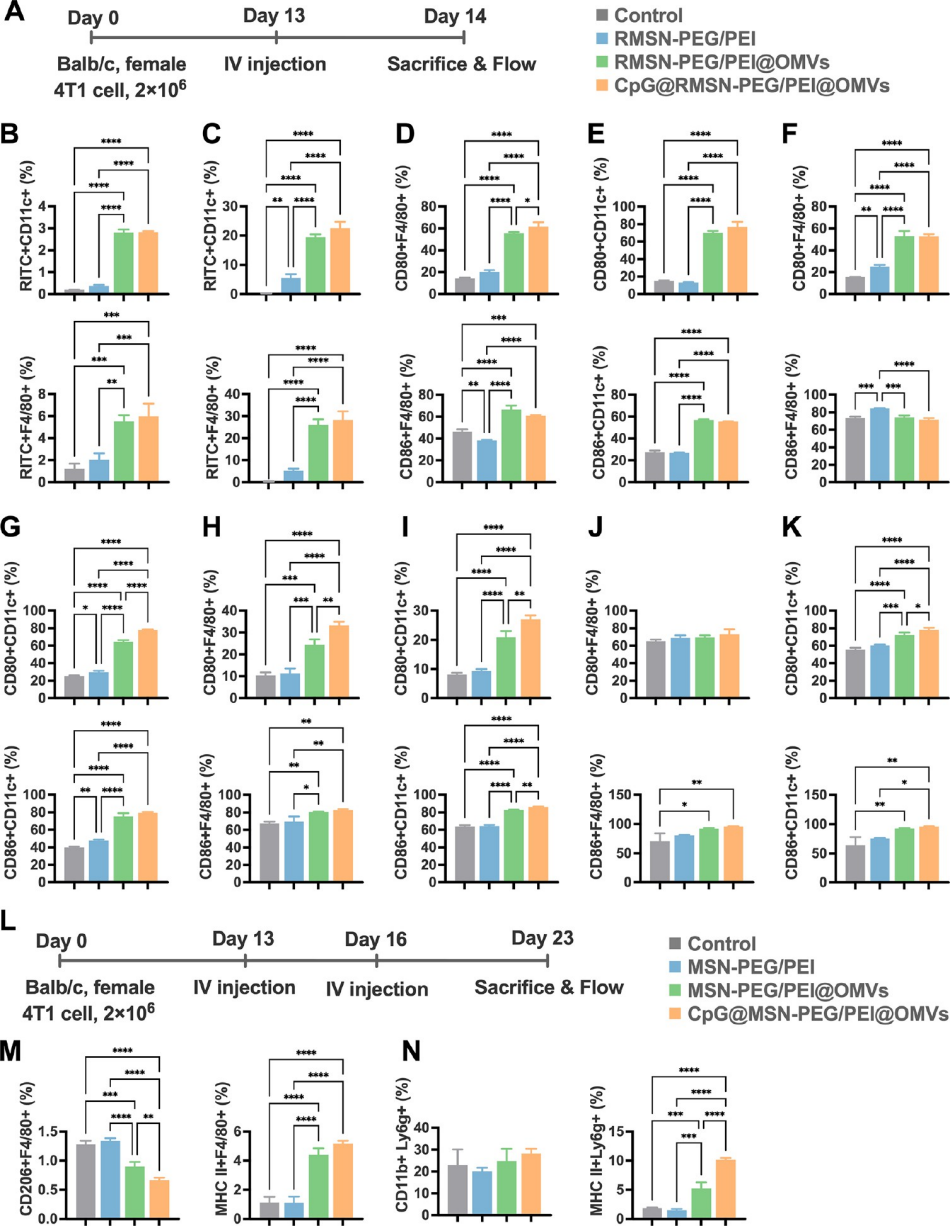
Augmented APC immunity by CpG@MSN-PEG/PEI@OMVs in vivo. (A) Schematic illustration of APC activation experiment postintravenous injection in 4T1-tumor-bearing mice with various treatments. (B) Flow cytometric evaluation of NP internalization by CD11c^+^ and F4/80^+^ cells in TDLNs and (C) in tumor (*n* = 3 per group). Flow cytometric analysis of surface markers CD80 and CD86 on F4/80^+^ and CD11c^+^ cells in (D,E) peripheral blood, (F,G) spleen, (H,I) TDLNs, and (J,K) in tumors (*n* = 3 per group). (L) Schematic illustration of experimental studies. (M) Expression levels of MHC II and CD206 on F4/80^+^ cells, and (N) Ly6g on CD11b^+^ cells and MHC II on Ly6g^+^ cells in tumors by flow cytometry (*n* = 3 per group). Statistical significance was indicated by *p < 0.05, **p < 0.01, ***p < 0.001, and ****p < 0.0001, determined using one-way ANOVA with Tukey’s multiple comparisons test.

To evaluate APC maturation, we measured the expression of CD80 and CD86 as markers of mature macrophages and DCs. Both RMSN-PEG/PEI@OMVs and CpG@RMSN-PEG/PEI@OMVs induced significantly elevated expression of these markers in peripheral blood, spleen, TDLNs, and tumors compared to controls (Figures 4D–K and S2-1D–K). However, CD86 expression in splenic macrophages did not differ significantly among treatment groups (Figure 4F). After confirmation of APC activation, we examined the polarization status of tumor-associated macrophages and tumor-associated neutrophils, particularly the immunosuppressive M2 (CD206^+^) and N2 phenotypes. These subsets are known to impair T cell infiltration and promote angiogenesis and immunosuppressive pathways within the TME.^37,38^ We thus investigated whether CpG@RMSN-PEG/PEI@OMVs could counteract these effects (Figures 4L–N and S2-1L–O).

Seven days after two intravenous doses, tumors from CpG@RMSN-PEG/PEI@OMV-treated mice displayed the lowest proportion of CD206^+^ macrophages and the highest frequency of MHC II^+^ macrophages among all groups (Figure 4M), indicating a shift toward a proinflammatory M1 phenotype. We also analyzed tumor-infiltrating CD11b^+^Ly6G^+^ neutrophils, which have been implicated in antitumor immunity.^39^ Although total neutrophil numbers did not differ significantly among groups, the proportion of MHC II^+^ neutrophils was significantly higher in mice treated with RMSN-PEG/PEI@OMVs or CpG@RMSN-PEG/PEI@OMVs, with the latter showing the strongest effect (Figure 4N). Although neutrophils are typically considered terminally differentiated innate immune cells, emerging evidence suggests that MHC II^+^ neutrophils can engage in CD4^+^ T cell activation in cancer patients.^40,41^ In summary, CpG@RMSN-PEG/PEI@OMVs effectively promote M1 macrophage polarization and enhance APC antigen-presenting capacity, facilitating a proinflammatory TME. These changes may contribute to the initiation and maintenance of durable T cell-mediated antitumor immunity.

### 2.8 Enhancing T Lymphocyte Engagement in Tumors Using CpG@MSN-PEG/PEI@OMVs

To investigate how CpG@MSN-PEG/PEI@OMVs influence tumor-infiltrating T cells, we examined their activation profile following treatment. 24 h after a single dose, tumors from CpG@MSN-PEG/PEI@OMVs-treated mice exhibited significantly elevated IFNγ secretion by CD4^+^ T cells, exceeding levels observed in all other groups (Figures 5A and S2-1P). ELISpot analysis using splenocytes further confirmed enhanced IFNγ secretion in this group at the same time point (Figure 5B), consistent with previous observations.^20^ However, no significant activation of CD8^+^ T lymphocytes was detected in this early stage. Based on established T cell activation kinetics, naïve T cells require several days postpriming to differentiate and recirculate.^42^ We therefore extended our analysis to 7 days following two injections. At this later time point, mice treated with RMSN-PEG/PEI@OMVs or CpG@MSN-PEG/PEI@OMVs showed an increase in tumor-infiltrating IFNγ-producing CD8^+^ T cells compared to control and RMSN-PEG/PEI groups, although this increase did not reach statistical significance (Figures 5C and S2-1Q). To further evaluate local immune activation, IHC analysis of 4T1 tumor sections from CpG@MSN-PEG/PEI@OMVs-treated mice revealed an increased infiltration of CD8^+^ T cells and elevated levels of IFNγ, IL-6, and IL-1β in both tumors and TDLNs. Simultaneously, the immunosuppressive markers CD206 and Foxp3 were significantly reduced compared with all other groups (Figures 5D and S1-5, 6). Interestingly, IFNγ secretion by CD4^+^ T cells within the TME following MSN-PEG/PEI@OMV treatment remained at baseline levels, comparable to those in the control group at this time point (Figures 5E and S2-1R). This finding suggests a limitation of MSN-PEG/PEI@OMVs in sustaining prolonged inflammatory responses. In contrast, CD4^+^ T cells from CpG@MSN-PEG/PEI@OMVs-treated tumors maintained elevated levels of IFNγ production, indicating that the inclusion of CpG enhances T cell functionality under chronic inflammatory conditions. This was supported by both flow cytometry and IHC analyses. Further confirmation was obtained via ELISpot assays on day 23 using splenocytes from 4T1 tumor-bearing mice. T cells from the CpG@MSN-PEG/PEI@OMVs-treated group exhibited a higher frequency of IFNγ-producing spots (Figures 5F and S1-7), consistent with flow cytometry results (Figures 5E and S2-1R), demonstrating a more sustained systemic immune response.

**Figure 5:**
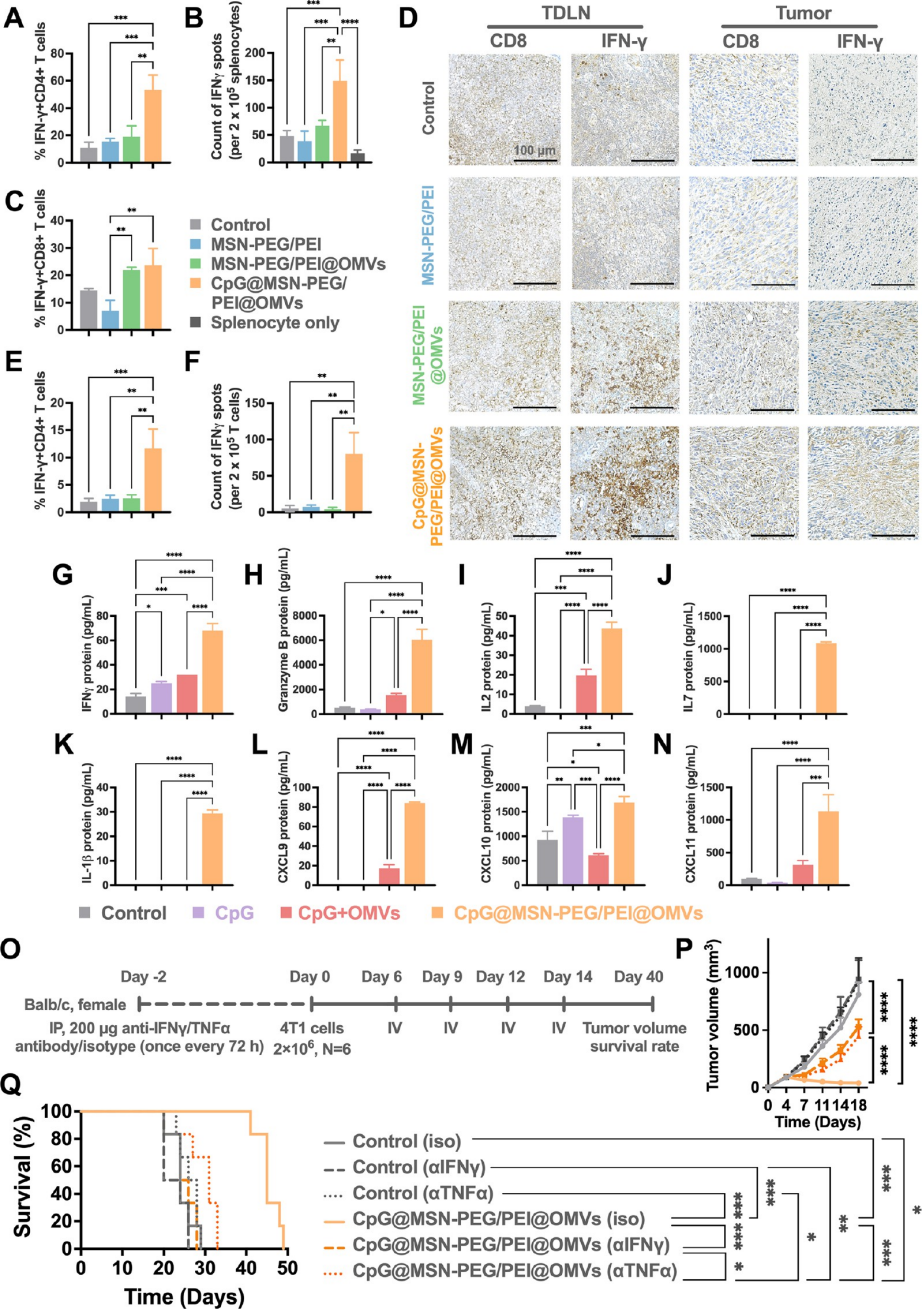
Role of CpG@MSN@PEG/PEI@OMVs in modulating adaptive immunity for enhanced tumor immunotherapy in vivo. (A) Quantitative flow cytometry analysis of IFNγ^+^CD4^+^ T cell expression in tumor 24 h postintravenous injection of various treatments (*n* = 3 per group). (B) ELISpot-based quantification of IFNγ secretion in splenocytes of mice receiving various treatments after 24 h (*n* = 3 per group). (C) Flow cytometry analysis of IFNγ^+^CD8^+^ and (E) IFNγ^+^CD4^+^ cell percentages in tumors 7 days after two intravenous injections of the indicated treatment (*n* = 3 per group). (D) IHC staining of CD8 T cells and IFNγ in tumor tissues isolated from BALB/c mice with 4T1 tumors, 7 days post two intravenous injections of the different treatments (*n* = 3 per group) (scale bar: 100 μm). (F) ELISpot-based quantification of IFNγ secretion in T lymphocytes isolated from splenocytes of mice receiving various treatments after 7 days (*n* = 3 per group). (G–N) Release of inflammatory cytokines and chemokines IFNγ, GrB, IL-2, IL-7, IL-1 beta, CXCL9, CXCL10, and CXCL11 in blood serum 7 days after two intravenous injections of the indicated treatments (*n* = 3 per group). (O) Schematic illustration of in vivo cytokine depletion. (P) Average tumor progression and (Q) survival of 4T1 tumor-bearing mice treated with PBS + isotype (gray), PBS + blocking antibodies against IFNγ^+^ (αIFNγ) (black), PBS + blocking antibodies against TNFα^+^ (αTNFα) (dotted black), CpG@MSN-PEG/PEI@OMVs + isotype (skin color), CpG@MSN-PEG/PEI@OMVs + αIFNγ (orange), and CpG@MSN-PEG/PEI@OMVs + αTNFα (dotted orange) (*n* = 6 per group). Statistical significance was indicated by *p < 0.05, **p < 0.01, ***p < 0.001, and ****p < 0.0001, determined using one-way ANOVA (A-N), two-way ANOVA with Tukey’s multiple comparisons test (P), or log-rank tests (Q).

Moreover, lymph node-like structures (LNS) were frequently observed adjacent to 4T1 tumors in mice 7 days after two intravenous doses of CpG@MSN-PEG/PEI@OMVs, but they were rarely seen in control or MSN-PEG/PEI@OMV-treated animals. Representative images of LNS are provided in Figure S1-8. Flow cytometric analysis of LNS from CpG@MSN-PEG/PEI@OMVs-treated mice revealed a complex immune cell composition, including CD45^+^ leukocytes (51.5%), CD138^+^B220^–^ plasma cells (16.9%), and CD4^+^ (23.4%) and CD8^+^ (7.5%) T cell subsets. Within these T cell populations, 17.8% of CD4^+^ and 21.6% of CD8^+^ T cells exhibited a central memory phenotype (CD44^+^CD62L^+^), while 27.7% of CD4^+^ and 10.8% of CD8^+^ T cells expressed an effector memory phenotype (CD44^+^CD62L^–^).

### 2.9 Systemic Cytokine Profiles and the Role of Activated T Cell Cytokines in CpG@MSN-PEG/PEI@OMVs Antitumor Strategies

Following the confirmation of enhanced IFNγ-secreting CD4^+^ and CD8^+^ T cell populations in tumors treated with CpG@MSN-PEG/PEI@OMVs, we next evaluated the systemic Th1 immune response. This direction was guided by the interplay between localized antitumor immunity and broader systemic immune modulation, which collectively determines immunotherapeutic efficacy.^43^ To assess this, we employed Quantibody multiplex ELISA arrays to analyze serum cytokines in 4T1 tumor-bearing mice treated intravenously with two doses of PBS, free CpG, CpG mixed with OMVs, or CpG@MSN-PEG/PEI@OMVs. As anticipated, OMV-based formulations induced elevated levels of proinflammatory cytokines, including TNFα, GM-CSF, Chemokine (C Motif) Ligand 1 (XCL1), IL-21, and IL-36, compared to the control and MSN-PEG/PEI groups (Figure S1-9).

Notably, as shown in Figure 5G–N, CpG@MSN-PEG/PEI@OMVs induced the highest expression levels of cytokines associated with cytotoxic T cell activity (IFNγ, Granzyme B), Th1 immune polarization (IL-2, IL-7, and IL-1β), and T cell recruitment (CXCL9, CXCL10, and CXCL11), compared with other treatment groups. Importantly, serum concentrations of cytokines linked to CRS (e.g., IL-6 and TNFα) remained within safe, physiological ranges, indicating a therapeutically favorable but nonpathologic immune activation profile. Having profiled systemic cytokine responses, we then focused on IFNγ and TNFα—two cytokines known to modulate immune surveillance and tumor control.^44,45^ To dissect their contribution to CpG@MSN-PEG/PEI@OMV-induced antitumor immunity, we performed cytokine depletion experiments in 4T1 tumor-bearing mice (Figure 5O). Strikingly, the neutralization of either IFNγ or TNFα substantially diminished the therapeutic efficacy of CpG@MSN-PEG/PEI@OMVs (Figure 5P), highlighting their essential roles in mediating the observed antitumor effects.

In the subset of CpG@MSN-PEG/PEI@OMVs-treated mice subjected to TNFα depletion, it is plausible that IFNγ-mediated immunostimulatory effects partially depend on TNFα-mediated maturation of APCs and T cells (CD4^+^ and CD8^+^), which may enhance IFNγ production and activity.^46,47^ As such, the TNFα deficiency in this group compromised tumor control despite the persistence of IFNγ secretion, underscoring the cooperative role of TNFα in facilitating optimal immune activation during treatment. Interestingly, our earlier flow cytometry analysis did not reveal a marked elevation in TNFα levels, potentially due to the limited immunological response induced by the two-dose regimen of CpG@MSN-PEG/PEI@OMVs. Consequently, TNFα fluctuations may have remained below the detectable thresholds.

Survival analysis (Figure 5Q) further showed that TNFα depletion had a minimal impact on overall survival in CpG@MSN-PEG/PEI@OMVs-treated mice. This may reflect the dominant role of IFNγ in controlling metastasis, particularly in breast cancer models, where TNFα has paradoxically been associated with enhanced tumor cell migration.^48,49^ As such, even in the presence of TNFα during IFNγ depletion, survival outcomes were not significantly improved. Collectively, these findings highlight the essential and distinct roles of both IFNγ and TNFα in regulating tumor progression following CpG@MSN-PEG/PEI@OMVs treatment, with IFNγ playing a particularly critical role in improving survival outcomes in this breast cancer model.

### 2.10 Long-Lasting Antigen-Specific Immunological Memory Induced by CpG@MSN-PEG/PEI@OMVs

To evaluate the long-term immunological memory elicited by CpG@MSN-PEG/PEI@OMVs, 4T1 tumor-bearing mice received two intravenous injections of each formulation on days 13 and 16. On day 23, tumors and spleens were harvested for flow cytometric analysis of memory T cell populations (Figure 6A). As shown in Figures 6B,C and S2-1S,T, both splenic central memory T cells (CD4^+^CD44^+^CD62L^+^) and tumor-infiltrating effector memory T cells (CD4^+^CD44^+^CD62L^–^) were significantly elevated in mice treated with MSN-PEG/PEI@OMVs or CpG@MSN-PEG/PEI@OMVs, relative to the control and MSN-PEG/PEI groups. To further confirm antigen-specific recall responses, ELISpot analysis of splenocytes on day 23 showed that CpG@MSN-PEG/PEI@OMVs-treated mice exhibited the highest frequency of IFNγ-secreting T cells following the ex vivo rechallenge with 4T1 cells. This finding supports the induction of antigen-specific immune memory by CpG@MSN-PEG/PEI@OMVs and demonstrates their ability to bridge innate and adaptive immune responses (Figure 6D).

**Figure 6:**
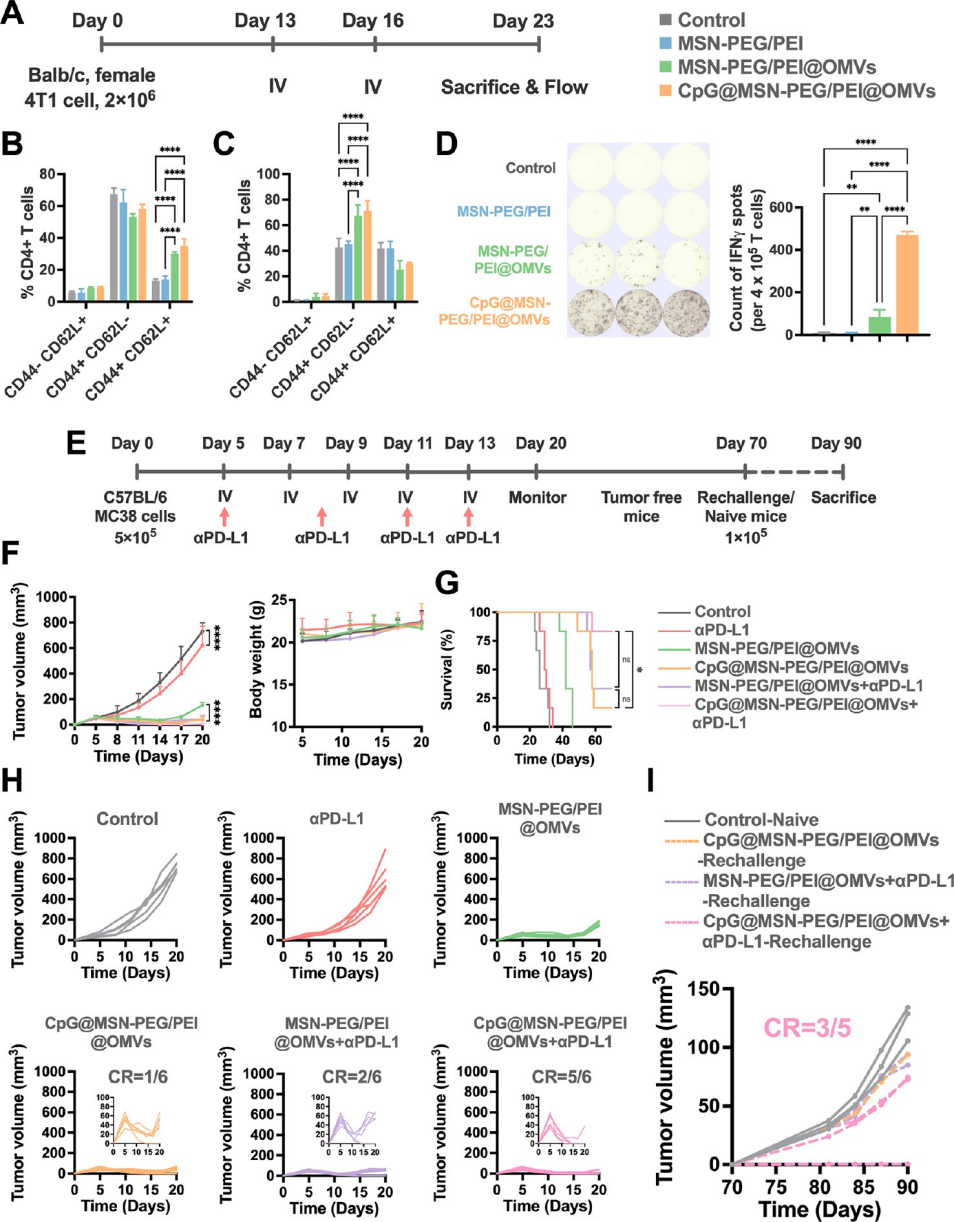
Sustained immune memory and tumor resistance induced by CpG@MSN-PEG/PEI@OMVs. (A) Schematic timeline of memory T cell response to the indicated treatment in 4T1 mouse tumor models. (B) Relative numbers of naïve (CD44^–^CD62L^+^), central memory (CD44^+^CD62L^+^), and effector memory (CD44^+^CD62L^–^) CD4+ T cell subsets in spleens and (C) tumors of 4T1 tumor-bearing mice following various treatment administrations by flow cytometry (*n* = 3 per group). (D) ELISpot-based quantification of IFNγ secretion in T cells from treated mouse splenocytes restimulated with 4T1 cells (*n* = 3 per group). (E) Schematic timeline of antitumor treatment efficacy and tumor rechallenge in MC38 mouse tumor models. (F) Average tumor volume curves and body weight after treatment. (G) Overall survival rates across a 70 day observation period (*n* = 6 per group). (H) Tumor progression in each mouse after the indicated treatments. (I) Tumor growth post MC38 rechallenge of naïve (*n* = 3), CpG@MSN-PEG/PEI@OMVs-treated mice (*n* = 1), MSN-PEG/PEI@OMVs with anti-PD-L1 antibody-treated mice (*n* = 2), and CpG@MSN-PEG/PEI@OMVs with anti-PD-L1 antibody-treated mice (*n* = 5). Statistical significance was indicated by *p < 0.05, **p < 0.01, ***p < 0.001, and ****p < 0.0001, determined using one-way ANOVA (B-D), two-way ANOVA with Tukey’s multiple comparisons test (F), or log-rank tests (G).

Given the robust IFNγ induction observed in tumors and serum (Figures 4A–G and 5D), and considering IFNγ′s role in upregulating PD-L1 on tumor cells,^50^ we next investigated whether CpG@MSN-PEG/PEI@OMVs could synergize with anti-PD-L1 checkpoint blockade. In the MC38 tumor model, mice received five intravenous treatments (starting on day 5, every 3 days), with anti-PD-L1 antibodies administered intraperitoneally on days 5, 8, 11, and 13 (Figure 6E). As shown in Figure 6F–H, while both MSN-PEG/PEI@OMVs and CpG@MSN-PEG/PEI@OMVs—alone or in combination with anti-PD-L1—delayed tumor growth compared to controls, the combination of CpG@MSN-PEG/PEI@OMVs and anti-PD-L1 antibody produced the most pronounced effect, achieving a complete tumor remission rate of 83.3% and significantly prolonging survival. Anti-PD-L1 monotherapy had negligible effects on progression-free survival with no significant improvement over PBS controls. All treatments were well tolerated, with no signs of acute toxicity or weight loss.

To assess the durability of memory immunity, surviving mice were rechallenged with MC38 tumor cells on day 70 post-treatment. Notably, mice previously treated with CpG@MSN-PEG/PEI@OMVs plus anti-PD-L1 antibody exhibited a 60% complete protection rate, whereas other treatment groups failed to suppress tumor regrowth (Figure 6I). These results demonstrate a strong synergistic interaction between CpG-based TLR9 activation and PD-L1 blockade, amplifying the durability and specificity of memory T cell responses.

Our findings are consistent with previous studies reporting that combined targeting of TLR9 and PD-L1 enhances tumor eradication and protects against rechallenge in MC38 models.^51^ Furthermore, clinical data support the therapeutic potential of TLR9 agonists, such as vidutolimod, in patients with anti-PD-1-resistant metastatic melanoma.^52^ Together, these results support the application of TLR9-based strategies in combination with an ICB for promoting durable, antigen-specific tumor immunity.

### 2.11 Modulating T Cell Exhaustion Phenotypes and Systemic Immunosuppressive Cytokines with CpG@MSN-PEG/PEI@OMVs in Mice with 4T1 Carcinoma

In chronic tumor environments, elevated expression of PD-1 and Tim-3 on CD8^+^ T cells is a hallmark of T cell exhaustion.^53^ To evaluate whether CpG@MSN-PEG/PEI@OMVs can reverse this dysfunctional phenotype, we examined PD-1 and Tim-3 expression on CD4^+^ and CD8^+^ T cells in 4T1 tumor-bearing mice (Figure 7A). Following two tail vein injections of the indicated treatments, mice receiving CpG@MSN-PEG/PEI@OMVs exhibited a marked reduction in PD-1^+^CD4^+^ and Tim-3^+^CD8^+^ T cell populations within tumors and TDLNs compared to the other groups. Specifically, CpG@MSN-PEG/PEI@OMVs-treated mice showed the lowest expression levels of both PD-1 and Tim-3 on tumor-infiltrating CD8^+^ T cells relative to the control, MSN-PEG/PEI, and MSN-PEG/PEI@OMV groups. Notably, PD-1 expression in CD8^+^ T cells from this group was nearly 7-fold lower than in mice treated with PBS or MSN-PEG/PEI (Figures 7B–E and S1-10).

**Figure 7:**
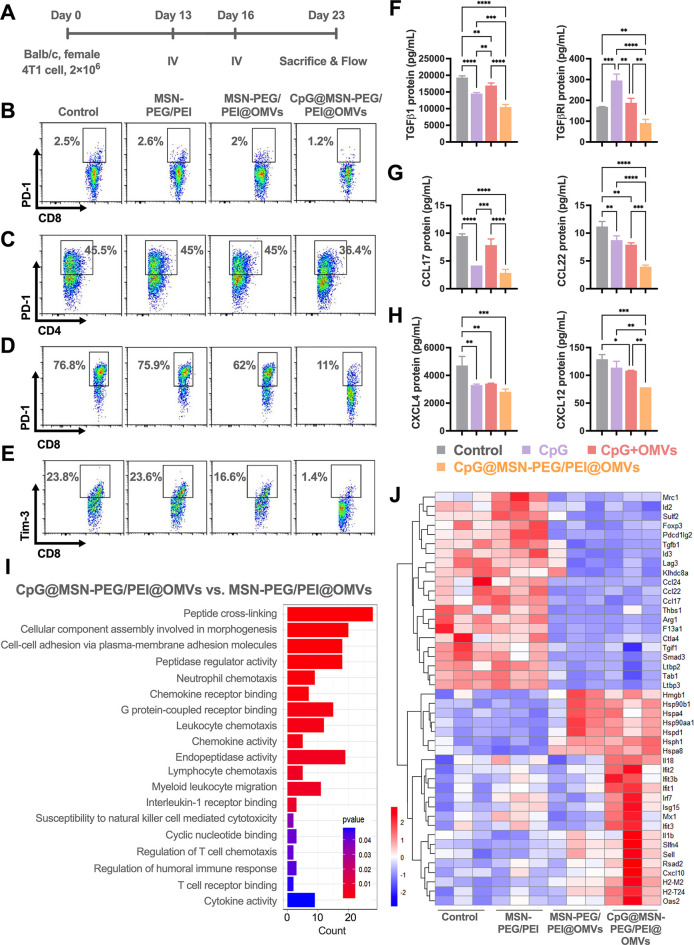
Impact of OMV nanohybrids on transcriptional alteration in TME. (A) Experimental framework of the 4T1 tumor model and TME evaluation. (B) Proportions of PD-1^+^CD8^+^ T cells in TDLNs analyzed by flow cytometry (*n* = 3 per group). (C–E) Proportions of PD-1^+^CD4^+^, PD-1^+^CD8^+^, and Tim-3^+^CD8^+^ T cells in tumors analyzed by flow cytometry (*n* = 3 per group). (F–H) Release of immunosuppressive cytokines and chemokines TGFB1, TGFBR1, CCL17, CCL22, CXCL4, and CXCL12 in blood serum (*n* = 3 per group). (I) Identification of significant GO process enrichment terms in TME from CpG@MSN-PEG/PEI@OMVs-treated mice as compared with MSN-PEG/PEI@OMV-treated mice (*n* = 3 per group). (J) Heatmap analysis of DEGs in tumor following treatment with PBS, MSN-PEG/PEI, MSN-PEG/PEI@OMVs, or CpG@MSN-PEG/PEI@OMVs as identified by RNA sequencing (*n* = 3 per group). Statistical significance was indicated by *p < 0.05, **p < 0.01, ***p < 0.001, and ****p < 0.0001, determined using one-way ANOVA with Tukey’s multiple comparisons test (B–H).

We next assessed the systemic immunosuppressive cytokine profile in 4T1 tumor-bearing mice following treatment. Serum levels of TGF-β pathway components (TGF-β1 and TGF-β receptor 1), chemokines associated with regulatory T cell (Treg) recruitment, including chemokine (C–C Motif) ligand 17 (CCL17) and ligand 22 (CCL22), and those promoting myeloid-derived suppressor cell (MDSC) trafficking (CXCL4 and CXCL12) were all significantly reduced in the CpG@MSN-PEG/PEI@OMVs group compared to controls (Figure 7F–H). Collectively, these findings demonstrate that CpG@MSN-PEG/PEI@OMVs attenuate T cell exhaustion phenotypes and suppress systemic immunosuppressive cytokine signals, contributing to functional antitumor immunity and mitigating exhaustion-driven tumor progression.

### 2.12 Transcriptomic Reprogramming of TMEs Induced by OMV Nanohybrids (MSN-PEG/PEI@OMVs or CpG@MSN-PEG/PEI@OMVs)

One of the major obstacles in cancer immunotherapy is the metabolic restriction within the TME, including glucose depletion and hypoxia, which severely impair TILs and diminish antitumor immune responses.^54,55^ Dysfunctional mitochondrial metabolism, reduced glycolytic activity, and impaired OXPHOS are hallmark features of exhausted T cells.^56–58^ Reversing these metabolic limitations is, therefore, essential for restoring effective antitumor immunity. To further investigate the intratumoral immune modulation observed following CpG@MSN-PEG/PEI@OMVs treatment—initially identified via flow cytometry—we conducted transcriptomic profiling of the treated tumors. We focused on global gene expression changes related to inflammatory signaling, immune checkpoint regulation, and immunosuppressive mechanisms within the TME. Mice bearing 4T1 tumors received two intravenous doses of each formulation, after which tumors were harvested for RNA sequencing (RNA-seq) analysis.

Principal component analysis (PCA) was performed to assess the transcriptomic variation among treatment groups, as shown in Figure S1-11. Differential gene expression analysis revealed that MSN-PEG/PEI@OMVs treatment resulted in 1620 differentially expressed genes (DEGs) relative to control and 1926 DEGs relative to MSN-PEG/PEI alone (Figure S1-12B,D). In contrast, CpG@MSN-PEG/PEI@OMVs treatment resulted in 1656 and 1638 DEGs compared to the control and MSN-PEG/PEI groups, respectively (Figure S1-12C,E). Notably, comparison between CpG@MSN-PEG/PEI@OMVs and MSN-PEG/PEI@OMVs alone revealed an additional 615 DEGs, underscoring the transcriptomic reprogramming driven by CpG incorporation (Figure S1-12F).

Gene ontology (GO) analysis of tumors treated with OMV nanohybrids versus control or MSN-PEG/PEI groups demonstrated significant enrichment in categories including cytokine activity, positive regulation of inflammatory response, myeloid leukocyte activation, and T cell activation/proliferation (Figure S1-13B,D). In contrast, CpG@MSN-PEG/PEI@OMVs-treated tumors exhibited unique enrichment in GO terms associated with humoral immune responses and leukocyte chemotaxis when compared to those of both control and MSN-PEG/PEI groups (Figure S1-13C,E). No such enrichment was observed in the OMV-only group compared to baseline, suggesting that CpG integration further amplifies immune-related transcriptional programs.

Further comparison of the TME from CpG@MSN-PEG/PEI@OMVs-treated mice versus those treated with MSN-PEG/PEI@OMVs revealed enrichment of GO terms related to peptide cross-linking, chemokine receptor binding, leukocyte and lymphocyte chemotaxis, natural killer (NK) cell-mediated cytotoxicity susceptibility, regulation of T cell chemotaxis, humoral immune response modulation, T cell receptor binding, and cytokine activity (Figure 7I).

To gain deeper insight into metabolic and immune pathway alterations induced by CpG@MSN-PEG/PEI@OMVs, we performed a Kyoto Encyclopedia of Genes and Genomes (KEGG) pathway enrichment analysis. This analysis identified notable enrichment in cytokine–cytokine receptor interactions, viral protein–cytokine receptor interactions, and complement and coagulation cascades when comparing CpG@MSN-PEG/PEI@OMVs to both control and MSN-PEG/PEI-treated groups (Figure S1-14).

Heatmap visualization of gene expression in 4T1 tumors treated with the OMV nanohybrids further confirmed substantial transcriptomic remodeling (Figure 7J). Compared to control and MSN-PEG/PEI groups, 23 genes were upregulated, and 21 genes were downregulated following OMV nanohybrid treatment. Among the upregulated genes, several were associated with damage-associated molecular patterns, including *Hmgb1*, *Hsp90b1*, *Hspa4*, *Hsp90aa1*, *Hspd1*, *Hsph1*, and *Hspa8*, as well as the pyroptosis-related cytokine gene *Il1b*.

In CpG@MSN-PEG/PEI@OMVs-treated tumors, a further increase was observed in 16 inflammation-associated genes, including the pyroptosis gene *Il18* and a suite of interferon-induced transcripts (*IFIT3B*, *IFIT2*, *IFIT1*, *IRF7*, *ISG15*, *MX1*, and *IFIT3*), consistent with earlier flow cytometry data indicating enhanced IFNγ secretion. Genes related to immune cell activation (*Slfn4*, *Sell*, *Rsad2*, *Cxcl10*, and *Oas2*)^59–62^ and antigen presentation (*H2-M2* and *H2-T24*) were also significantly elevated in the CpG@MSN-PEG/PEI@OMVs group relative to controls and MSN-PEG/PEI-treated mice.

Conversely, 21 genes were significantly downregulated in the TME following OMV nanohybrid treatment, including those involved in TGF-β signaling (*Smad3*, *Tab1*, *Ltbp3*, *Ltbp2*, *Thbs1*, *Tgfb1*, *Sulf2*, and *F13a1*);^63–68^ M2 macrophage-related genes (*Mrc1*, *Arg1*, *CCL24*, and *CCL22*);^69–71^ regulatory T cell-associated genes (*CCL17*, *CCL22*, *Foxp3*, and *Ctla4*);^72–75^ and T cell exhaustion-related transcripts (*Id2*, *Foxp3*, *Pdcd1lg2*, *Lag3*, and *Ctla4*).^76–79^ Hierarchical clustering highlighted distinct gene expression patterns in the OMV-treated groups compared to the control and MSN-PEG/PEI-treated mice, reinforcing the substantial immunomodulatory effects exerted by the OMV nanohybrids within the TME.

Gene set enrichment analysis (GSEA) further confirmed the enrichment of gene signatures related to humoral immunity in CpG@MSN-PEG/PEI@OMVs-treated tumors versus control and MSN-PEG/PEI-treated groups (Figure S1-15). Beyond local tumor effects, we also observed systemic immunomodulation within secondary lymphoid structures. Specifically, flow cytometry of spleens and TDLNs revealed a significant expansion of plasma cells (CD138^+^B220^–^) in mice treated with OMV nanohybrids compared with controls and MSN-PEG/PEI alone. Notably, CpG@MSN-PEG/PEI@OMVs further amplified this response beyond that of the OMVs alone (Figures S1-16 and S2-1U,V), suggesting enhanced B cell-mediated immunity. A higher proportion of plasma cells has been associated with prolonged relapse-free intervals in triple-negative breast cancer.^80^ B cells are increasingly recognized for their capacity to promote T cell immunity by producing cytokines and chemokines and differentiating into antibody-secreting plasma cells.^81^ These antibodies may directly recognize tumor-associated antigens, bind cellular targets, or mediate antibody-dependent cellular cytotoxicity via Fc receptor engagement, thereby facilitating antigen presentation and potentiating T cell activation.^82^ In summary, CpG@MSN-PEG/PEI@OMVs orchestrate transcriptional reprogramming within the TME, characterized by suppression of immunosuppressive and exhaustion-associated gene signatures, enhancement of inflammatory and interferon-mediated pathways, and induction of systemic humoral responses. These findings indicate a shift from an immunologically “cold” to “hot” tumor phenotype, supporting further investigation of this nanoplatform in cancer immunotherapy.

### 2.13 Upregulation of Mitochondrial OXPHOS in T Cells from TDLNs by CpG@MSN-PEG/PEI@OMVs Revealed through Single-Cell Transcriptomics (scRNA-Seq)

To elucidate the mechanism by which CpG@MSN-PEG/PEI@OMVs mitigate T cell exhaustion and enhance systemic antitumor immunity, we performed scRNA-seq on TDLNs harvested from 4T1 tumor-bearing mice treated with PBS (control), CpG mixed with OMVs, or CpG@MSN-PEG/PEI@OMVs (*n* = 3 per group, pooled for analysis; Figure 8A). After quality control filtering, a total of 18,196 cells were retained: 5001 from control, 6820 from CpG + OMVs, and 6375 from CpG@MSN-PEG/PEI@OMVs. Major immune cell types—including B cells, DCs, macrophages, NK cells, neutrophils, and T cells—were annotated using canonical markers (Figure 8B).^83^

**Figure 8:**
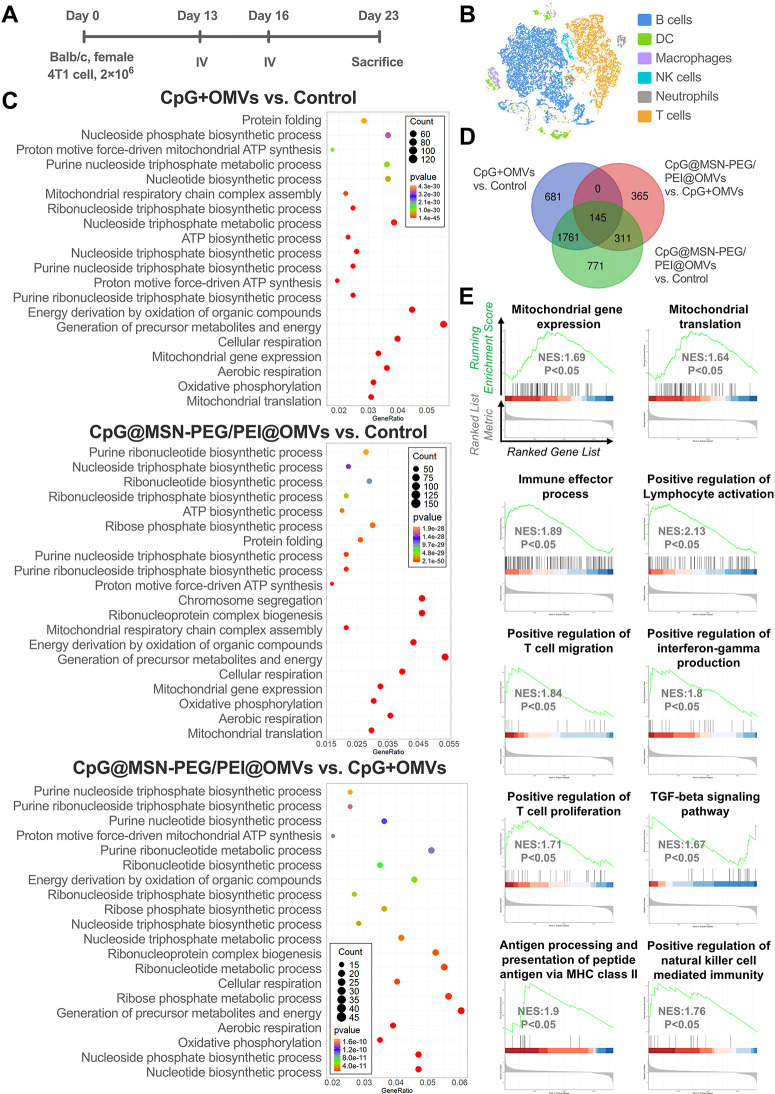
Impact of CpG@MSN-PEG/PEI@OMVs on transcriptional profiles in TDLNs. (A) Experimental framework of TDLNs collected on day 23 after 4T1 tumor-implanted mice (*n* = 3 per group) were intravenously administrated with various treatments on day 13 and day 16. (B) t-distributed stochastic neighbor embedding (t-SNE) plot of the detected immune cell subpopulations from groups of control, CpG mixed with OMVs, and CpG@MSN-PEG/PEI@OMVs in scRNA-seq data. Colored squares indicate cell types. (C) GO terms enrichment of biological process on significantly upregulated pathways between CpG mixed with OMVs versus control, CpG@MSN-PEG/PEI@OMVs versus control, and CpG@MSN-PEG/PEI@OMVs versus CpG mixed with OMVs. (D) Venn diagram of positive gene regulations in T cell subclusters separated from 6 clusters in t-SNE showing an overlap between control, CpG mixed with OMVs, and CpG@MSN-PEG/PEI@OMVs. (E) GSEA pathway plot related to mitochondrial activity and immunological activation and response in T cell clusters from CpG@MSN-PEG/PEI@OMVs-treated mice compared with T cell clusters from PBS-treated mice as identified by scRNA-seq integration data set. NES, normalized enrichment score.

GO enrichment analysis revealed distinct transcriptional responses in myeloid populations following the CpG@MSN-PEG/PEI@OMVs treatment. In DCs, genes involved in energy production, mitochondrial respiration, and oxidative metabolism were significantly upregulated (Figure S1-17). Macrophages showed enhanced expression of genes related to oxidative stress responses and chromatin remodeling (Figure S1-18), while neutrophils exhibited changes in transcripts associated with protein–DNA binding and chromosome organization (Figure S1-19). Notably, DCs from the CpG@MSN-PEG/PEI@OMVs group expressed higher levels of cDC1-related markers (*Batf3*, *Xcr1*, *Cxcl9*, and *CD86*) and MHC class II genes (*H2-Aa*, *H2-Eb1*, *H2-M3*, *H2-Q7*, *H2-DMb1*, *H2-DMb2*, *H2-Ab1*, and *H2-DMa*) (Figure S1-20).

In lymphoid compartments (B, NK, and T cells), CpG@MSN-PEG/PEI@OMVs treatment promoted transcriptional signatures linked to mitochondrial function and OXPHOS, including ATP synthesis and energy metabolism (Figures 8C and S1-21, 22). NK cells also showed an elevated expression of genes involved in immune activation and cytotoxicity. Both B and T cells demonstrated transcriptional evidence of increased mitochondrial activity, which is a hallmark of cellular longevity and effector readiness.

In T cells specifically, significant upregulation of electron transport chain and OXPHOS-related genes—*Atp6v1g1*, *Cox5a*, *Cox7b*, *Cox8a*, *Ndufs4*, *Ndufa5*, *Atp5j2*, *MPC1*, and *MPC2*—was observed (Figures 8D and S1-23, 24).^56^ This metabolic reprogramming was accompanied by downregulation of exhaustion-associated NR4A transcription factors (*Nr4a1–3*)^84^ and increased expression of cytotoxic markers (*CD8a*, *Nkg7*, and *Cst7*), suggesting revitalization of effector T cell function. We hypothesize that the OMV-mediated delivery of bacterial PAMPs (e.g., LPS) in combination with CpG-ODN primes DCs to provide enhanced metabolic and functional support to T cells, fostering a shift from exhausted to memory/effector phenotypes (Figures S1-23, 24). Additionally, T cells from CpG@MSN-PEG/PEI@OMVs-treated mice expressed elevated levels of effector genes (*CD8a*, *CD8b*, *Nkg7*, *Cst7*, and *Lgals*)^85,86^ and stem-like T cell markers (*Slamf6*, *Gpr183*, *Ly6e*, *Ltb*, and *Myb*),^85,87^ indicating the emergence of a metabolically active, cytotoxic, and memory-prone T cell population.

To validate broader pathway engagement, we conducted GSEA. CpG + OMV-treated TDLNs exhibited enrichment in biological processes (BP), such as adaptive immune response, lymphocyte activation, and B cell-mediated immunity. In contrast, the CpG@MSN-PEG/PEI@OMVs group showed significant enrichment in mitochondrial gene expression, immune effector activity, T cell migration/proliferation, MHC class II-mediated antigen presentation, and positive regulation of IFNγ production, among others (Figures 8E and S1-25). These results indicate that CpG@MSN-PEG/PEI@OMVs reprogram T cells toward an OXPHOS-dominant phenotype, supporting both metabolic and immunological changes at the single-cell transcriptome level.

## 3 Conclusion

In this study, we evaluated an OMV-coated, CpG adjuvant-loaded MSN system as a nanotherapeutic strategy for cancer immunotherapy. Our results demonstrated that MSN-PEG/PEI@OMVs markedly enhanced accumulation in both tumors and TDLNs compared to uncoated OMVs. Systemic administration of CpG@MSN-PEG/PEI@OMVs effectively suppressed tumor growth and prevented lung metastasis primarily by promoting APC maturation, inducing M1 macrophage polarization, and activating IFNγ-secreting CD4^+^ and CD8^+^ T lymphocytes. This approach also elicited long-lasting, tumor antigen-specific immunological memory.

When combined with anti-PD-L1 therapy, CpG@MSN-PEG/PEI@OMVs successfully eliminated most established solid tumors in MC38 models, leading to durable cures and robust protection against the rechallenge. Mice treated with CpG@MSN-PEG/PEI@OMVs exhibited reduced expression of PD-1 and Tim-3 on tumor-infiltrating CD8^+^ T cells, as well as lower systemic levels of immunosuppressive cytokines, including TGF-β family members (TGF-β1 and TGF-β receptor 1) and chemokines responsible for regulatory T cell recruitment (CCL17 and CCL22). These immune-modulating effects collectively enhance the therapeutic efficacy of the checkpoint blockade.

Transcriptomic profiling revealed that CpG@MSN-PEG/PEI@OMVs reprogrammed the TME by downregulating immune checkpoint genes and immunosuppressive mediators while upregulating mitochondrial OXPHOS-related genes in T cells residing in TDLNs. Single-cell RNA sequencing further confirmed increased expression of *Atp6v1g1*, *Cox5a*, *Cox8a*, *Ndufs4*, and *Atp5j2*, accompanied by decreased expression of exhaustion-associated transcription factors (*Nr4a1–3*) and elevated effector markers such as *CD8a* and *Nkg7*. These findings indicate a metabolic shift from glycolysis toward OXPHOS, likely driven by TLR9/MyD88-mediated AMPK/mTORC1 signaling, thereby restoring mitochondrial function and promoting sustained T cell effector functionality under immunosuppressive conditions.

In conclusion, the CpG@MSN-PEG/PEI@OMVs system represents a strategy that integrates innate immune activation with a durable adaptive memory. By simultaneously remodeling the immunosuppressive TME and reconditioning T cell metabolism, this platform supports efforts to overcome barriers in cancer immunotherapy and contributes to applications aimed at achieving sustained, systemic antitumor immunity.

## 4 Experimental Section

### 4.1 Materials

3-Aminopropyltrimethoxysilane (APTMS, 95%), tetraethyl orthosilicate (TEOS, 98%), ammonium hydroxide (ACS reagent, 28–30%), and cetyltrimethylammonium bromide (CTAB, >99%) were obtained from ACROS Organics. 2-[Methoxy­(polyethyleneoxy)­6-9propyl]­trimethoxysilane (PEG-silane, MW 460–590 g/mol) and trimethoxysilylpropyl-modified polyethylenimine (PEI-silane, 50%, MW 1500–1800 g/mol) were purchased from Gelest. Rhodamine B isothiocyanate (mixed isomers, RITC), paraformaldehyde (PFA), Triton X-100, urea, acrylamide (≥99%, HPLC grade), *N*,*N*′-methylenebis­(acrylamide), and sodium dodecyl sulfate (SDS) were acquired from Sigma-Aldrich. A 22-mer unmethylated cytosine-phosphate-guanine oligodeoxynucleotide (CpG-ODN, 5′-TCGTCGTTTTCGGCGCGCGCCG-3′; natural phosphodiester backbone) was obtained from InvivoGen. The Cell Counting Kit-8 (CCK-8) was obtained from Dojindo. Percoll was purchased from GE Healthcare. PBS (10×) was obtained from UniRegion Bio-Tech. 2,2′-Azobis­[2-(2-imidazolin-2-yl)­propane] dihydrochloride (VA-044), 4′,6-diamidino-2-phenylindole (DAPI, 1 mg/mL), Cimarec digital stirring hot plate, Tris buffer (1 M, pH 8.0, RNase-free), and potassium acrylate (KA) were sourced from Thermo Fisher. No further purification was conducted on any reagent prior to use. Round coverslips (15 mm) were purchased from Marienfeld. Sixty millimeter plastic culture dishes were obtained from Alpha Plus. A hybridization oven was acquired from Bioman, and scalpels were purchased from Aesculap. Ultrapure deionized water was prepared using a Millipore Milli-Q Plus system.

### 4.2 Cell Lines

B16-F10 (mouse melanoma), MC38 (mouse colon carcinoma), 4T1 (mouse mammary carcinoma), and 4T1-Luc (luciferase-expressing 4T1) cell lines were obtained from the American Type Culture Collection (ATCC, Manassas, VA). The RAW 264.7 murine macrophage-like cell line was sourced from the Food Industry Research and Development Institute, Taiwan (BCRC 60001). Cells were cultured in either Dulbecco’s modified Eagle’s medium (DMEM) or RPMI 1640 medium (Gibco), supplemented with 10% fetal bovine serum (FBS; Gibco) and 100 U/mL penicillin–streptomycin (Gibco). MC38 cells were additionally cultured with 1 mM HEPES and 0.1 mM nonessential amino acids. All cultures were maintained at 37 °C in a humidified incubator with 5% CO_2_. When 60–70% confluency was reached, cells were passaged using 0.25% trypsin (Hyclone).

### 4.3 Synthesis of RMSN-PEG/PEI NPs

PEGylated RMSNs (∼25 nm in diameter) were synthesized using a previously reported soft-templated protocol.^88^ In brief, 0.29 g of CTAB (surfactant) was dissolved in 150 mL of aqueous ammonia (0.128 M) and stirred at 60 °C for 15 min in a water bath. Subsequently, 2.5 mL of APTMS-conjugated RITC was added, followed by dropwise addition of 2 mL of 0.88 M TEOS in 99.5% ethanol under continuous stirring for 1 h. Surface modification was then performed by adding 20 μL of PEI-silane and 550 μL of PEG-silane (both prediluted in ethanol), followed by 30 min of additional stirring. The NPs were aged at 50 °C overnight and further subjected to sequential hydrothermal treatments at 70 and 90 °C for 24 h each. After synthesis, the particles were washed multiple times with ethanol. To remove CTAB surfactant, ion exchange was initiated by adding concentrated hydrochloric acid (HCl, 37%) and incubating at 60 °C. The final RMSN-PEG/PEI NPs were stored in 99.5% ethanol until use.

### 4.4 Isolation of OMVs

OMVs were isolated from E. coli BL21 (DE3) ΔLpp (lipoprotein-deficient) strains. Bacteria were cultured in LB medium at 37 °C with shaking at 160 rpm overnight. Following incubation, cultures were centrifuged at 6000 rpm for 40 min at 4 °C to remove cells. The resulting supernatants were filtered through a 0.45 μm MCE membrane (ChromTech, #MJM4547) to eliminate residual bacterial contaminants. Subsequent purification involved ultrafiltration using a 100 kDa molecular weight cutoff membrane, allowing OMVs to be retained in PBS. The filtrates were then concentrated via ultracentrifugation at 45,200 rpm for 3 h at 4 °C. The resulting OMV pellets were resuspended in PBS and stored at −80 °C. To quantify the OMV protein content, we used the Pierce BCA Protein Assay Kit (Thermo Fisher), following the manufacturer’s protocol.

### 4.5 Characterization of Hybrid NPs

For morphological and structural analysis, samples were deposited onto carbon-coated copper grids and imaged using a Hitachi H-7100 TEM operating at 100 kV. Negative staining was performed separately for the OMVs and RMSN-PEG/PEI@OMVs using 2% uranyl acetate for 60 s. Excess stain was removed with filter paper, followed by air drying for 10 min prior to TEM observation.

Particle size distributions were determined using ImageJ software based on TEM images. Protein profiles of MSN-PEG/PEI@OMVs were evaluated by SDS-PAGE using a Mini-PROTEAN Tetra System (Bio-Rad Laboratories). The hydrodynamic diameter and zeta potential of RMSN-PEG/PEI, OMVs, RMSN-PEG/PEI@OMVs, and CpG-loaded RMSN-PEG/PEI@OMVs were measured via DLS using a Zetasizer Nano ZS (Malvern Instruments, UK). Particle concentration and distribution were analyzed using NP tracking analysis on a NanoSight NS300 system (Malvern Ltd.) equipped with 488 and 532 nm lasers. All measurements were performed in PBS, and each sample was analyzed in triplicate to calculate the mean particle size, zeta potential, and concentration.

### 4.6 MSN-PEG/PEI@OMV Formation and CpG Cargo Encapsulation

To fabricate MSN-PEG/PEI@OMVs, MSN-PEG/PEI particles were mixed with OMVs and subjected to 11 passes of extrusion through a 50 nm polycarbonate membrane (Avanti Polar Lipids) using a miniextruder in 1× PBS buffer (pH 7.4).

To evaluate their potential as CpG-delivering nanotherapeutics and compare their efficacy with free CpG-ODN or uncoated OMVs, CpG@MSN-PEG/PEI@OMVs were prepared based on electrostatic interaction between negatively charged CpG-ODN and positively charged PEI-functionalized MSNs. Briefly, CpG-ODN was gently mixed with MSN-PEG/PEI at an optimized mass ratio in distilled water, followed by incubation at room temperature for 4 h. To remove unbound CpG-ODN and concentrate the formulation, the mixture was filtered through 100 kDa MWCO ultrafiltration membranes. The retentate was repeatedly washed with distilled water to ensure complete removal of free CpG-ODN. The concentration of encapsulated CpG was quantified using a NanoDrop 2000c UV–Vis spectrophotometer (Thermo Scientific, Waltham, MA, USA). Encapsulation capacity (EC %) and encapsulation efficiency (EE %) were calculated using the following equations:

- EC (%) = (mass of encapsulated CpG/mass of total MSN-PEG/PEI@OMVs) × 100
- EE (%) = (mass of encapsulated CpG/mass of CpG initially added) × 100

### 4.7 Cell Viability Analysis

To evaluate the cytotoxicity of MSN-PEG/PEI@OMVs, RAW 264.7 cells were seeded in 96-well plates at a density of 1 × 10^4^ cells/well in DMEM supplemented with 2% FBS and incubated overnight. The following day, cells were treated with various concentrations (250, 500, 750, and 1000 μg/mL) of MSN-PEG/PEI, OMVs, or MSN-PEG/PEI@OMVs diluted in the culture medium and incubated at 37 °C for 24 h. After treatment, cells were rinsed three times with PBS and subjected to cell viability analysis using the CCK-8 assay (Dojindo Laboratories, Japan), following the manufacturer’s instructions. Briefly, the culture medium containing 10% CCK-8 reagent was added and incubated at 37 °C for 4 h. Absorbance was then measured at 450 nm by using a microplate reader. Cell viability was expressed as a percentage relative to that of untreated control cells.

### 4.8 Cellular Internalization Analysis

RAW 264.7 cells were plated in 6-well plates at a density of 2 × 10^5^ cells/well and incubated at 37 °C in a 5% CO_2_ atmosphere for 24 h. The cells were then exposed to 250, 500, 750, or 1000 μg/mL of RMSN-PEG/PEI, CellMask Deep Red-labeled OMVs (NIR-OMVs), or RMSN-PEG/PEI@NIR-OMVs for 24 h. After incubation, cells were washed three times with cold PBS and imaged using an IX-71 fluorescence microscope. Subsequently, cells were detached using trypsin, centrifuged at 800 rpm for 3 min, resuspended in PBS, and analyzed by flow cytometry (Agilent NovoCyte) to assess NP uptake based on fluorescence intensity.

### 4.9 In Vitro BMDC Isolation and Maturation Analysis

BMDCs were isolated from BALB/c mice. Bone marrow was flushed from femurs and tibiae using a 26-gauge needle and syringe, filtered through a 70 μm strainer (Fisher Scientific), and subjected to erythrocyte lysis using RBC Lysis Buffer (BioLegend). After washing with PBS, 5 × 10^6^ bone marrow cells were cultured in 10 cm dishes containing DMEM supplemented with 10% FBS, 100 U/mL penicillin–streptomycin, and 20 ng/mL recombinant murine GM-CSF. The culture medium (with cytokine) was refreshed every 2–3 days by replacing half of the volume.

On day 7, loosely adherent and nonadherent cells were harvested as immature BMDCs for maturation assays. To assess maturation, 5 × 10^5^ BMDCs were seeded into 6-well plates and treated with MSN-PEG/PEI (250 μg/mL), OMVs (0.5 μg/mL), or CpG@MSN-PEG/PEI@OMVs (CpG: 0.54 μg/mL) and incubated at 37 °C for 24 h. After treatment, cells were washed with PBS, gently scraped, and stained with FITC-conjugated antimouse MHC class II (clone M5/114.15.2; BioLegend) and APC-conjugated antimouse CD86 (clone GL-1; BioLegend). Expression of surface markers was analyzed via flow cytometry to evaluate BMDC maturation.

### 4.10 Animals

Female BALB/c and male C57BL/6 mice were obtained from the BioLASCO Experimental Animal Center (Taipei, Taiwan). All mice used in this study were between 6 and 8 weeks of age at the start of the experiments and were maintained under specific pathogen-free conditions. Animals were housed in a 12 h light/dark cycle at the Taipei Medical University Laboratory Animal Center with free access to food and water. All procedures were approved by and conducted in accordance with the guidelines set by the Institutional Animal Care and Use Committee (IACUC) of Taipei Medical University.

### 4.11 In Vivo Biosafety Investigation

To evaluate systemic biosafety, BALB/c mice were subcutaneously inoculated with 2 × 10^6^ 4T1 cells in the left flank. Mice received intravenous injections of the indicated formulations on days 13 and 16. Blood samples were collected via cardiac puncture on days 14 and 30, followed by serum separation by centrifugation at 3000 rpm for 10 min at 4 °C. CBCs and serum biochemistry tests were performed at the Taipei Medical University Laboratory Medicine Center. Age-matched healthy mice served as controls. On day 13, rectal temperature was monitored every 10 min for 1 h postinjection and again at 24 h using a rectal thermocouple probe (Bio-Cando Biotechnology Inc., Taipei, Taiwan) until readings stabilized. For histopathological examination, major organs including the heart, liver, spleen, lungs, and kidneys were harvested and subjected to hematoxylin and eosin (H&E) staining to assess potential tissue toxicity.

### 4.12 In Vivo Imaging

To assess biodistribution, BALB/c mice (*n* = 4) bearing 4T1 tumors (∼50 mm^3^, established 5 days after subcutaneous inoculation of 2 × 10^6^ cells) received intravenous injections of RMSN-PEG/PEI, NIR-OMVs, or RMSN-PEG/PEI@NIR-OMVs. After 24 h, mice were euthanized by cardiac perfusion using 4 mL of PBS containing 0.15% EDTA. Tissues including tumors, TDLNs, axillary and inguinal lymph nodes, and major organs (heart, liver, spleen, lungs, and kidneys) were collected. Fluorescence images were acquired using an IVIS system (Xenogen IVIS-200), and the NIR signal intensity was quantified and normalized to organ weight.

### 4.13 4X-Potassium Acrylate (KA) Expansion Microscopy

Samples fixed in 4% paraformaldehyde were preincubated in a 4× potassium acrylate-based expansion microscopy (4X-KA-ExM) gelling solution for 2 days at 4 °C, as previously described.^89^ The gelling solution was freshly prepared by combining the stock monomer solution with 1% VA-044 (initiator). For gelation, 200 μL of this solution was added between two 15 mm coverslips in a gelation chamber and incubated at 37 °C for at least 2 h to allow complete solidification. Excess gel was trimmed using a scalpel, and embedded samples were transferred into denaturation buffer (200 mM SDS and 20 mM Tris) and incubated at 70 °C with gentle shaking for 2 days. After denaturation, gels were washed three times in 1× PBST (PBS + 0.2% Triton X-100) at 37 °C for 2 h each, followed by two washes in 1× PBS at room temperature for 30 min. After final trimming, samples were stained with DAPI (1:3000 in PBS) at room temperature for 24 h. Poststaining, gels were briefly rinsed in 1× PBS and incubated in ddH_2_O for water expansion. Water was refreshed three times for 30 min each on a shaker. Fully expanded gels were stored in ddH_2_O at room temperature prior to light sheet imaging.

### 4.14 Sample Mounting and Light Sheet Imaging

Large-volume expanded tumors and whole lymph nodes were imaged using a custom-built scanning beam light sheet microscope at the Light Sheet Microscopy Core Facility of Academia Sinica. Samples were mounted on a 3D-printed imaging holder, as previously described.^89^ The camera exposure time was set to 85 ms, and laser illumination was controlled via an Arduino board, which relayed the camera’s “exposure all” signal to the laser control unit. A pair of 4× Olympus NA 0.28 objectives was used for excitation, and a 10× Olympus XLPLN10XSVMP NA 0.6 objective, combined with a 150 mm tube lens, was used for detection, yielding a final *xy* resolution of 0.78 μm/pixel. For high-resolution imaging with the Zeiss Lightsheet Z.1 system, expanded gels were trimmed into 5 × 10 mm rectangles, and the 5 mm edge was vertically mounted on a poly-l-lysine (0.01%)-coated rectangular coverglass. The coverglass was then glued to a T-shaped imaging holder and allowed to set for ∼3 min before being immersed in ddH_2_O in the imaging chamber. The tissue side was oriented toward the detection objective. Images were acquired at 1920 × 1920 pixels, 16 bit resolution, using 0.5–1× zoom, with 10×/0.2 illumination objectives and a W Plan-Apochromat 20×/1.0 detection objective. Excitation was performed using 405 and 561 nm lasers at 3% and 30% power, respectively, to image DAPI-stained nuclei and RMSN-PEG/PEI signals. Two separate tracks were used for each channel, with z-stack-based switching. A laser blocker (LBF 405/488/561/640) and SBS LP560 emission filter with BP 575–615 nm range were applied. Exposure time was 99.9 ms, and the light sheet thickness was set at 3.07 μm. Z-stack images were acquired in continuous mode with a z-step size ranging from 1 to 3 μm.

### 4.15 Antitumor Therapeutic Study

To establish subcutaneous tumors, female BALB/c mice were injected with 1 × 10^6^ 4T1 cells suspended in 50 μL of PBS into the left flank. Once tumors reached a volume of approximately 250–300 mm^3^, mice were randomly divided into four groups (*n* = 6 per group): (1) control (PBS), (2) RMSN-PEG/PEI (200 mg/kg), (3) RMSN-PEG/PEI@OMVs (OMVs: 8 μg/mouse total protein), and (4) CpG@RMSN-PEG/PEI@OMVs (CpG: 8.6 μg/mouse). Intravenous injections were administered on days 13 and 16. Tumor volumes were measured using digital calipers and calculated using the formula (width^2^ × length × 0.5).^90^ Body weight was also monitored every 3 days. On day 30, primary tumor weights were recorded. IVIS was conducted on days 6, 17, 27, and 30 to monitor tumor progression and metastasis. At the experimental end point, mice were euthanized with a combination of Zoletil and Rompun. Primary tumors were excised for imaging, and lungs were inflated with 15% India ink in 10% buffered formalin via intratracheal injection. Lungs were fixed in 4% paraformaldehyde and subjected to histological examination using H&E staining to assess metastatic burden.

### 4.16 Lung Metastasis Models

For the metastatic lung model, male C57BL/6 mice were intravenously injected with 1 × 10^6^ B16-F10 melanoma cells suspended in 200 μL of PBS. On days 4 and 8 postinoculation, mice received intravenous injections of MSN-PEG/PEI, MSN-PEG/PEI@OMVs, or CpG@MSN-PEG/PEI@OMVs, respectively. The control group received PBS. On day 15 following tumor cell injection, mice were euthanized under anesthesia with Zoletil 50 and Rompun (intraperitoneal), and lungs were harvested for evaluation. Visible melanoma nodules on the lung surface were counted under a stereomicroscope to quantify metastatic burden.

### 4.17 Histological Evaluation

Tumor tissues and TDLNs were fixed in 4% paraformaldehyde at room temperature for 24 h, followed by paraffin embedding. Tissue sections (5 μm thick) were deparaffinized, rehydrated, and incubated with peroxidase blocking solution at room temperature for 20 min. Antigen retrieval was performed by heating the sections in citrate buffer (pH 6.0) for 30 min. After cooling, sections were blocked with ImmunoBlock for 1 h at room temperature. Following PBS rinses, sections were incubated for 4 h with primary antibodies against the following targets: CD4 (clone RBT-CD4, BioSB), CD8 (clone C8/144B, BioSB), IFNγ (clone RBT-CD4, Servicebio), TNFα (clone 1F6, Bioss), IL-6 (clone RBT-CD4, Bioworld), IL-1beta (clone RBT-CD4, BioSS), CD206 (clone RBT-CD4, ACE Biolabs), or foxp3 (clone RBT-CD4, GeneTex). A horseradish peroxidase-conjugated rabbit probe (Toson Technology, Hsinchu, Taiwan) was applied for 30 min at room temperature. Color development was achieved using 3,3′-diaminobenzidine (1:50 dilution, Toson Technology), followed by hematoxylin counterstaining for 5 min. After dehydration, the slides were mounted and examined microscopically. Quantitative analysis of immunohistochemistry (IHC) was conducted using ImageJ software by two independent, blinded investigators. Immunoreactive cell areas were used for semiquantitative scoring. For immunofluorescence staining, lung tissues were fixed overnight in 10% formalin and cryoprotected in 30% sucrose in PBS at 4 °C overnight. After equilibration, tissues were embedded in an optimal cutting temperature compound, and 7 μm cryostat sections were prepared. Slides were blocked for 2 h at room temperature in 10% FBS/TBST (PBS with 0.2% Triton X-100), followed by overnight incubation with anti-Ki67 antibody (LifeSpan). The sections were then incubated with Alexa Fluor 488-conjugated secondary antibody (Invitrogen) and counterstained with DAPI. Fluorescence imaging was performed using an inverted fluorescence microscope (Olympus IX71).

### 4.18 Flow Cytometry

Peripheral blood samples were collected into EDTA-coated tubes, and PBMCs were isolated via density gradient centrifugation using Histopaque-1077 (Sigma-Aldrich, Munich, Germany) at 400*g* for 30 min at room temperature. Cells from the buffy coat layer were collected and washed three times with PBS. Red blood cells were lysed using RBC lysis buffer at 4 °C for 5 min, and the reaction was quenched with RPMI-1640 medium supplemented with 10% FBS. Cells were then blocked in flow staining buffer containing TruStain FcX anti-CD16/32 antibody (clone 93; BioLegend) for 15 min on ice to prevent nonspecific binding.

Tumors, TDLNs, and spleens were excised, weighed, and chopped into 2–4 mm fragments. Tumor tissues were enzymatically digested using a Tumor Dissociation Kit (Miltenyi Biotec) for 1 h at 37 °C. All samples were passed through 100 μm strainers (Falcon) and treated with RBC lysis buffer at 4 °C for 5 min. After the reaction was quenched with 10% FBS-RPMI medium, cells were centrifuged at 800*g* for 5 min. To isolate tumor-infiltrating leukocytes, cell suspensions were overlaid onto Percoll and centrifuged at 800*g* for 30 min with the brake off. The leukocyte-rich interphase was collected, washed with PBS, and blocked with TruStain FcX anti-CD16/32 antibody at 4 °C for 15 min. The following fluorophore-conjugated antibodies (BioLegend) were used for surface marker staining: Myeloid and APC markers: FITC-anti-CD11c (clone N418), PerCP-anti-F4/80 (clone BM8), PE-Cy7-anti-CD11b (clone M1/70), APC-Cy7-anti-Ly6g (clone 1A8), FITC-anti-Ly6g (clone 1A8), APC-anti-MHC class II (clone M5/114.15.2), APC-anti-CD86 (clone GL-1), PE-Cy7-anti-CD80 (clone 16-10A1), and PE-anti-CD206 (clone C068C2); T cell markers: PerCP-anti-CD4 (clone GK1.5), FITC-anti-CD8 (clone 53–6.7), APC-anti-CD62L (clone MEL-14), APC-anti-PD-1 (clone RMP1-30), and APC-anti-Tim-3 (clone RMT3-23); B cell and plasma cell markers: APC-anti-CD138 (clone 281-2) and FITC-anti-B220 (clone RA3-6B2); and stem-like and activation markers: PE-anti-CD44 (clone IM7) and APC-anti-CD24 (clone M1/69). Cells were stained in the dark at 4 °C for 30 min using flow staining buffer (BioLegend), followed by two PBS washes. For intracellular cytokine staining, cells were fixed and permeabilized according to standard protocols and then stained with APC-antimouse IFNγ (clone XMG1.2, BioLegend). All samples were analyzed on an Agilent NovoCyte flow cytometer. Data were acquired and analyzed by using NovoExpress software.

### 4.19 In Vivo Depletion Experiments

To evaluate the role of IFNγ and TNFα in mediating the therapeutic efficacy of CpG@MSN-PEG/PEI@OMVs, female BALB/c mice were subcutaneously inoculated in the left flank with 2 × 10^6^ 4T1 cells suspended in 50 μL of PBS. Mice received intravenous injections of MSN-PEG/PEI (200 mg/kg), MSN-PEG/PEI@OMVs (8 μg total OMV protein per mouse), or CpG@MSN-PEG/PEI@OMVs (8.6 μg CpG per mouse) on days 6, 9, 12, and 14. For cytokine depletion studies, neutralizing antibodies against IFNγ (clone XMG1.2, Bio X Cell) and TNFα (clone XT3.11, Bio X Cell) were administered intraperitoneally at a dose of 200 μg in 100 μL of PBS. Treatments began 48 h prior to tumor implantation and continued every 3 days until the study end point. Rat IgG2b (clone LTF-2, Bio X Cell) and rat IgG1 (clone TNP6A7, Bio X Cell) were used as isotype controls. Tumor growth and survival were monitored as previously described.

### 4.20 Cytokine Antibody Arrays

To assess systemic cytokine responses, BALB/c mice were subcutaneously inoculated with 2 × 10^6^ 4T1 cells. Mice received intravenous administration of the indicated formulations on days 13 and 16. On day 23, blood samples were collected via cardiac puncture, and serum was separated by centrifugation at 3000 rpm for 10 min at 4 °C and then stored at −80 °C until analysis. Cytokine profiles were assessed using the Quantibody Mouse Cytokine Antibody Array 640 (RayBiotech, USA; catalog #QAM-CAA-640) following the manufacturer’s protocols. Samples from control, CpG, MSN-PEG/PEI@OMV, and CpG@MSN-PEG/PEI@OMVs treatment groups were analyzed in triplicate. Fluorescent intensities were scanned using the Innoscan 710AL system and quantified with Mapix software.

### 4.21 ELISPOT Assays

The frequency of IFNγ-secreting T cells in mouse splenocytes was determined using the BD Mouse IFNγ ELISPOT Kit (cat. no. 551083) according to the manufacturer’s instructions. Multiscreen 96-well ELISPOT plates (Millipore) were coated overnight at 4 °C with antimouse IFNγ capture antibody. Plates were washed and blocked with RPMI-1640 containing 10% FBS. Splenocytes (2 × 10^5^ cells/well) or negatively selected T cells (EasySep Mouse T Cell Isolation Kit, STEMCELL Technologies, catalog no. 19851) were seeded into each well. Anti-CD3ε and anti-CD28 (Ultra-LEAF, BioLegend) served as positive controls. For tumor antigen-specific reactivation, T cells (4 × 10^5^) were cocultured with 4T1 cells for 24 h at 37 °C. Following incubation, biotinylated detection antibodies and alkaline phosphatase-conjugated streptavidin were added, followed by color development with substrate solution. Spots indicating cytokine-secreting cells were imaged and quantified using an AID vSpot Spectrum Reader (AID, Munich, Germany).

### 4.22 Tumor Rechallenge Models

To evaluate durable antitumor immunity, male C57BL/6 mice were subcutaneously inoculated in the left flank with 5 × 10^6^ MC38 cells in 50 μL of PBS. Mice were intravenously treated with MSN-PEG/PEI@OMVs or CpG@MSN-PEG/PEI@OMVs (in 200 μL of PBS) on days 5, 7, 9, 11, and 13. Concurrently, anti-PD-L1 antibody (clone 10F.9G2, Bio X Cell, 200 μg per injection) was administered intraperitoneally on days 5, 8, 11, and 13. Tumor volume, body weight, and survival were monitored regularly. On day 70, mice with complete tumor remission were rechallenged subcutaneously in the contralateral flank with 1 × 10^5^ MC38 cells. A cohort of three age-matched naive mice was included as a positive control for tumorigenicity. Rechallenge outcomes were used to assess the longevity of protective antitumor memory responses.

### 4.23 RNA-Seq Data Processing

Tumor tissues were harvested from mice that were euthanized with Zoletil and Rompun and immediately snap-frozen in liquid nitrogen. RNA-seq analysis was performed by Welgene Biotechnology Company (Taipei, Taiwan). Total RNA was extracted using TRIzol reagent (Invitrogen, USA), and RNA integrity was assessed using the RNA Integrity Number (RIN) via an Agilent Bioanalyzer 2100 (Agilent Technologies, Santa Clara, CA, USA). RNA samples meeting quality standards were processed by following the Illumina protocol. mRNA libraries were constructed using the Agilent SureSelect XT HS2 mRNA Library Preparation Kit (Agilent, USA), with size selection conducted using AMPure XP Beads. Sequencing was performed with Illumina’s sequencing-by-synthesis platform, generating 150 bp paired-end reads.

Raw sequencing data were processed using the bcl2fastq v2.20 pipeline (Illumina). Adapter trimming and quality filtering were conducted with Trimmomatic (v0.36), retaining high-quality reads. Clean reads were aligned to the mouse reference genome using HISAT2, and gene expression levels were quantified as transcripts per million (TPM). Differential gene expression was analyzed using StringTie (v2.1.4) and DESeq2 (v1.28.1), incorporating bias detection and correction. To identify significant expression changes among the four groups (control, MSN-PEG/PEI, MSN-PEG/PEI@OMVs, and CpG@MSN-PEG/PEI@OMVs), low-abundance genes (<0.3 TPM) were excluded. Genes exhibiting an adjusted *p*-value ≤0.05 and ≥2-fold expression change were considered statistically significant. GO, KEGG, and GSEA were performed using the clusterProfiler R package.^91^ GO enrichment included BP, cellular components, and molecular functions. Enrichment results were filtered based on an adjusted *p*-value <0.05 and a minimum of two unique genes per term. KEGG pathway enrichment followed similar criteria, requiring at least one unique gene per enriched pathway.

### 4.24 ScRNA-Seq Library Construction and Sequencing

TDLNs harvested from 4T1 tumor-bearing mice were processed into single-cell suspensions following the procedures described in the Flow Cytometry section. Single-cell RNA sequencing (scRNA-seq) was conducted by Welgene Biotechnology Company (Taipei, Taiwan). Briefly, the Single Cell 3′ Library and Gel Bead Kit v3.1 (10× Genomics, 1000121) and Chromium Single Cell G Chip Kit (10× Genomics, 1000120) were utilized with the Chromium Single Cell Controller (10× Genomics) to generate single-cell gel beads in emulsion (GEMs) according to the manufacturer’s instructions. Cell suspensions were prepared in PBS containing 0.04% BSA, with approximately 1 × 10^4^ cells loaded per channel, targeting a recovery of ∼6 × 10^3^ cells. Following GEM formation, cell lysis and reverse transcription occurred within individual droplets, with barcoded cDNA synthesized using a Veriti 96-Well Fast Thermal Cycler (Applied Biosystems) under the following thermal profile: 53 °C for 45 min, 85 °C for 5 min, and hold at 4 °C. The resulting cDNA was then amplified, and its quality was assessed using a 4200 Tapestation system (Agilent Technologies). Final libraries were constructed using the same library kit and sequenced on an Illumina NovaSeq X Plus platform, targeting a minimum of 40,000 reads per cell with a 150 bp paired-end sequencing format.

### 4.25 ScRNA-Seq Data Analysis

Raw sequencing data were processed using the Cell Ranger pipeline (10× Genomics). After quality control and removal of empty droplets, high-quality single cells were retained for downstream analysis. Gene expression profiles were denoised and subjected to dimensionality reduction via PCA. Cells were subsequently clustered based on their transcriptomic features using Cell Ranger’s graph-based and K-means clustering algorithms. The graph-based algorithm constructs a network where transcriptionally similar cells are connected via the K-nearest neighbor method and refined through Louvain Modularity Optimization. These clusters were further merged or split according to the differential gene expression results. Cell-type annotation was performed using the SingleR package, which maps each cell’s expression profile to reference data sets from the ImmGen consortium for mouse samples. To identify differential expression across the same cell types between groups, Seurat (v5.0.1) was employed to calculate the *p*-values using normalized expression matrices. Functional enrichment analyses—including GO, KEGG, and GSEA—were performed using the clusterProfiler R package following the analytical pipeline described in the RNA-Seq Data Processing section.

### 4.26 Statistical Analysis

All statistical analyses were performed using GraphPad Prism (v10.4.1). Data are presented as mean ± standard deviation. Comparisons between groups were analyzed using one-way or two-way ANOVA with Tukey’s post hoc test, unless otherwise specified. Statistical significance was denoted as follows: *p < 0.05, **p < 0.01, ***p < 0.001, and ****p < 0.0001.

## Supplementary Material



## Notes

Experimental methods for NP synthesis, OMV isolation, CpG encapsulation, and flow cytometry; physicochemical characterization data; procedures for biodistribution, safety, and therapeutic studies; light sheet and expansion microscopy protocols; immunological assays including cytokine profiling, ELISpot, and IHC; and RNA-seq and scRNA-seq analysis workflows and gene expression data (PDF)

## ∇

Deceased (2023). We dedicate this work to the memory of Kurt Yun Mou, whose guidance and contributions were integral to this research.
