# Supplementary material for: The Bacterial Outer Membrane Vesicle-Cloaked Immunostimulatory Nanoplatform Reinvigorates T Cell Function and Reprograms Tumor Immunity
Source: ACS Nano. 2025 May 20;19(21):19866–89. doi: 10.1021/acsnano.5c02541 (PMC12139043; doi:10.1021/acsnano.5c02541)
Supplement: Supplementary file 1 [file nn5c02541_si_001.pdf]

Supporting information

The Bacterial Outer Membrane Vesicle-Cloaked Immunostimulatory Nanoplatform Reinvigorates T Cell Function and Reprograms Tumor Immunity

Yu-Han Lin[1,2,3], Chia-Wei Chen[2], Mei-Yi Chen[3,†], Li Xu[2,†], Xuejiao Tian[4,5], Siu-Hung Cheung[2], Yen-Ling Wu[2], Natnaree Siriwon[6], Si-Han Wu[2,7]*, Kurt Yun Mou[1,3‡]

[1]*Taiwan International Graduate Program in Molecular Medicine, National Yang Ming Chiao Tung University and Academia Sinica, Taipei, 11529, Taiwan*

[2]*Graduate Institute of Nanomedicine and Medical Engineering, Taipei Medical University, Taipei, 11031, Taiwan*

[3]*Institute of Biomedical Sciences, Academia Sinica, Taipei, 11529, Taiwan*

[4]*Research Center for Applied Sciences and Nano Science and Technology Program, Taiwan International Graduate Program, Academia Sinica, Taipei 11529, Taiwan*

[5]*Department of Engineering and System Science, National Tsing Hua University, Hsinchu 30013, Taiwan*

[6]*Chakri Naruebodindra Medical Institute, Faculty of Medicine Ramathibodi Hospital, Mahidol University, Samutprakarn 10540, Thailand*

[7]*International Ph.D. Program in Biomedical Engineering, Taipei Medical University, Taipei 11031, Taiwan*

[†]*Both authors contributed equally to this work*

[‡] *Deceased (2023). We dedicate this work to the memory of Kurt Yun Mou, whose guidance and contributions were integral to this research.*

Corresponding author's Email: smilehanwu@tmu.edu.tw

Supporting Information 1

(Figures S1-1 to S1-25 and Table S1)

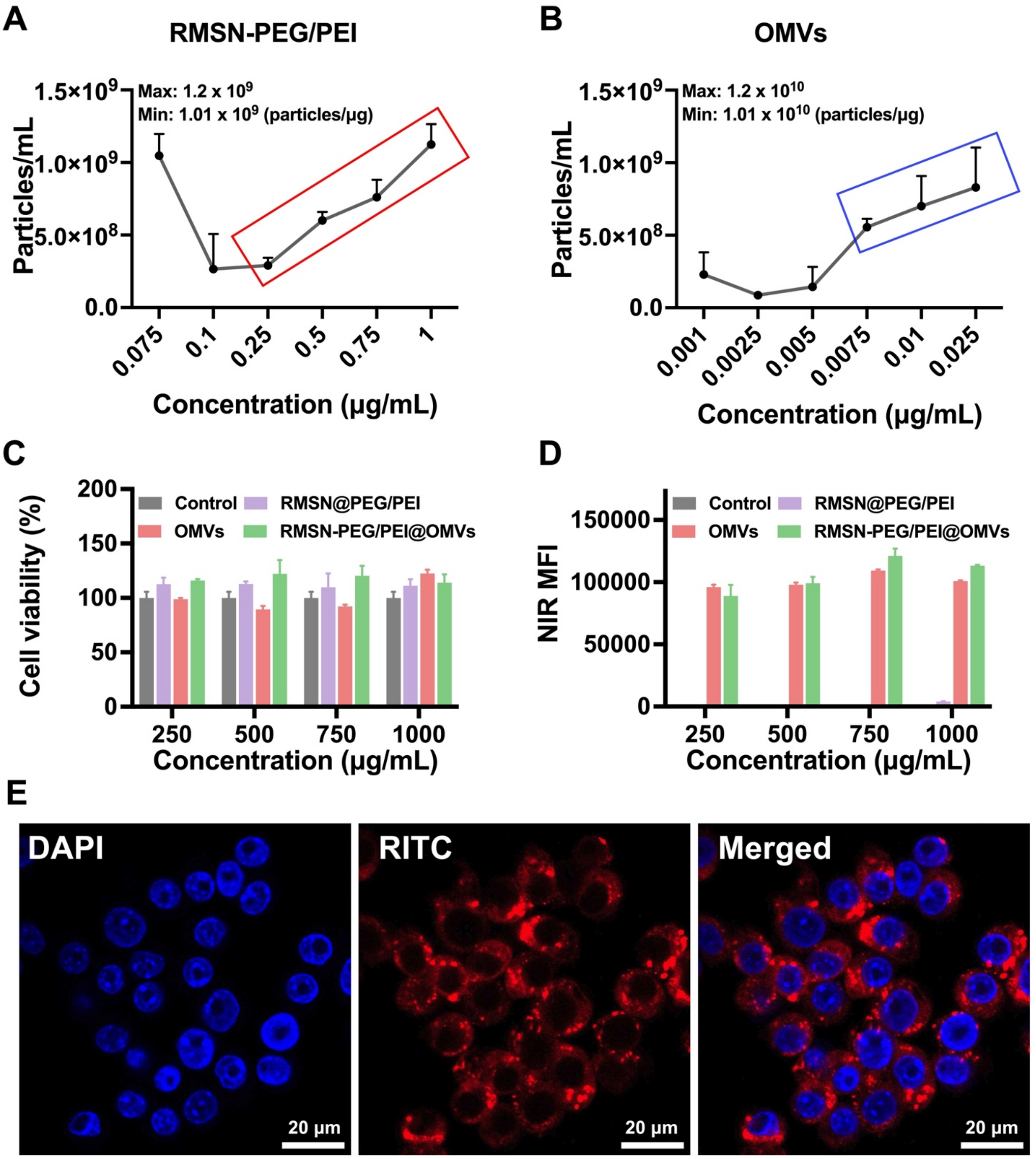

Figure S1-1. Physicochemical characteristics of nanoparticles and *in vitro* biocompatibility test after nanoparticle exposure. Nanoparticle tracking analysis of 25 nm **(A)** RMSN@PEG/PEI and **(B)** RMSN-PEG/PEI@OMVs. Linear optimal concentrations indicated by red and blue frames. Quantitative examination in RAW 264.7 cells incubated 24h with various concentrations of

nanoparticles on **(C)** cell viability by CCK8 assay and **(D)** cellular internalization with near-infrared (NIR) fluorescence dye by flow cytometry. **(E)** Confocal microscopy image of RAW 264.7 cells incubated with RITC-labeled RMSN-PEG/PEI@OMVs for 24 h. RITC (red) signals surrounding DAPI-stained nuclei (blue) confirm intracellular localization.

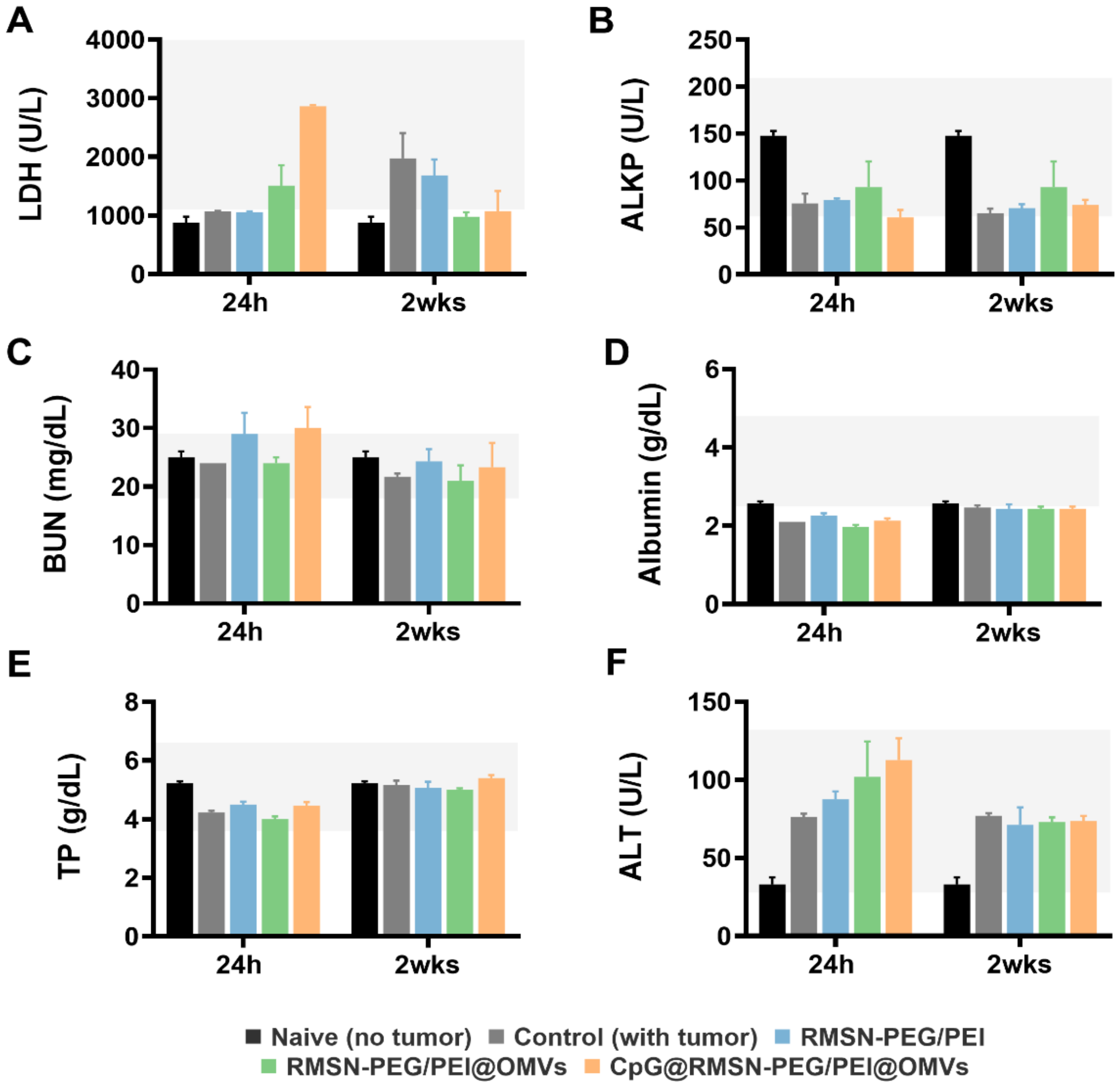

Figure S1-2. *In vivo* biological safety evaluation after various nanoparticle treatments by intravenous injection on BALB/c mice. (A-F) Clinical biochemistry profile after once and twice administrations. Intervals within grey blocks served as normal reference value for healthy BALB/c mice.

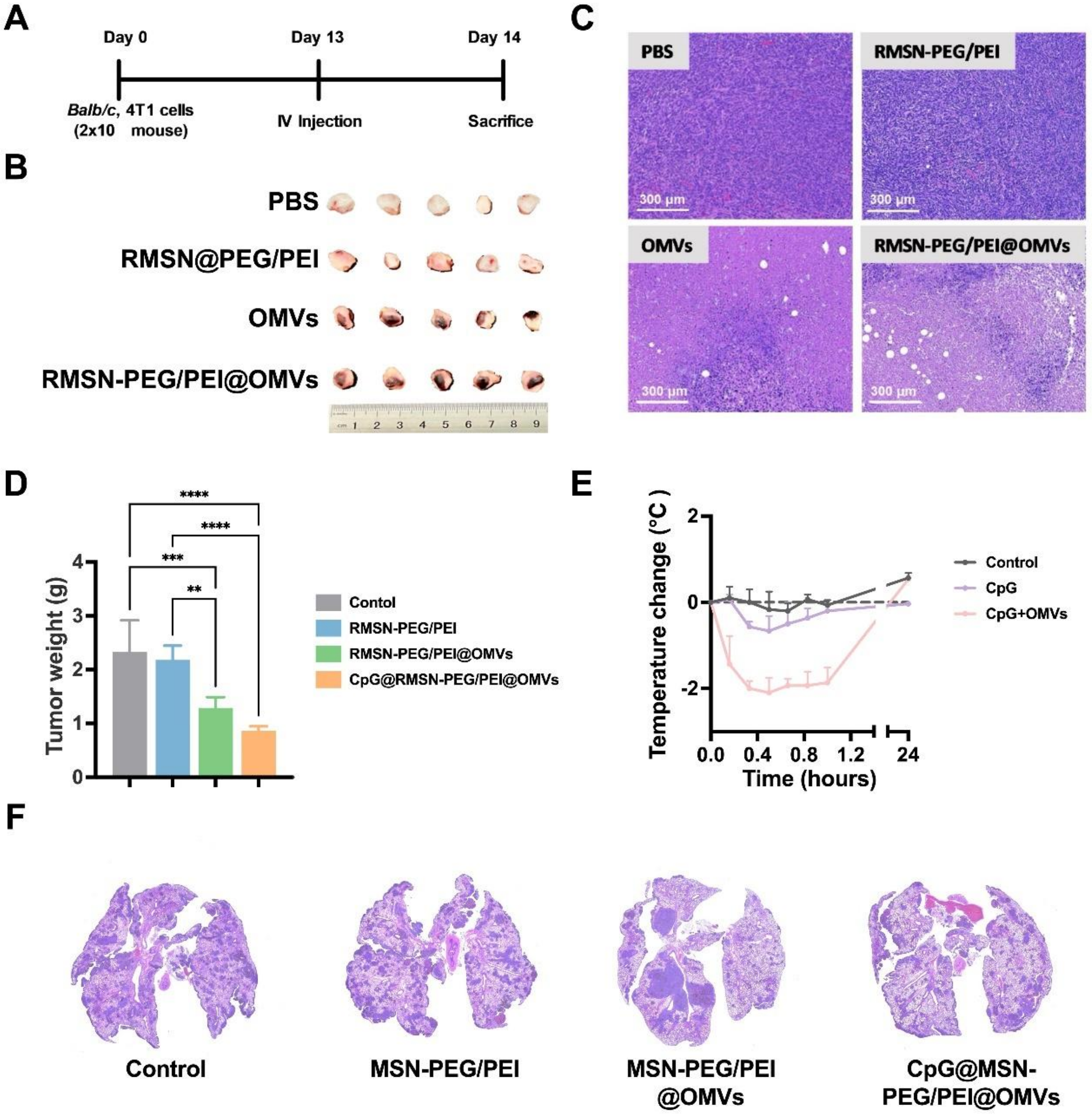

Figure S1-3. ***In vivo*** **biological safety evaluation after various nanoparticle treatments by intravenous injection on BALB/c mice.** *In vivo* necrosis analysis presented by **(A)** treatment schedule, **(B)** tumors assessment and **(C)** H&E staining. **(D)** Tumor weight measurement from tumor isolated post 30 days after two doses of various treatments. **(E)** Rectal temperature monitored from 1 to 24h post-injection. **(F)** H&E stained whole sections of lung from C57BL/6 mice intravenously administrated nanoparticles on day 4 and day 8 after injection with B16F10 melanoma cells (n = 3 per group) through tail vein.

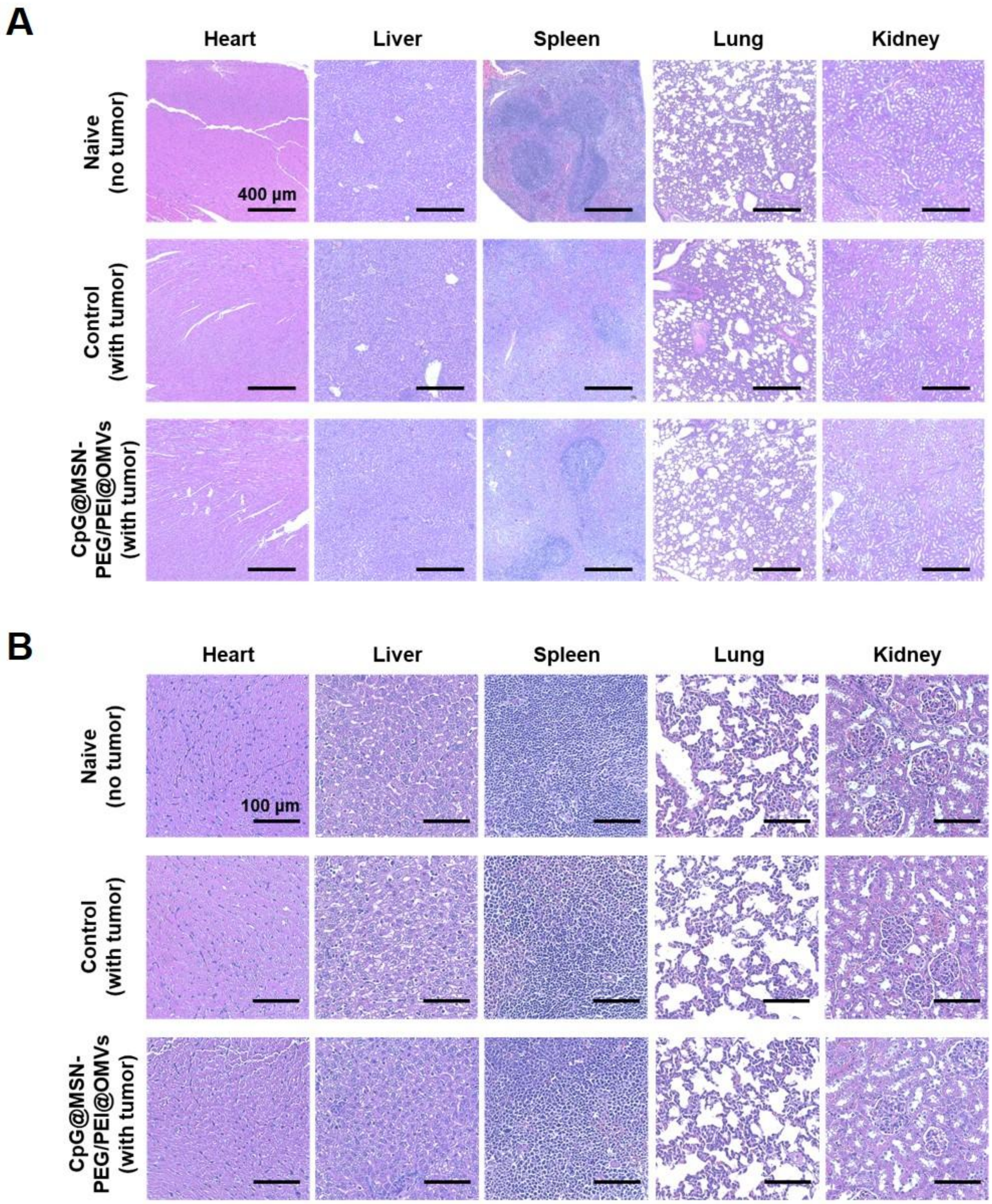

Figure S1-4. ***In vivo*** **biological safety evaluation after various nanoparticle treatments by intravenous injection on BALB/c mice. (A-B)** H&E staining in different organs collected post 7 days after two injections. Magnification: 10X and 40X, Scale bars: 400 μm and 100 μm, respectively.

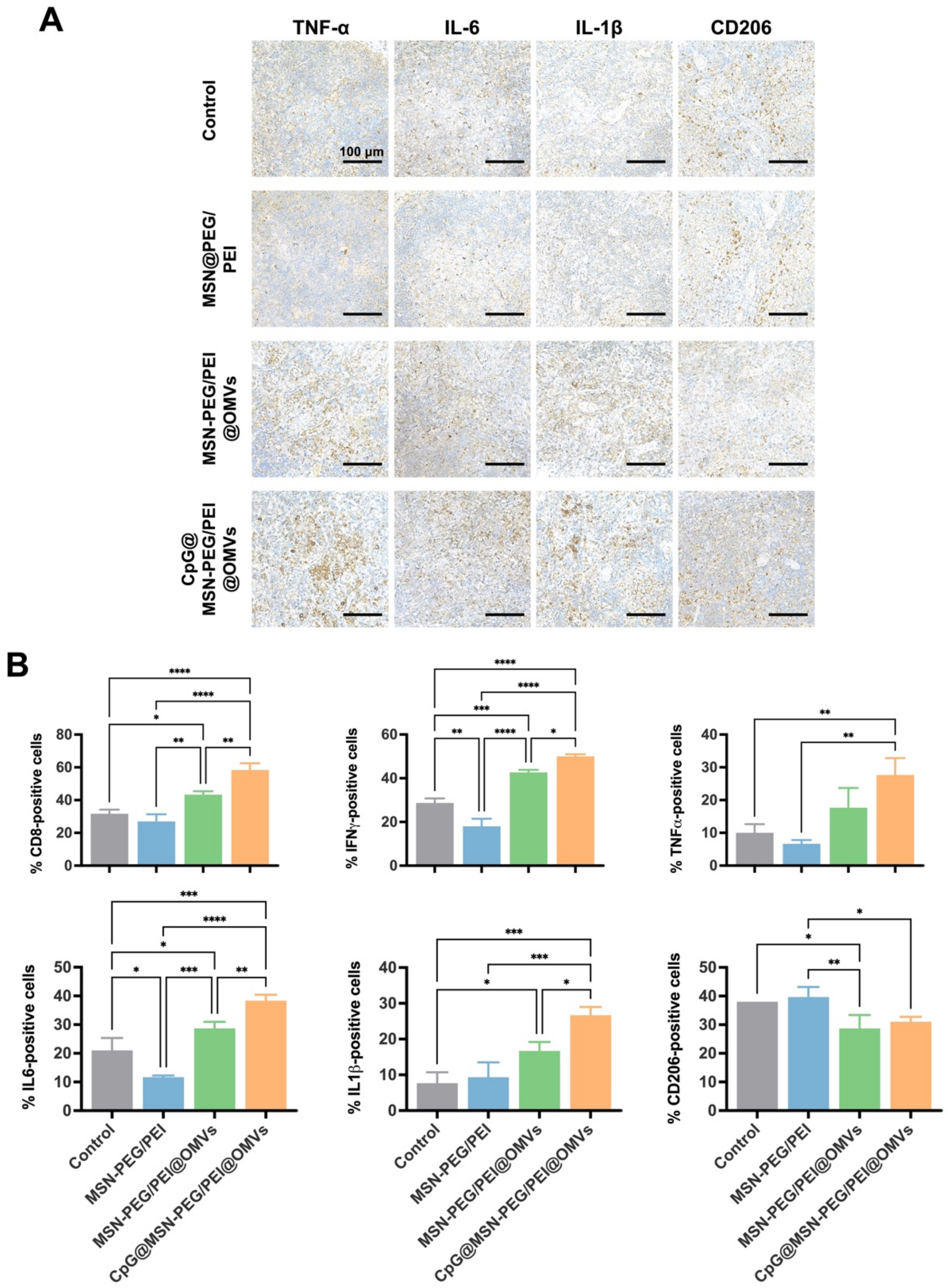

Figure S1-5. Histological investigation of cytokine release in TDLNs after OMV nanohybrid treatment (A) Immunohistochemistry (top panel) (scale bar: 100 μm) and **(B)** quantitative analysis (bottom panel) of CD8, IFN-γ, TNF-α, IL-6, IL-1β and CD206 in TDLNs collected post 23 days after second injections from BALB/c mice intravenously treated with the different nanoparticle groups (n = 3 per group).

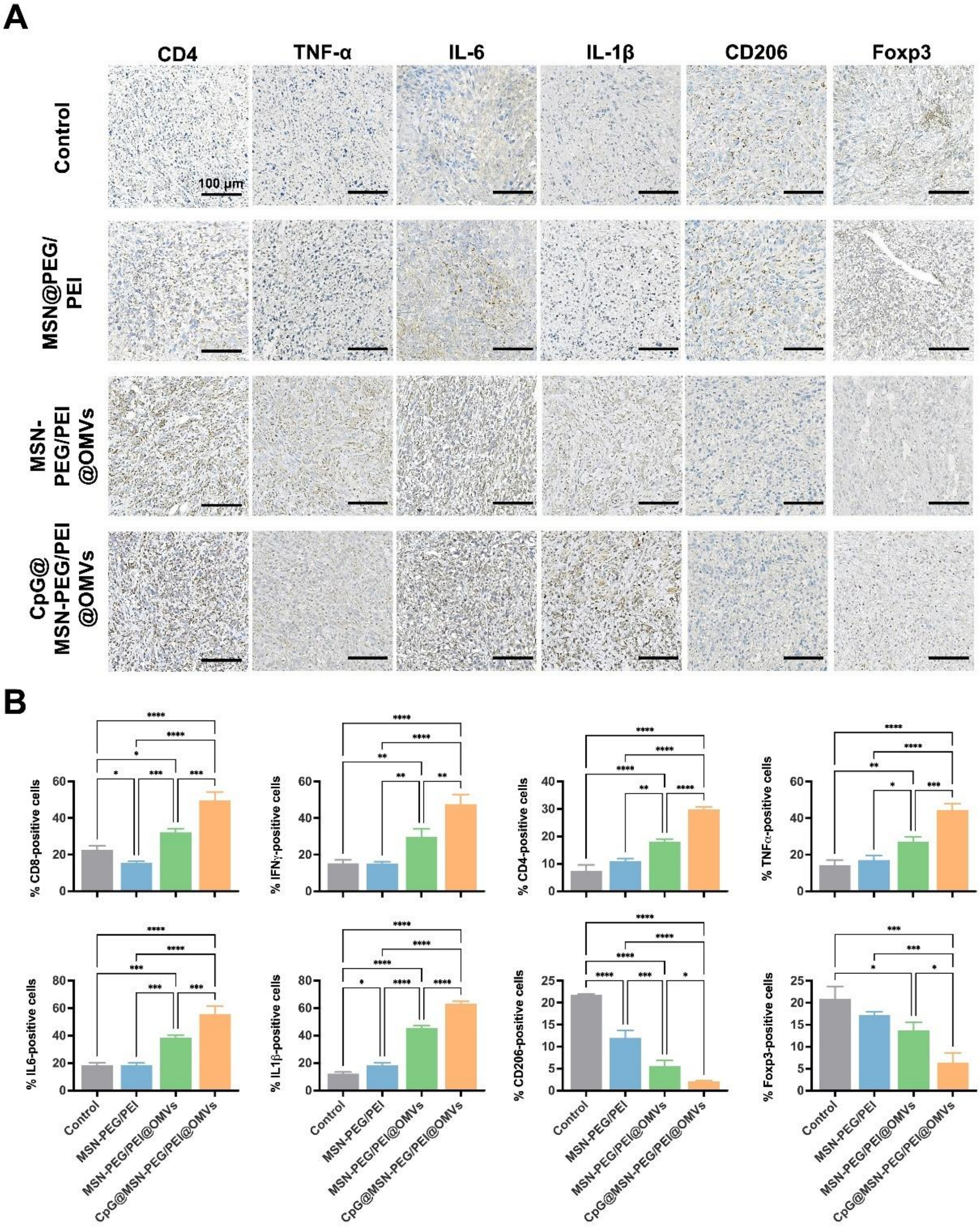

Figure S1-6. Histological investigation of cytokine release in tumor after OMV nanohybrid treatment (A) Immunohistochemistry (top panel) (scale bar: 100 μm) and **(B)** quantitative analysis (bottom panel) of CD8, IFN-γ, CD4, TNF-α, IL-6, IL-1β, CD206 and Foxp3 in tumors collected post 23 days after second injections from BALB/c mice intravenously treated with the different nanoparticle groups (n = 3 per group).

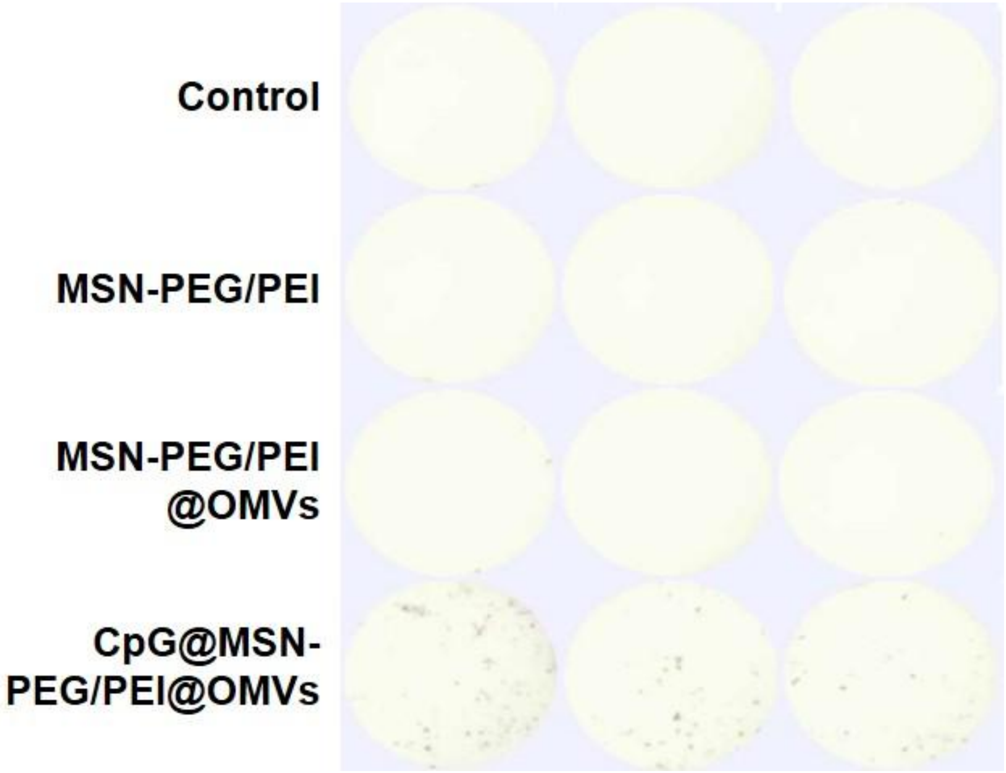

Figure S1-7. Generation of IFNγ-secreting T cell response after OMV nanohybrid treatment. ELISpot responses from IFN-γ secreting T lymphocytes in spleens collected post 23 days after second injections from BALB/c mice intravenously treated with the different nanoparticle groups (n = 3 per group).

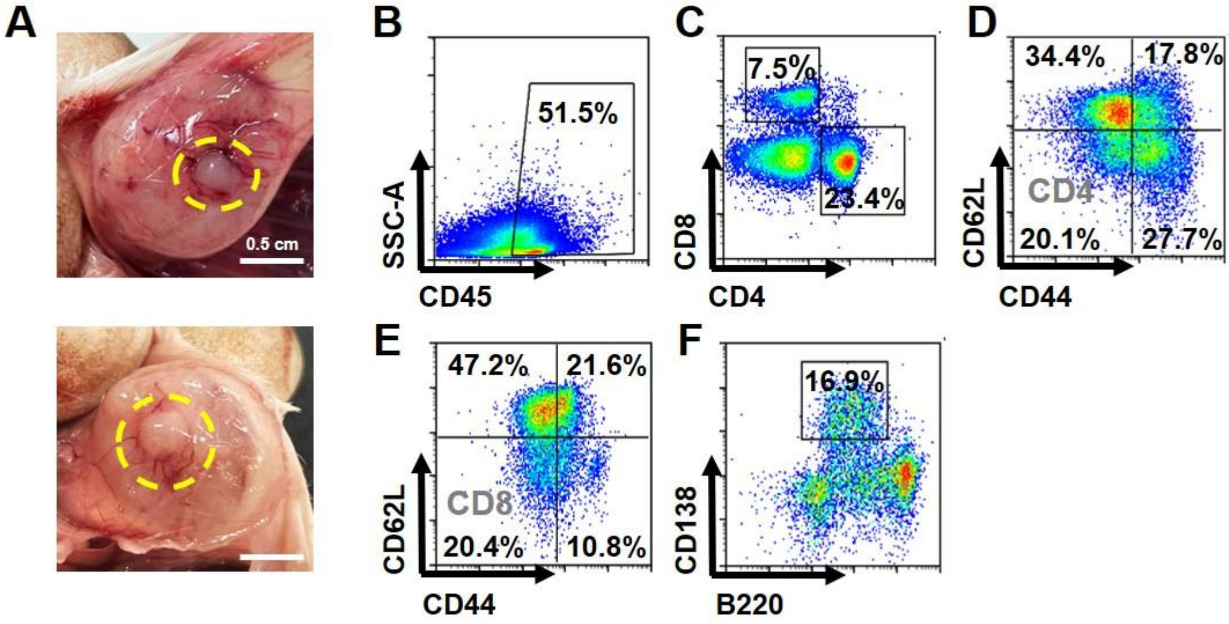

Figure S1-8. Induction of lymph node-like structures (LNS) in 4T1 tumor after CpG@MSN-PEG/PEI@OMV treatment. (A) LNS defined by yellow dashed line in CpG@MSN-

PEG/PEI@OMVs treatment group (scale bar: 0.5 cm). Representative flow cytometric charts of expression on **(B)** leukocytes ($CD45^+$), **(C)** T cells ($CD4^+$ or $CD8^+$) subsets in LNS. T cells ($CD4^+$ or $CD8^+$) further subdivided into **(D)** effector memory T cells ($CD44^+CD62L^-$), **(E)** central memory T cells ($CD44^-CD62L^+$) and **(F)** plasma cells ($CD138^+B220^-$) subpopulations.

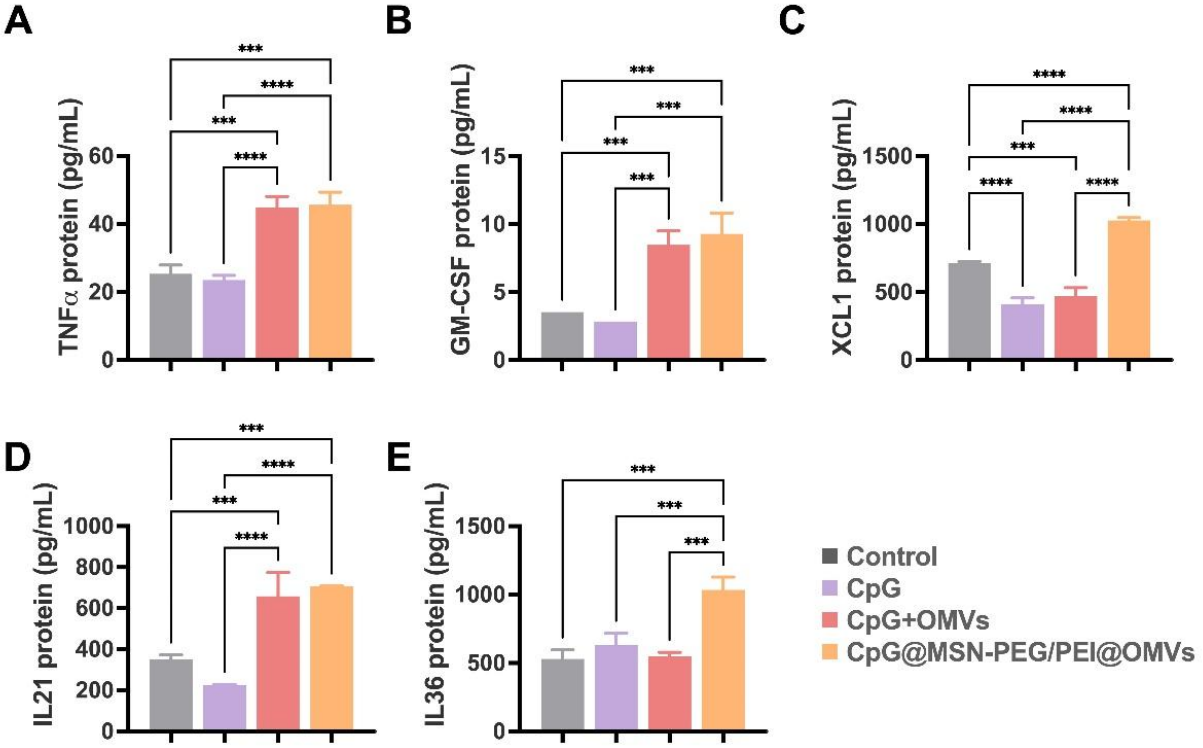

Figure S1-9. Quantibody multiplex ELISA array analysis of cytokine release in blood after OMV nanohybrid treatment. (A) TNF-α, **(B)** GM-CSF, **(C)** XCL1, **(D)** IL-21 and **(E)** IL-36 in blood collected post 23 days after second injections from BALB/c mice intravenously treated with the different nanoparticle groups (n = 3 per group).

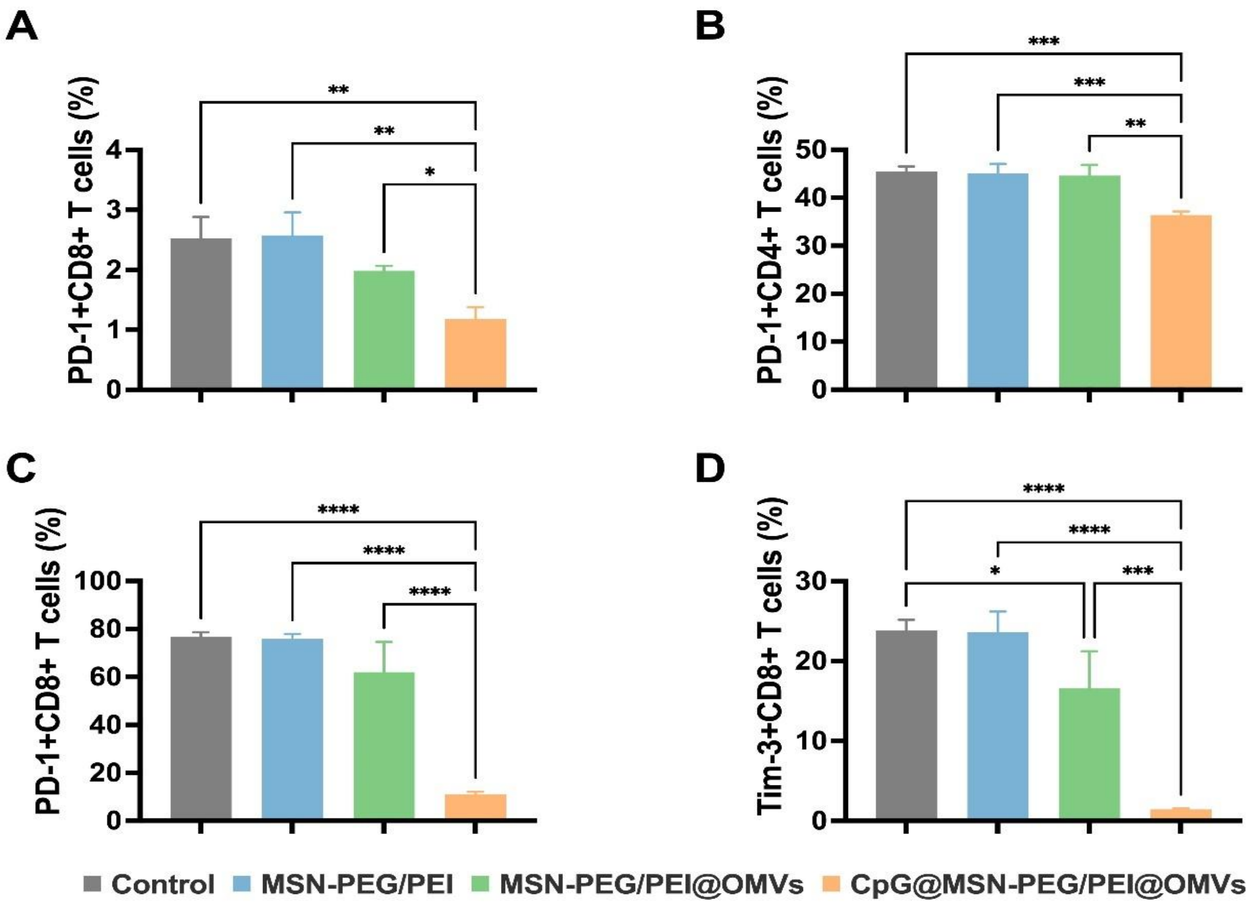

Figure S1-10. Flow cytometric quantification of surface markers PD-1 or Tim-3 on CD8 and CD4 T cells in TDLNs or tumor after OMV nanohybrid treatment. (A) PD-1^{+}CD8^{+} T cells in TDLNs **(B)** PD-1^{+}CD4^{+} T cells, **(C)** PD-1^{+}CD8^{+} T cells and **(D)** Tim-3^{+}CD8^{+} T cells in tumor isolated from 4T1-bearing BALB/c mice (n = 3 per group).

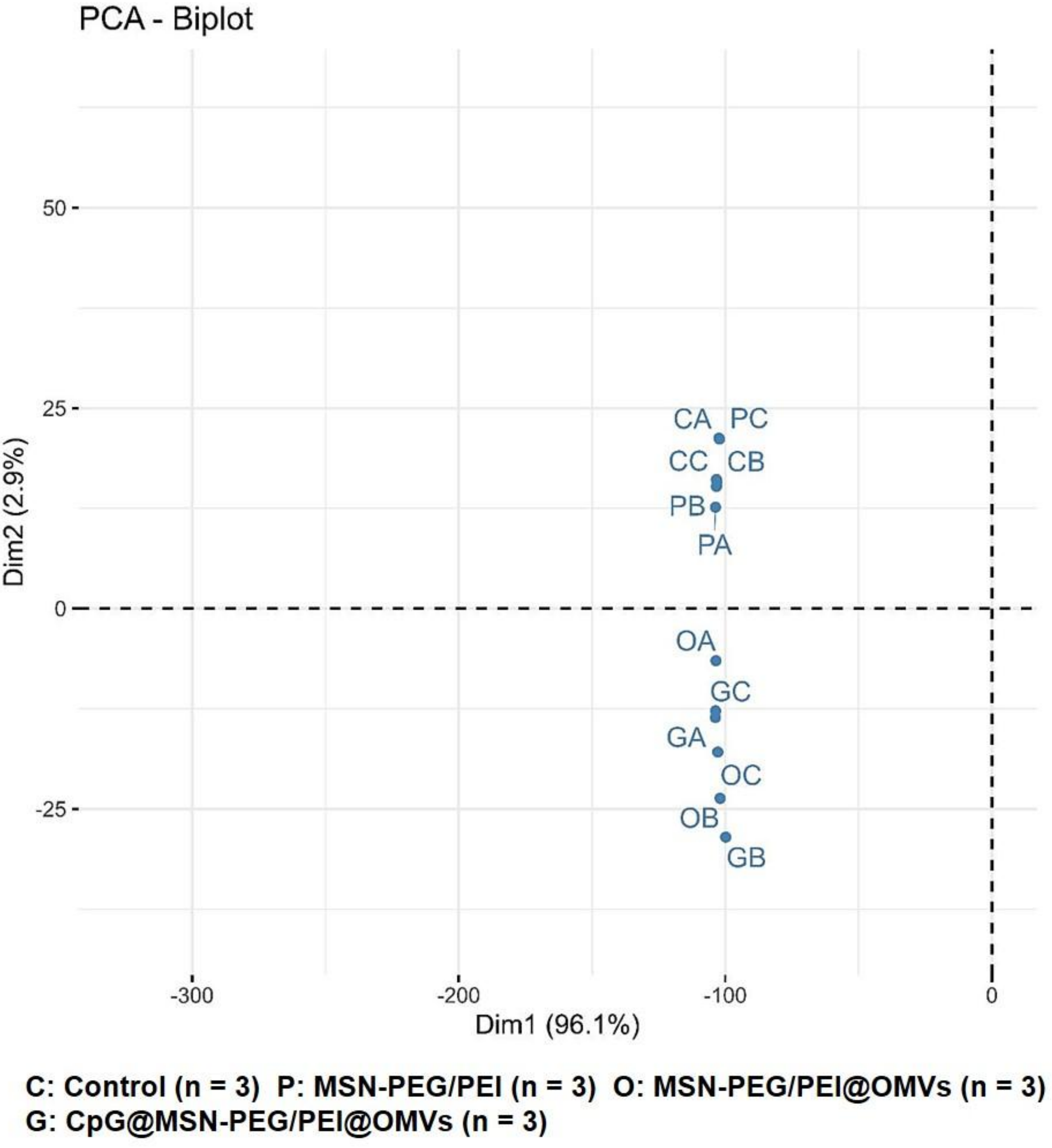

Figure S1-11. Principal component analysis (PCA) map of 4T1 tumor after OMV nanohybrid treatment.

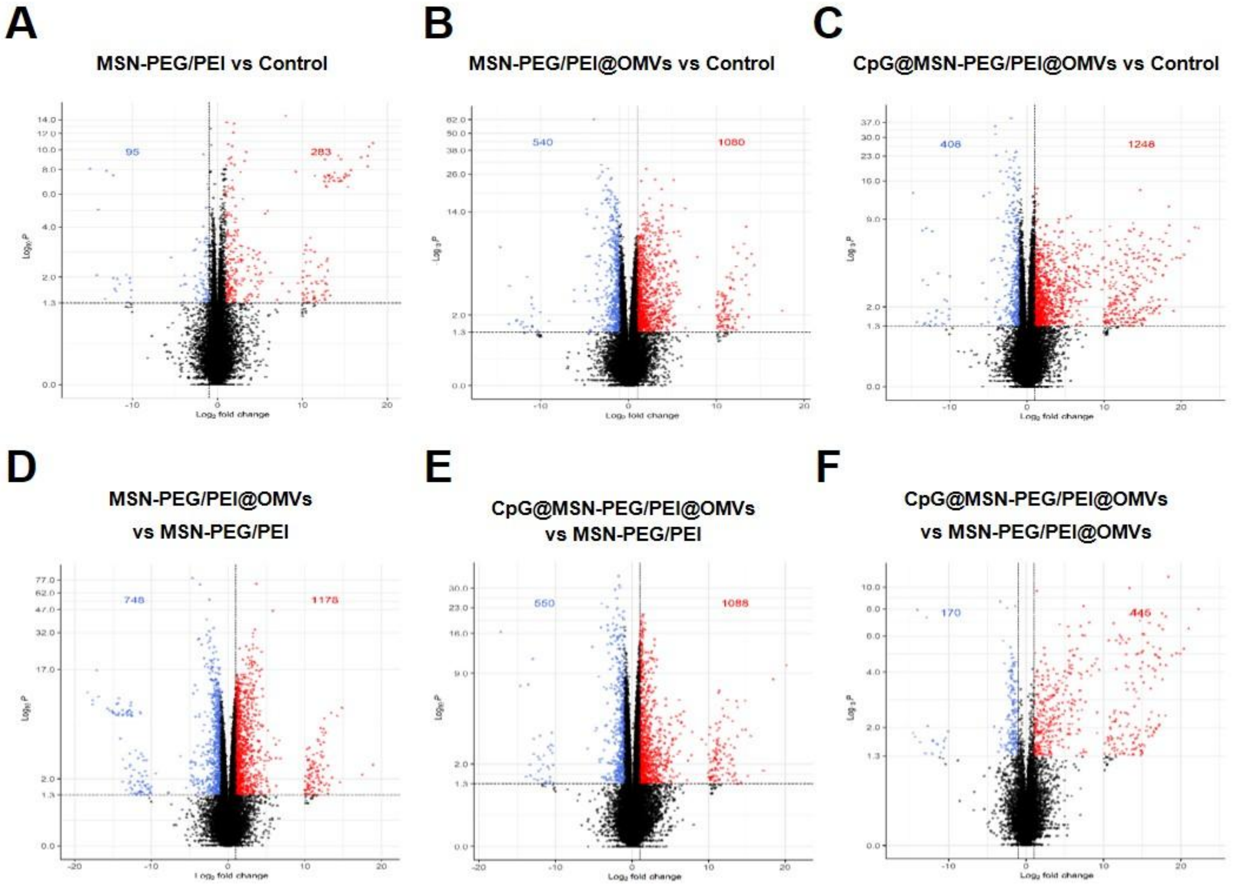

Figure S1-12. Volcano plot comparative expression analysis of tumors after OMV nanohybrid treatment. Red dots and blue dots representing significant changes in gene expression.

A

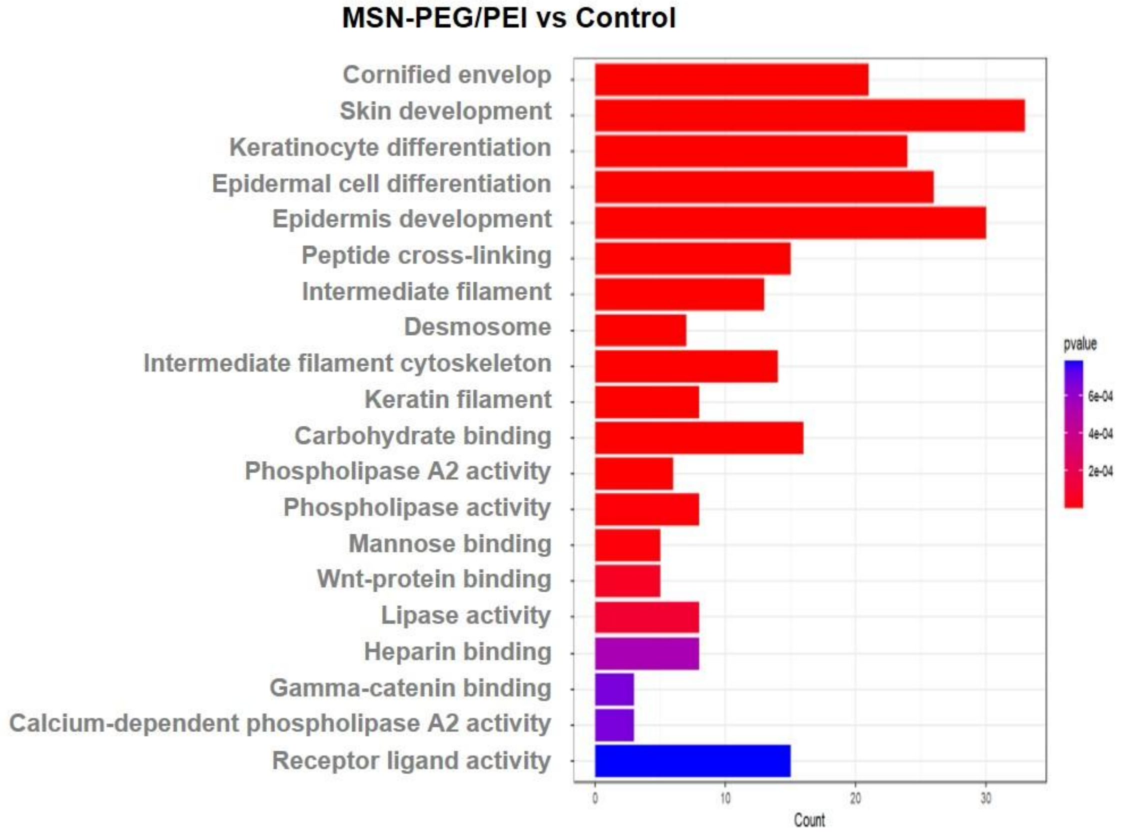
MSN-PEG/PEI vs Control
Cornified envelop
Skin development
Keratinocyte differentiation
Epidermal cell differentiation
Epidermis development
Peptide cross-linking
Intermediate filament
Desmosome
Intermediate filament cytoskeleton
Keratin filament
Carbohydrate binding
Phospholipase A2 activity
Phospholipase activity
Mannose binding
Wnt-protein binding
Lipase activity
Heparin binding
Gamma-catenin binding
Calcium-dependent phospholipase A2 activity
Receptor ligand activity
pvalue
6e-04
4e-04
2e-04
0
10
20
30
Count

B

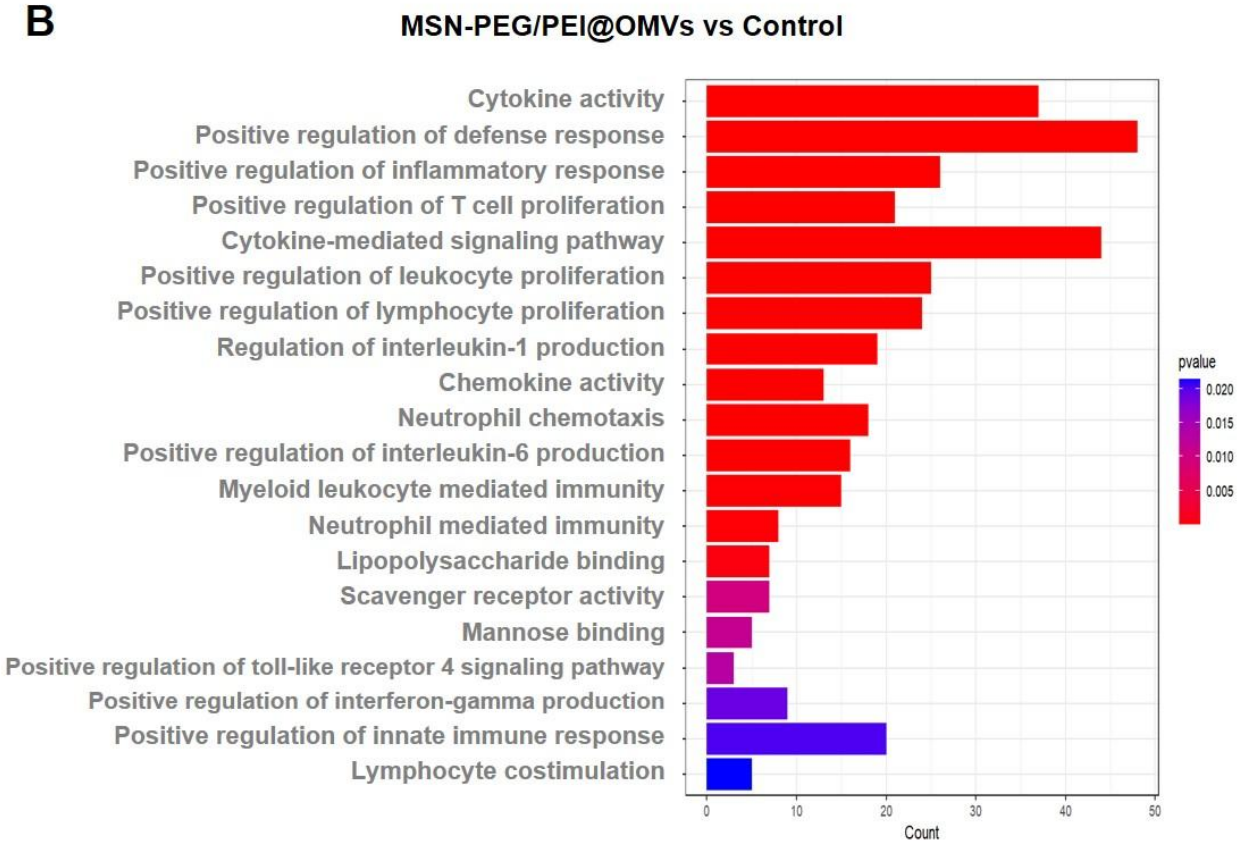
MSN-PEG/PEI@OMVs vs Control
Cytokine activity
Positive regulation of defense response
Positive regulation of inflammatory response
Positive regulation of T cell proliferation
Cytokine-mediated signaling pathway
Positive regulation of leukocyte proliferation
Positive regulation of lymphocyte proliferation
Regulation of interleukin-1 production
Chemokine activity
Neutrophil chemotaxis
Positive regulation of interleukin-6 production
Myeloid leukocyte mediated immunity
Neutrophil mediated immunity
Lipopolysaccharide binding
Scavenger receptor activity
Mannose binding
Positive regulation of toll-like receptor 4 signaling pathway
Positive regulation of interferon-gamma production
Positive regulation of innate immune response
Lymphocyte costimulation
pvalue
0.020
0.015
0.010
0.005
0
10
20
30
40
50
Count

C

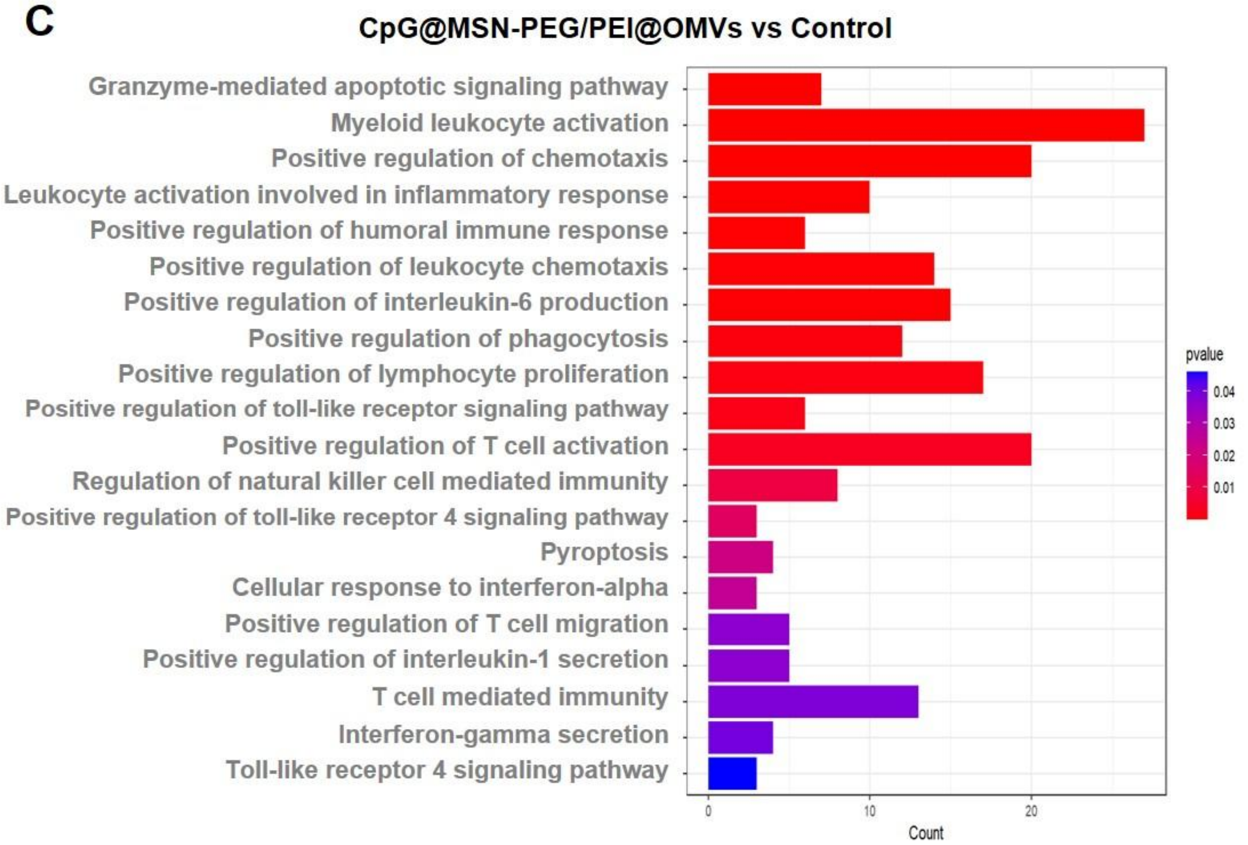
CpG@MSN-PEG/PEI@OMVs vs Control
Granzyme-mediated apoptotic signaling pathway
Myeloid leukocyte activation
Positive regulation of chemotaxis
Leukocyte activation involved in inflammatory response
Positive regulation of humoral immune response
Positive regulation of leukocyte chemotaxis
Positive regulation of interleukin-6 production
Positive regulation of phagocytosis
Positive regulation of lymphocyte proliferation
Positive regulation of toll-like receptor signaling pathway
Positive regulation of T cell activation
Regulation of natural killer cell mediated immunity
Positive regulation of toll-like receptor 4 signaling pathway
Pyroptosis
Cellular response to interferon-alpha
Positive regulation of T cell migration
Positive regulation of interleukin-1 secretion
T cell mediated immunity
Interferon-gamma secretion
Toll-like receptor 4 signaling pathway
0
10
20
Count
pvalue
0.04
0.03
0.02
0.01

D

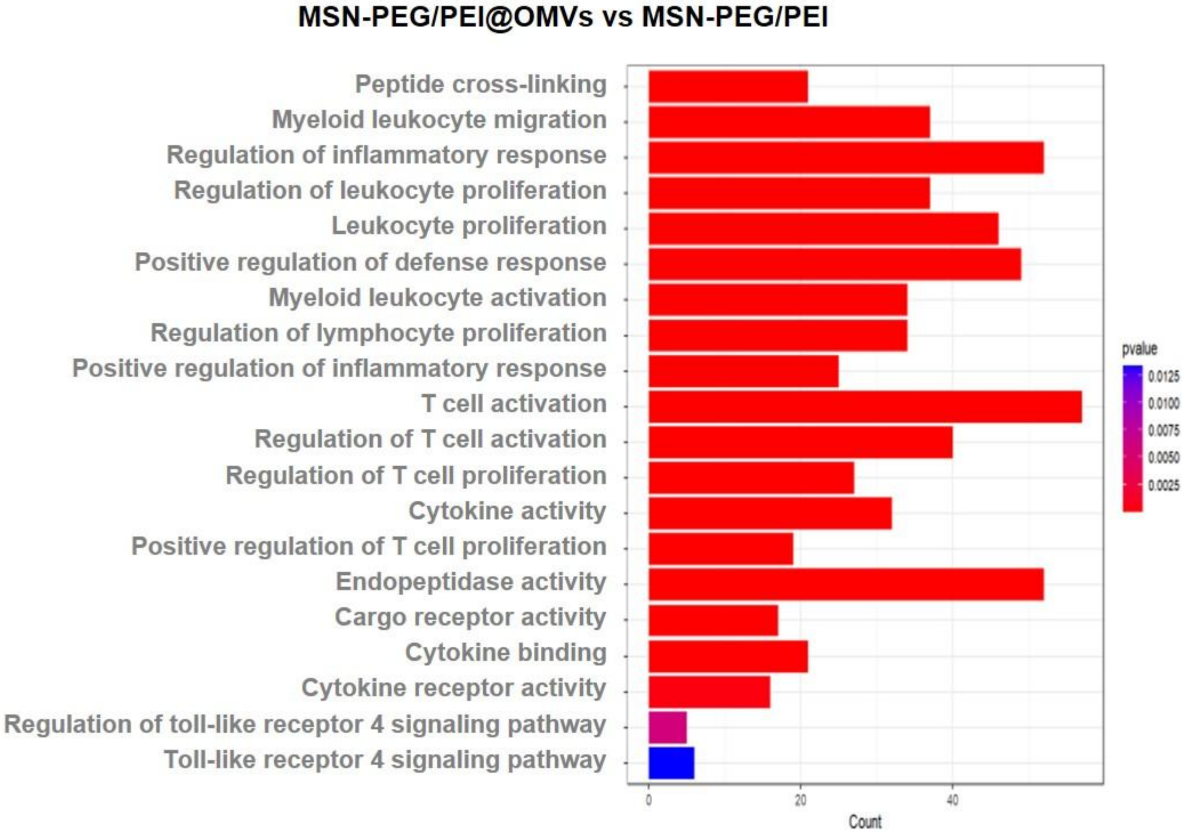
MSN-PEG/PEI@OMVs vs MSN-PEG/PEI
Peptide cross-linking
Myeloid leukocyte migration
Regulation of inflammatory response
Regulation of leukocyte proliferation
Leukocyte proliferation
Positive regulation of defense response
Myeloid leukocyte activation
Regulation of lymphocyte proliferation
Positive regulation of inflammatory response
T cell activation
Regulation of T cell activation
Regulation of T cell proliferation
Cytokine activity
Positive regulation of T cell proliferation
Endopeptidase activity
Cargo receptor activity
Cytokine binding
Cytokine receptor activity
Regulation of toll-like receptor 4 signaling pathway
Toll-like receptor 4 signaling pathway
0
20
40
Count
pvalue
0.0125
0.0100
0.0075
0.0050
0.0025

E

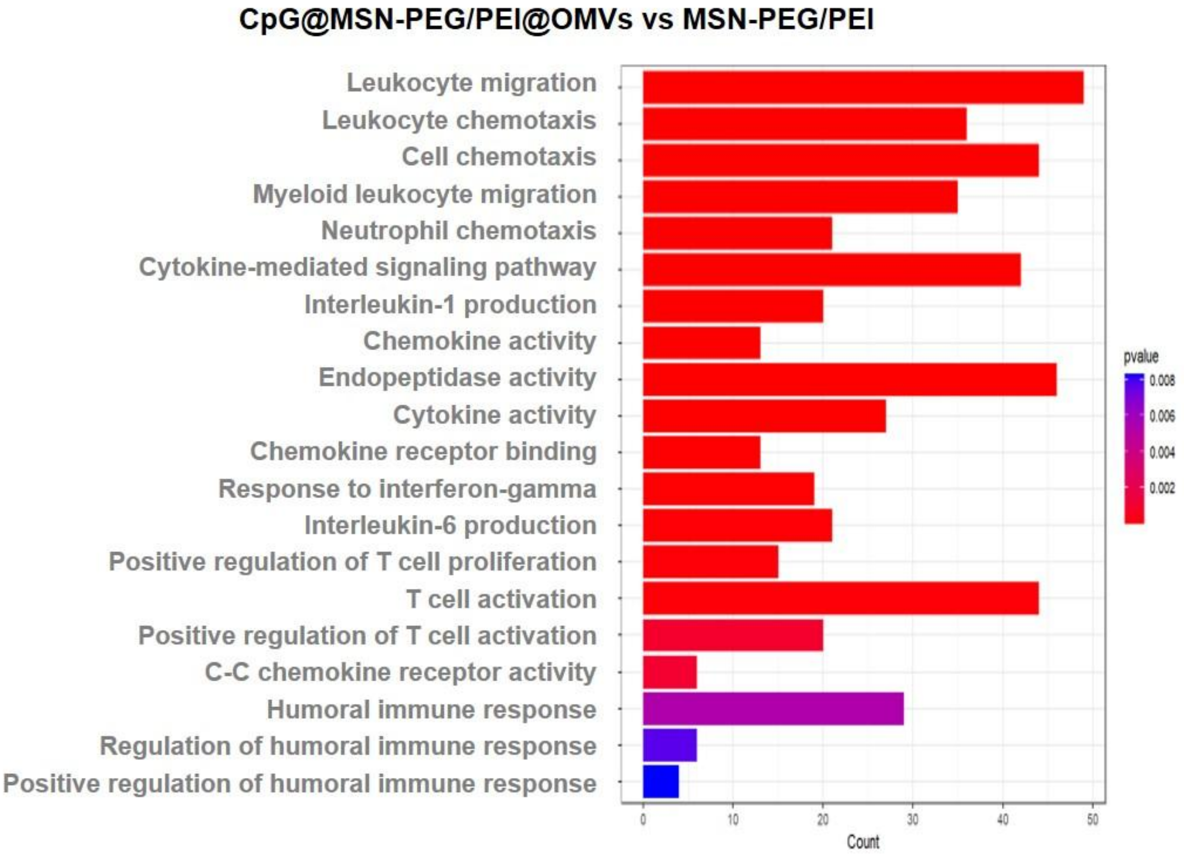

Figure S1-13. Identification of significant GO process enrichment terms in TME after OMV nanohybrid treatment.

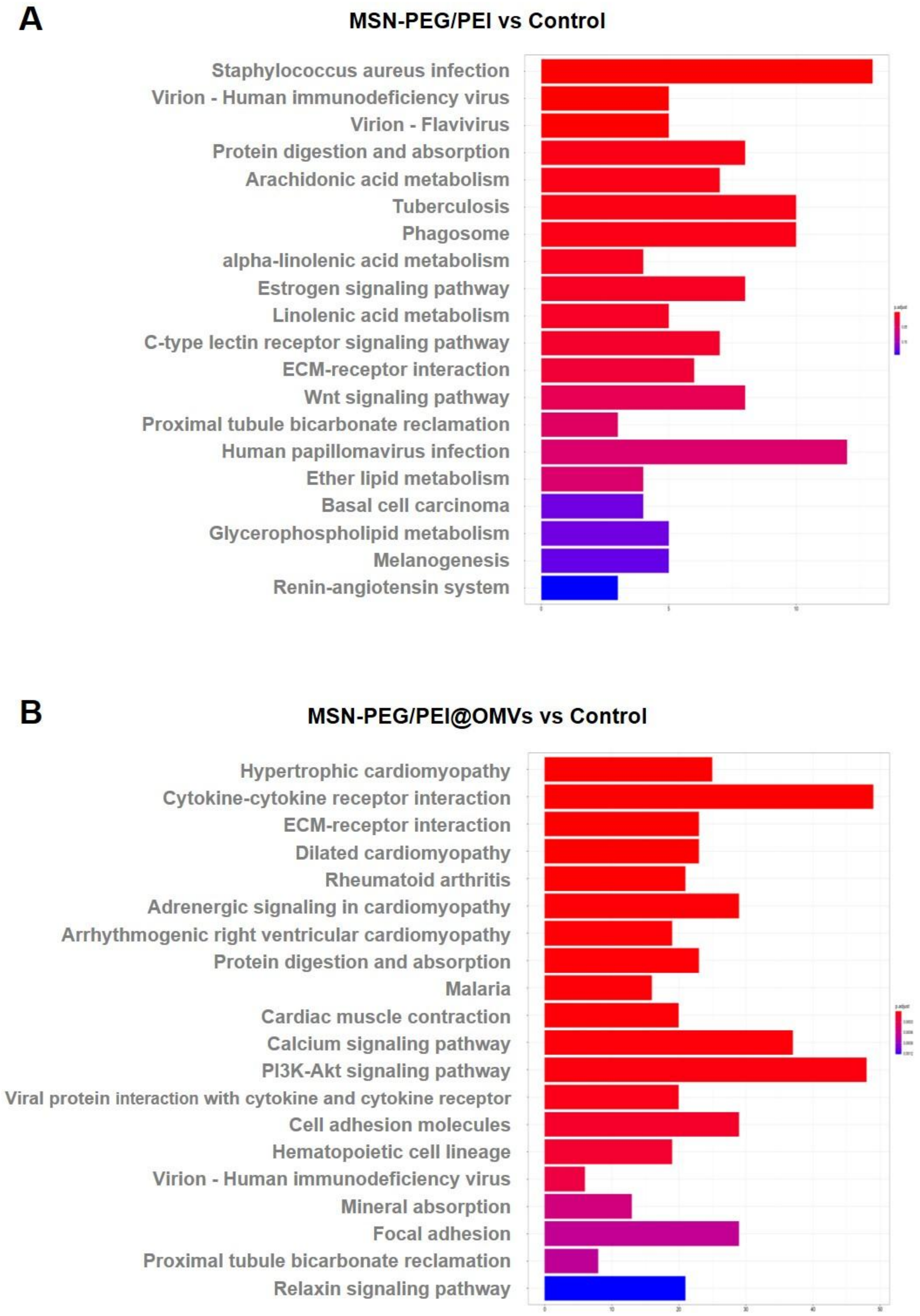
A
MSN-PEG/PEI vs Control
Staphylococcus aureus infection
Virion - Human immunodeficiency virus
Virion - Flavivirus
Protein digestion and absorption
Arachidonic acid metabolism
Tuberculosis
Phagosome
alpha-linolenic acid metabolism
Estrogen signaling pathway
Linolenic acid metabolism
C-type lectin receptor signaling pathway
ECM-receptor interaction
Wnt signaling pathway
Proximal tubule bicarbonate reclamation
Human papillomavirus infection
Ether lipid metabolism
Basal cell carcinoma
Glycerophospholipid metabolism
Melanogenesis
Renin-angiotensin system
B
MSN-PEG/PEI@OMVs vs Control
Hypertrophic cardiomyopathy
Cytokine-cytokine receptor interaction
ECM-receptor interaction
Dilated cardiomyopathy
Rheumatoid arthritis
Adrenergic signaling in cardiomyopathy
Arrhythmogenic right ventricular cardiomyopathy
Protein digestion and absorption
Malaria
Cardiac muscle contraction
Calcium signaling pathway
PI3K-Akt signaling pathway
Viral protein interaction with cytokine and cytokine receptor
Cell adhesion molecules
Hematopoietic cell lineage
Virion - Human immunodeficiency virus
Mineral absorption
Focal adhesion
Proximal tubule bicarbonate reclamation
Relaxin signaling pathway

C

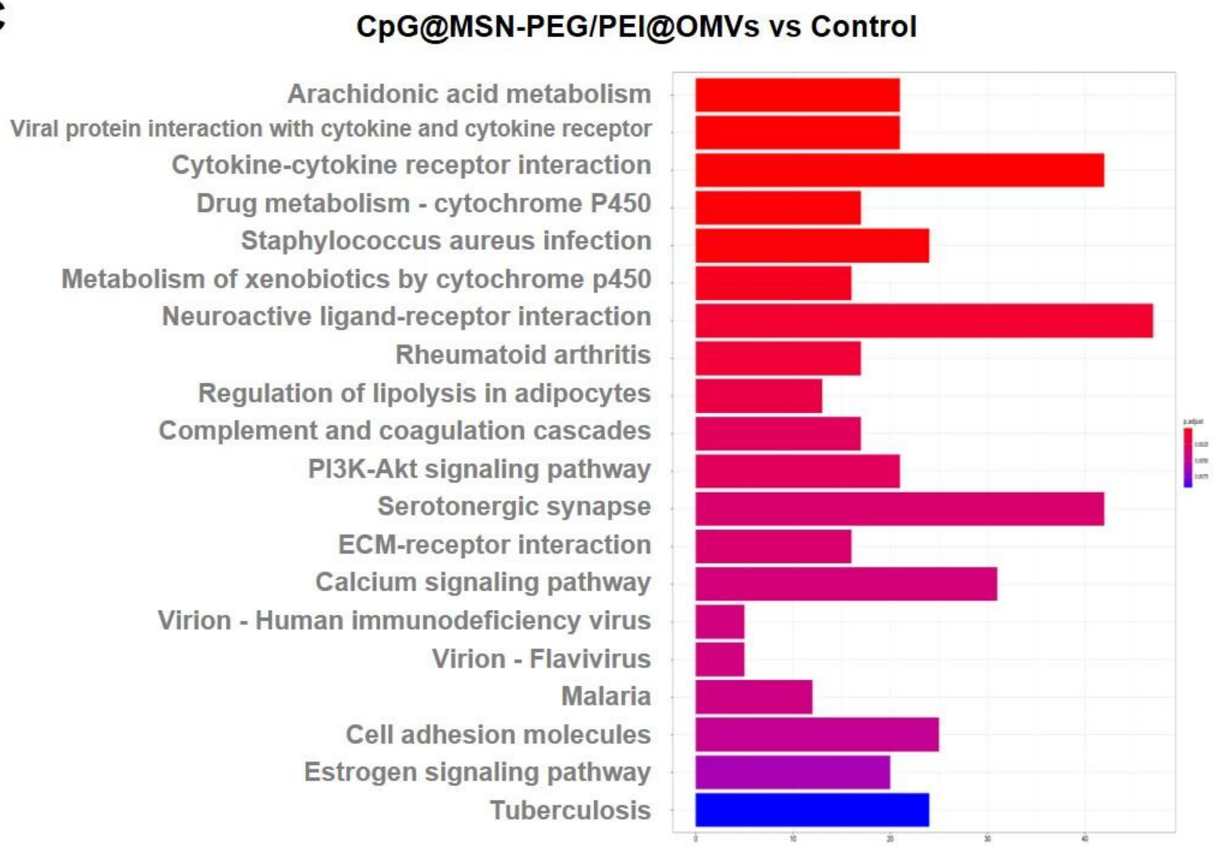
CpG@MSN-PEG/PEI@OMVs vs Control
Arachidonic acid metabolism
Viral protein interaction with cytokine and cytokine receptor
Cytokine-cytokine receptor interaction
Drug metabolism - cytochrome P450
Staphylococcus aureus infection
Metabolism of xenobiotics by cytochrome p450
Neuroactive ligand-receptor interaction
Rheumatoid arthritis
Regulation of lipolysis in adipocytes
Complement and coagulation cascades
PI3K-Akt signaling pathway
Serotonergic synapse
ECM-receptor interaction
Calcium signaling pathway
Virion - Human immunodeficiency virus
Virion - Flavivirus
Malaria
Cell adhesion molecules
Estrogen signaling pathway
Tuberculosis

D

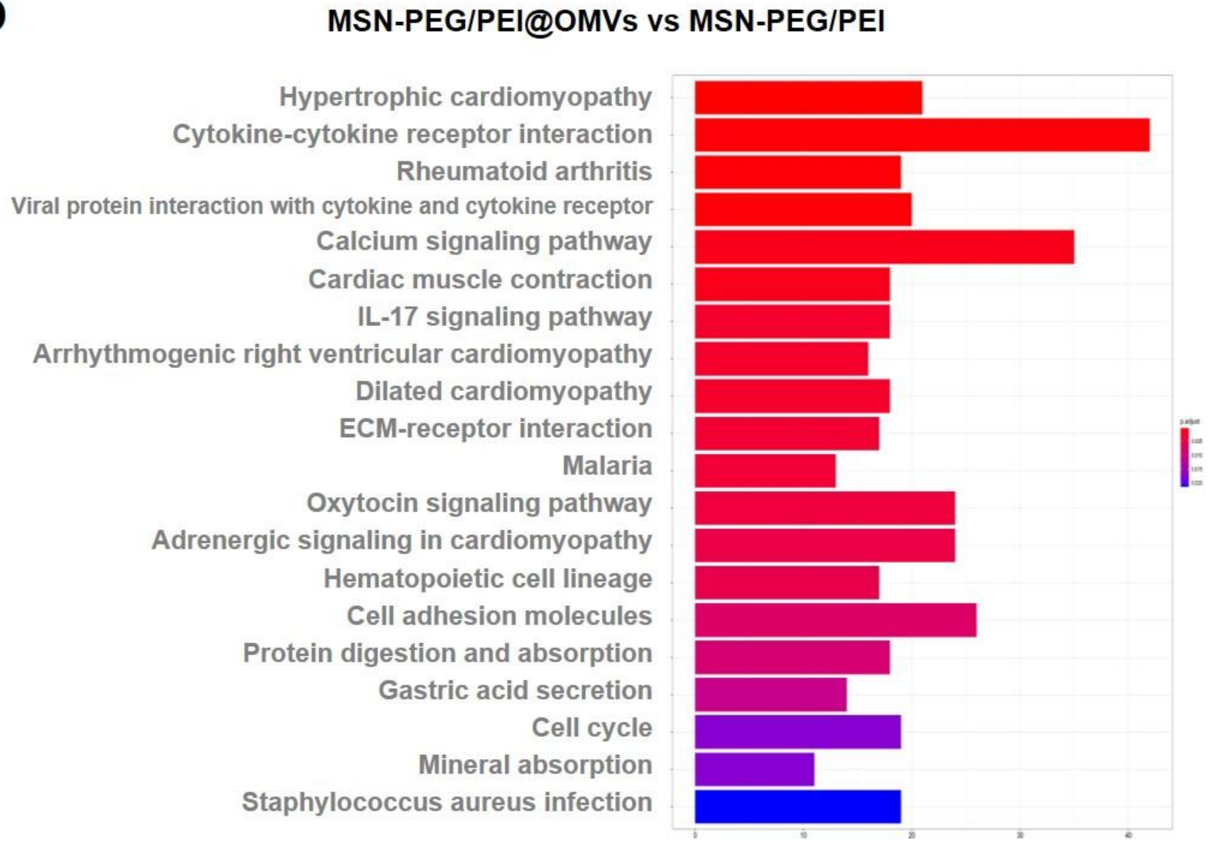
MSN-PEG/PEI@OMVs vs MSN-PEG/PEI
Hypertrophic cardiomyopathy
Cytokine-cytokine receptor interaction
Rheumatoid arthritis
Viral protein interaction with cytokine and cytokine receptor
Calcium signaling pathway
Cardiac muscle contraction
IL-17 signaling pathway
Arrhythmogenic right ventricular cardiomyopathy
Dilated cardiomyopathy
ECM-receptor interaction
Malaria
Oxytocin signaling pathway
Adrenergic signaling in cardiomyopathy
Hematopoietic cell lineage
Cell adhesion molecules
Protein digestion and absorption
Gastric acid secretion
Cell cycle
Mineral absorption
Staphylococcus aureus infection

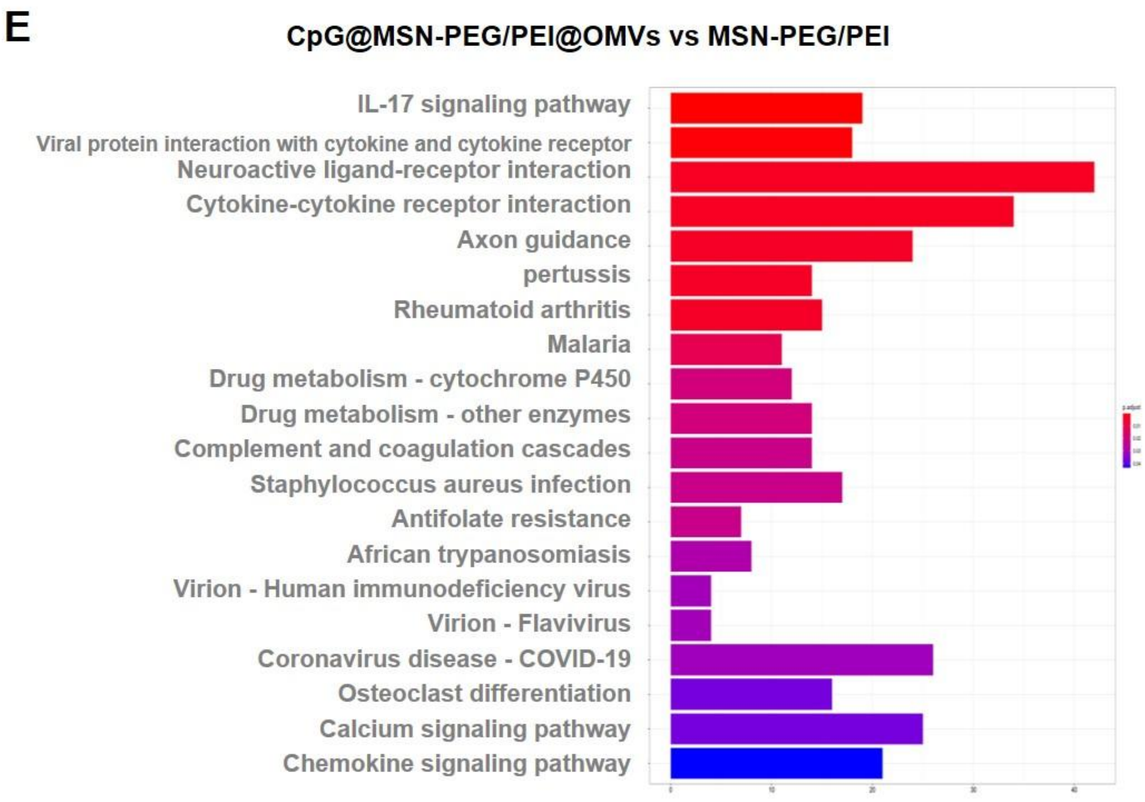

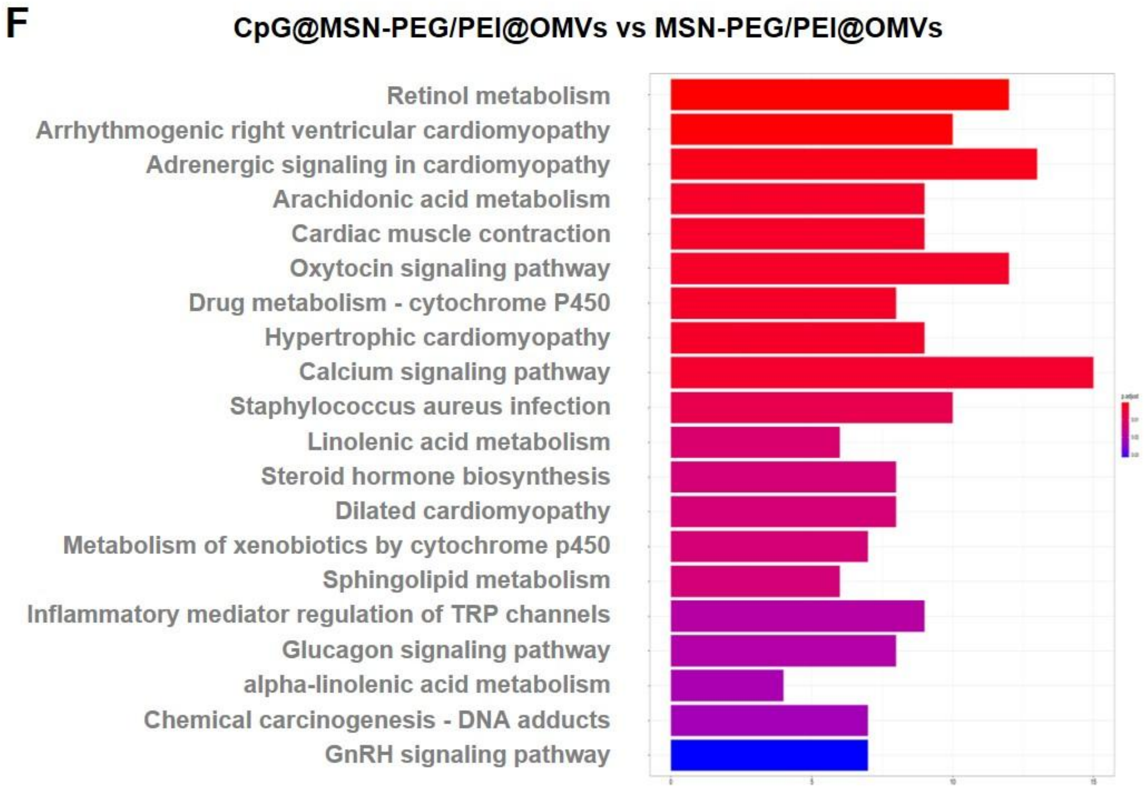

Figure S1-14. Identification of significant KEGG enrichment terms in TME after OMV nanohybrid treatment.

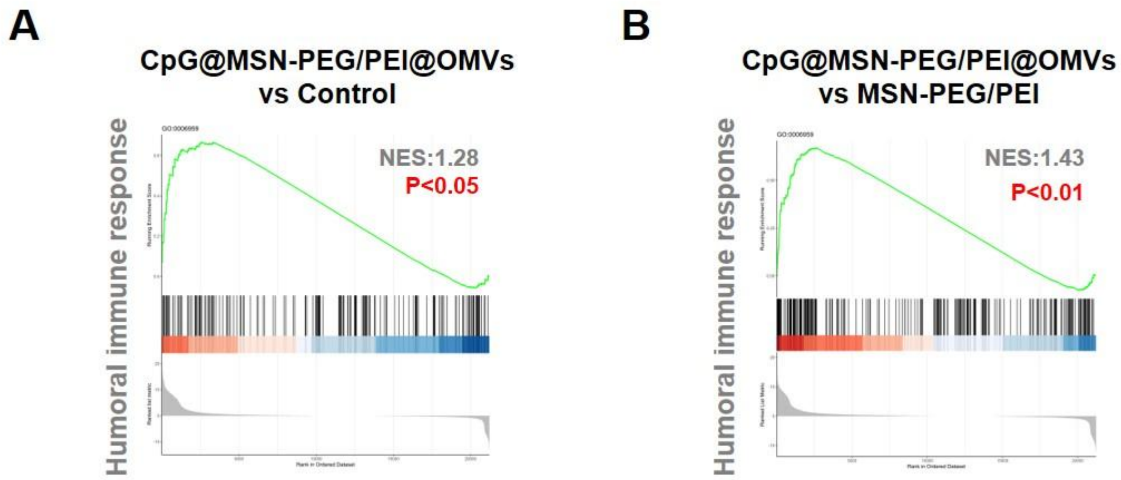

Figure S1-15. GSEA plot of tumor after OMV nanohybrid treatment.

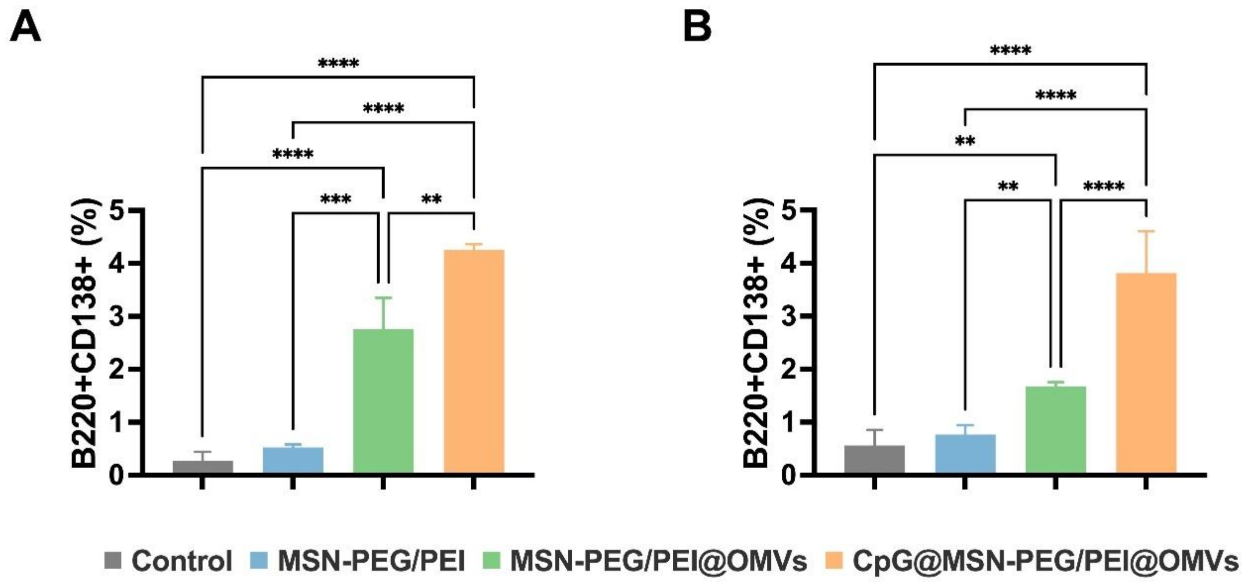

Figure S1-16. Flow cytometric quantification of surface markers CD138 and B220 in spleen and TDLNs after OMV nanohybrid treatment. **(A)** $CD138^{+}B220^{-}$ cell population in spleen **(B)** $CD138^{+}B220^{-}$ cell population in TDLNs isolated from 4T1-bearing BALB/c mice (n = 3 per group).

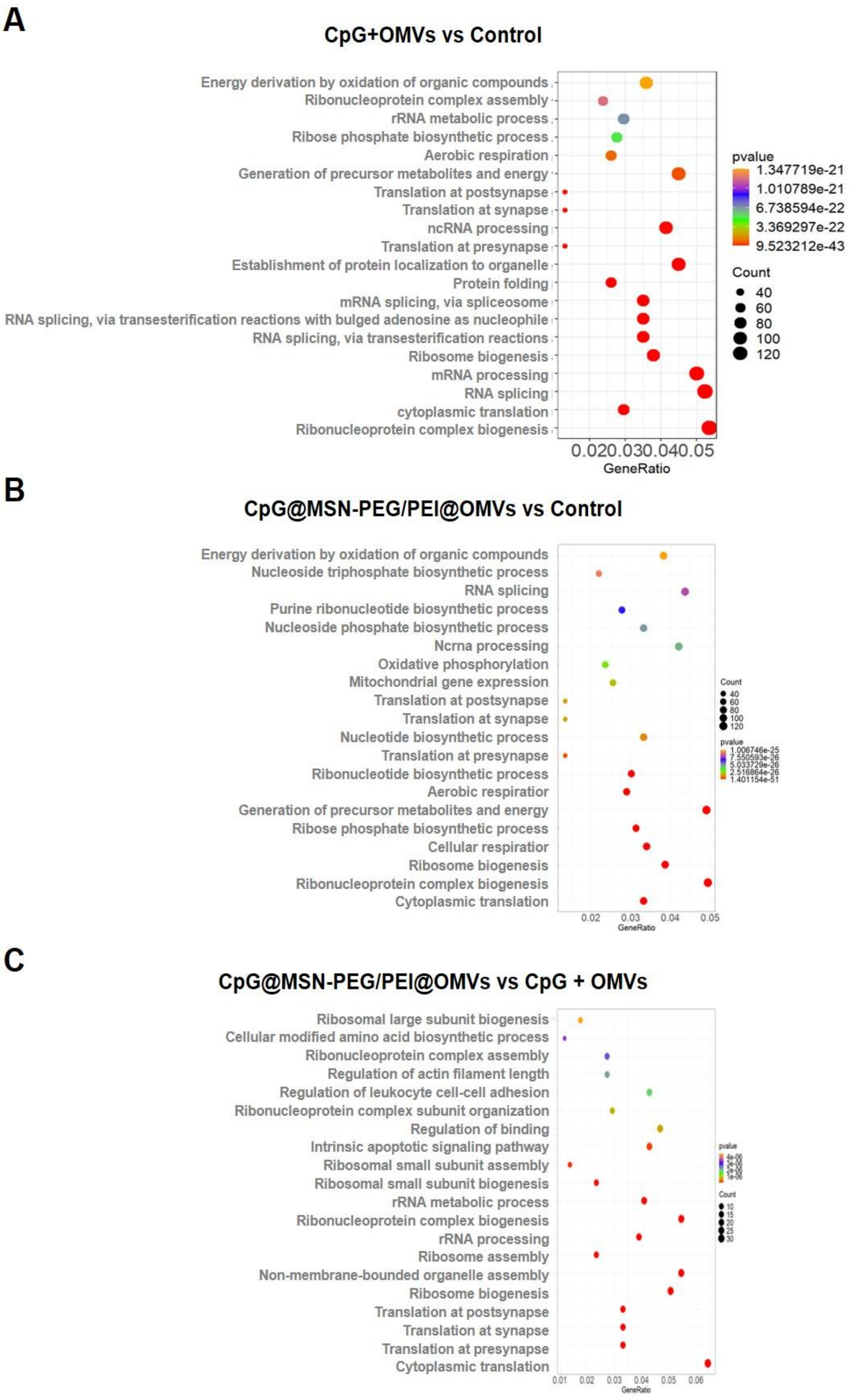

Figure S1-17. GO terms enrichment of biological process on significantly up-regulated pathways of DC subsets from TDLNs after OMV nanohybrid treatment identified by scRNA-seq integration dataset.

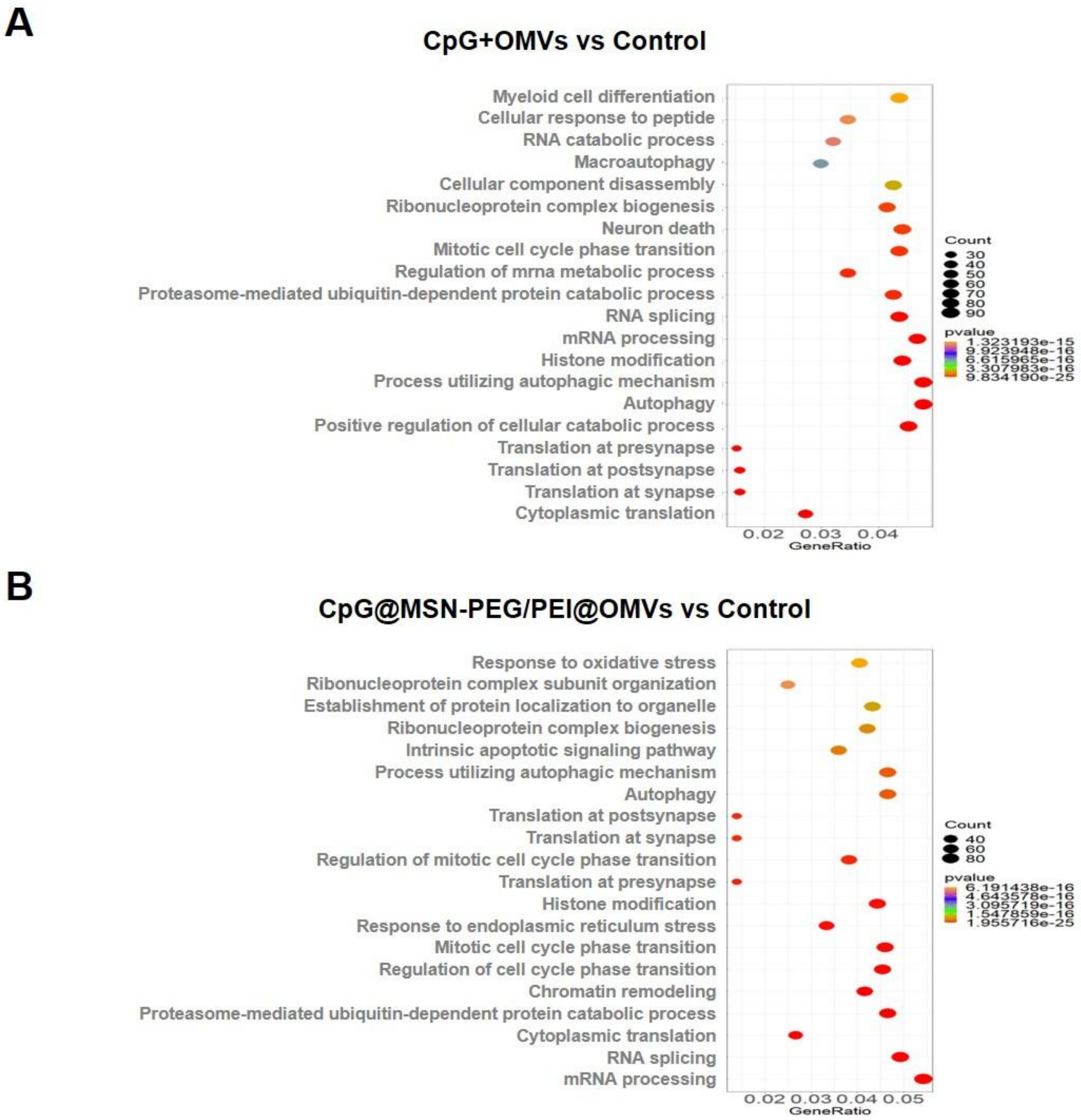

Figure S1-18. GO terms enrichment of biological process on significantly up-regulated pathways of macrophage subsets from TDLNs after OMV nanohybrid treatment identified by scRNA-seq integration dataset.

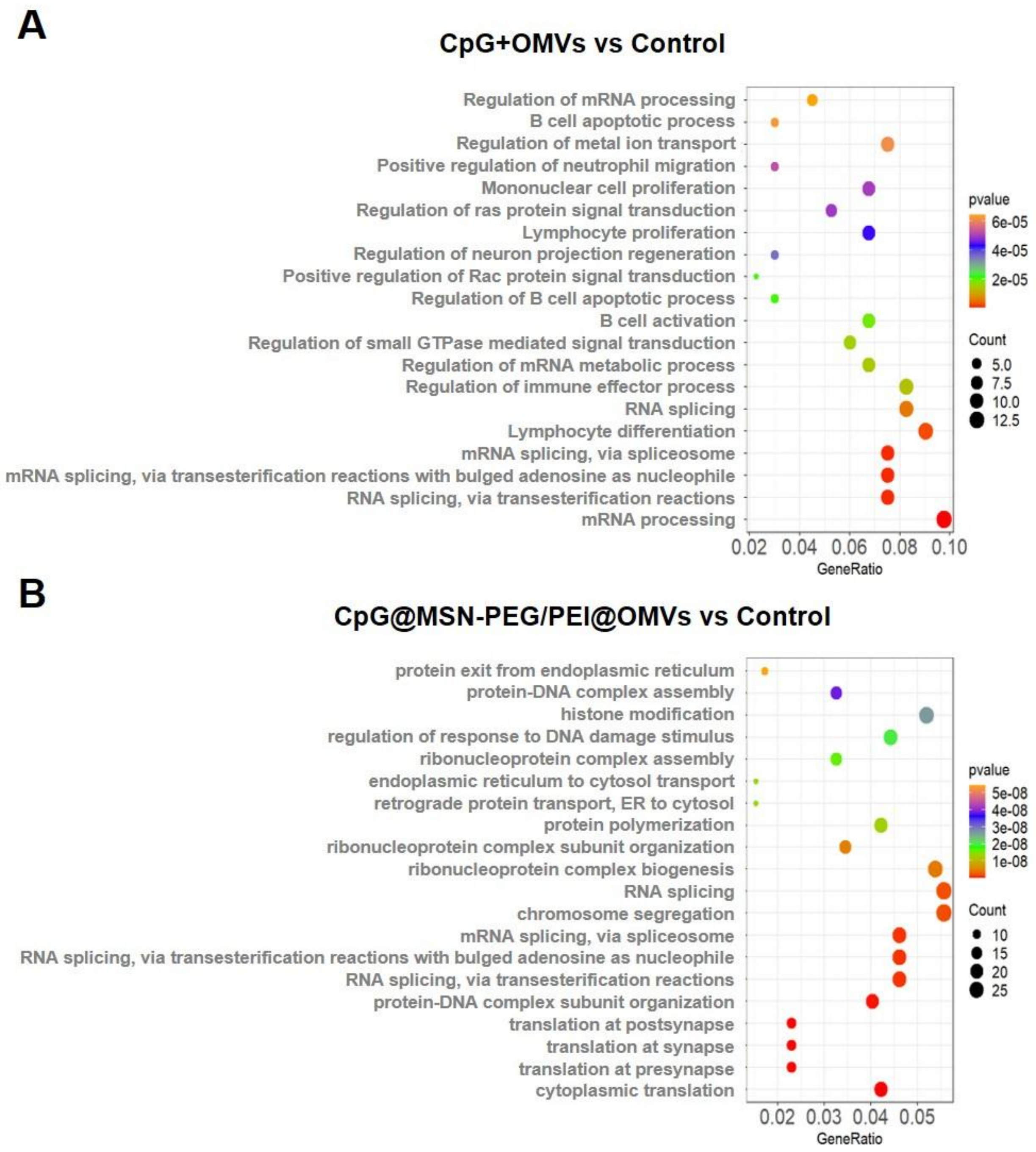

Figure S1-19. GO terms enrichment of biological process on significantly up-regulated pathways of neutrophil subsets from TDLNs after OMV nanohybrid treatment identified by scRNA-seq integration dataset.

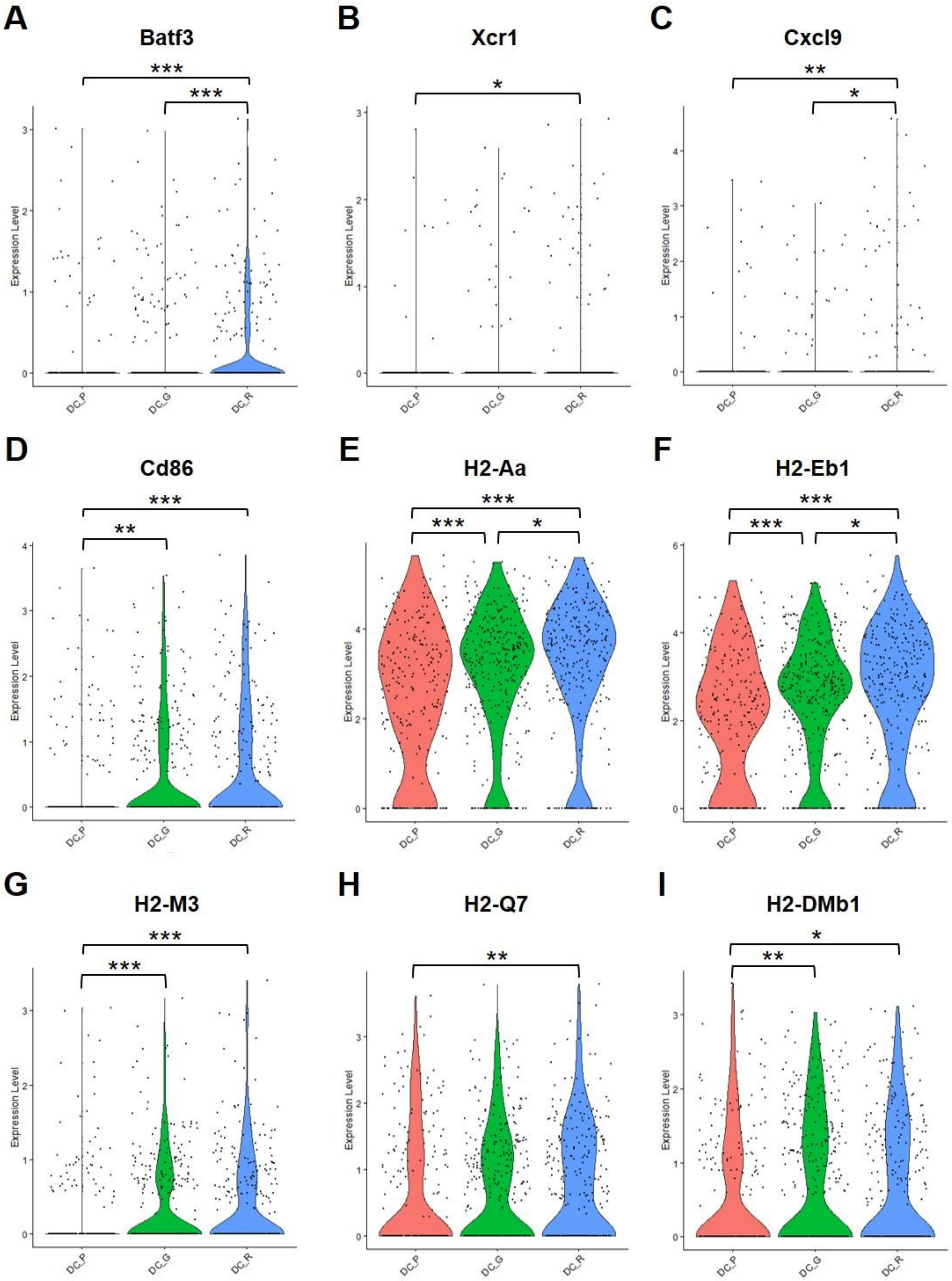

DC_P: Control DC_G: CpG+OMVs DC_ R: CpG@MSN-PEG/PEI@OMVs

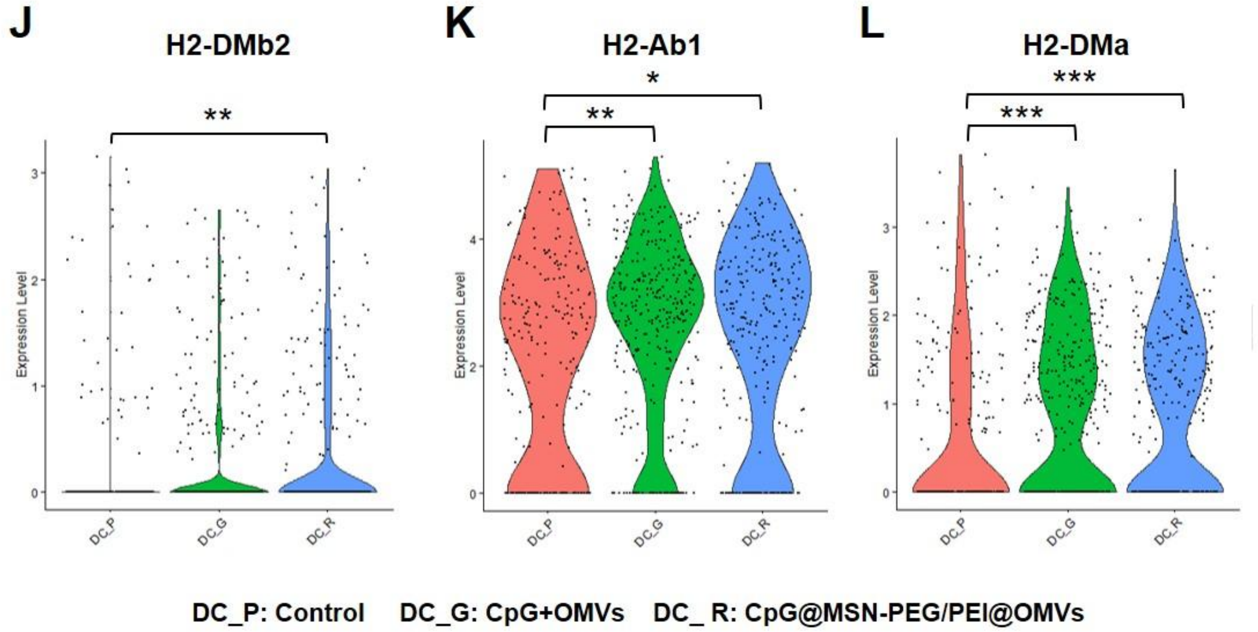

Figure S1-20. Violin plots of normalized expression levels in DC subsets from TDLNs after OMV nanohybrid treatment identified by scRNA-seq integration dataset. **(A)** *Batf3*, **(B)** *Xcr1*, **(C)** *Cxcl9*, **(D)** *Cd86*, **(E)** *H2-Aa*, **(F)** *H2-Eb1*, **(G)** *H2-M3*, **(H)** *H2-Q7*, **(I)** *H2-DMb1,* **(J)** *H2-DMb2,* **(K)** *H2-Ab1, and* **(L)** *H2-DMa*.

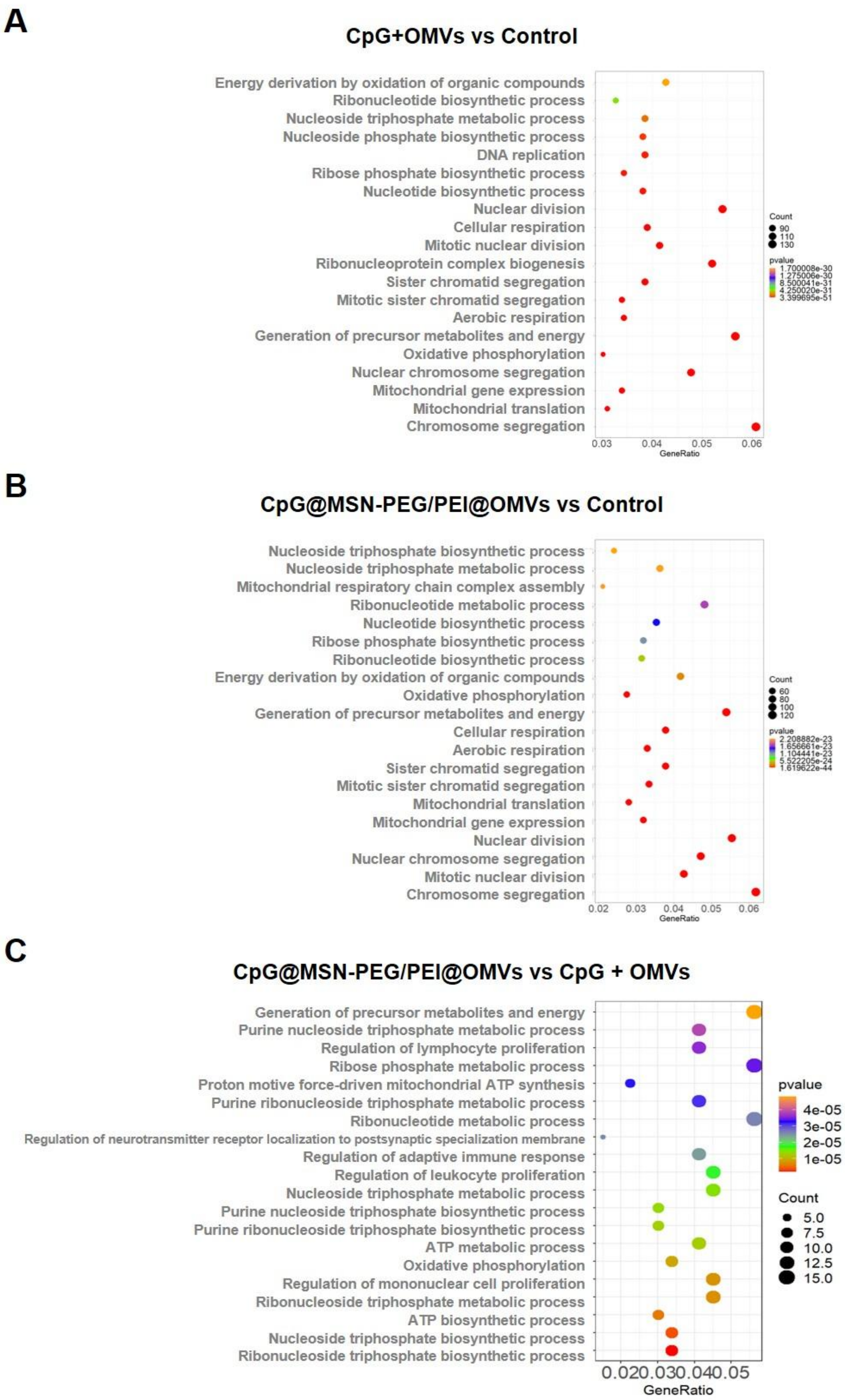

Figure S1-21. GO terms enrichment of biological process on significantly up-regulated pathways of B cell subsets from TDLNs after OMV nanohybrid treatment identified by scRNA-seq integration dataset.

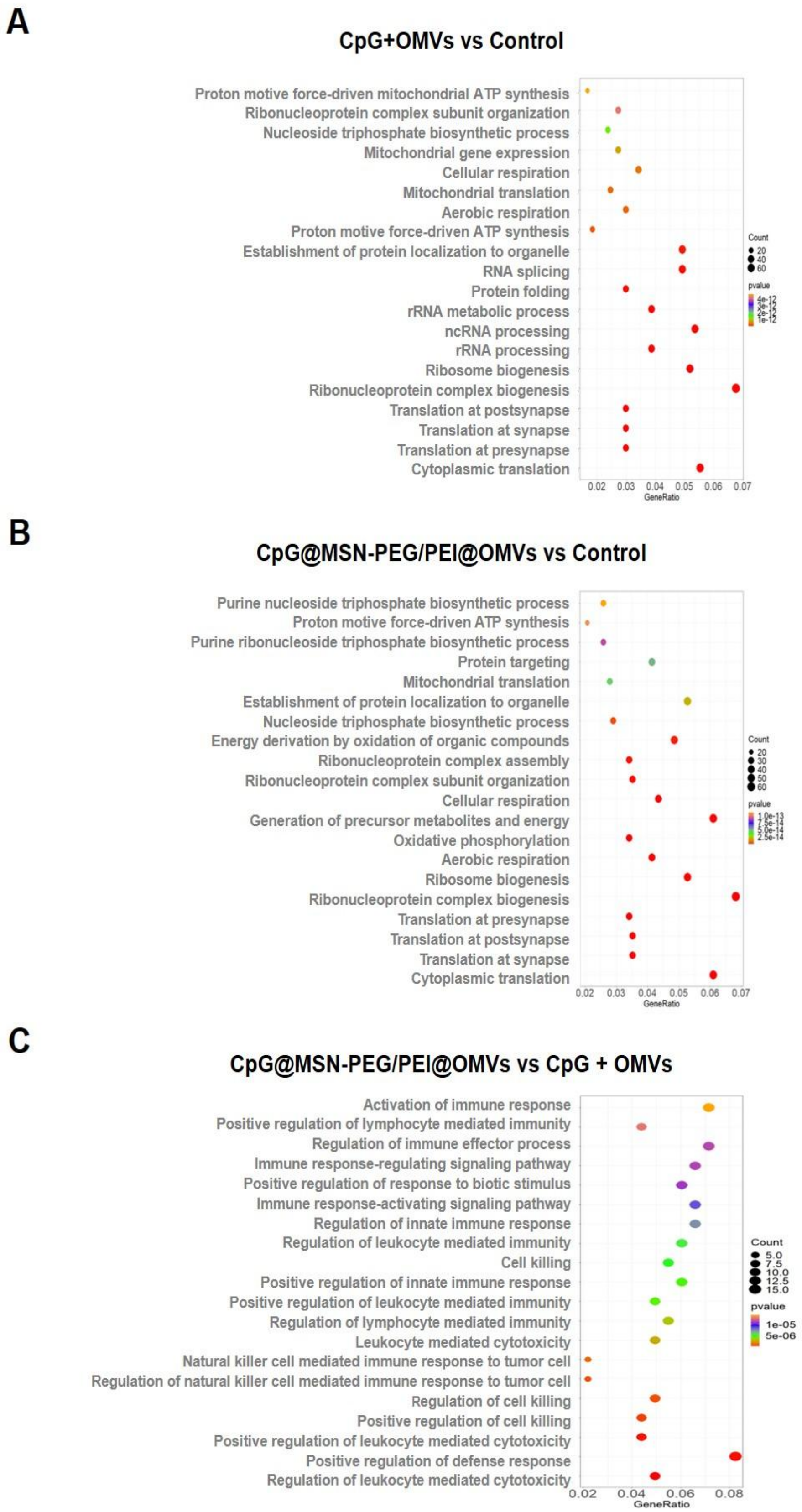

Figure S1-22. GO terms enrichment of biological process on significantly up-regulated pathways of NK cell subsets from TDLNs after OMV nanohybrid treatment identified by scRNA-seq integration dataset.

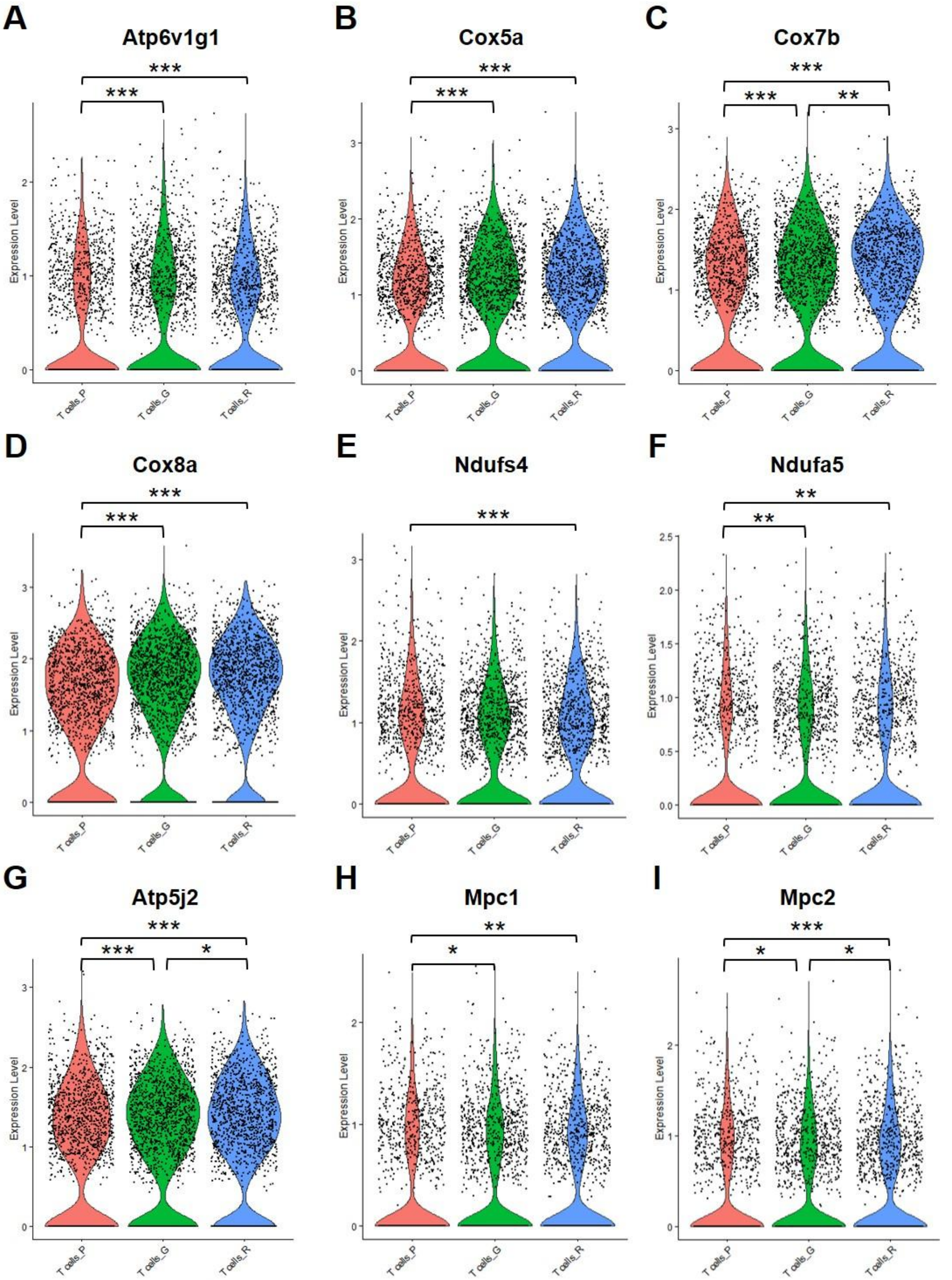

T cells_P: Control T Cells_G: CpG+OMVs T Cells_ R: CpG@MSN-PEG/PEI@OMVs

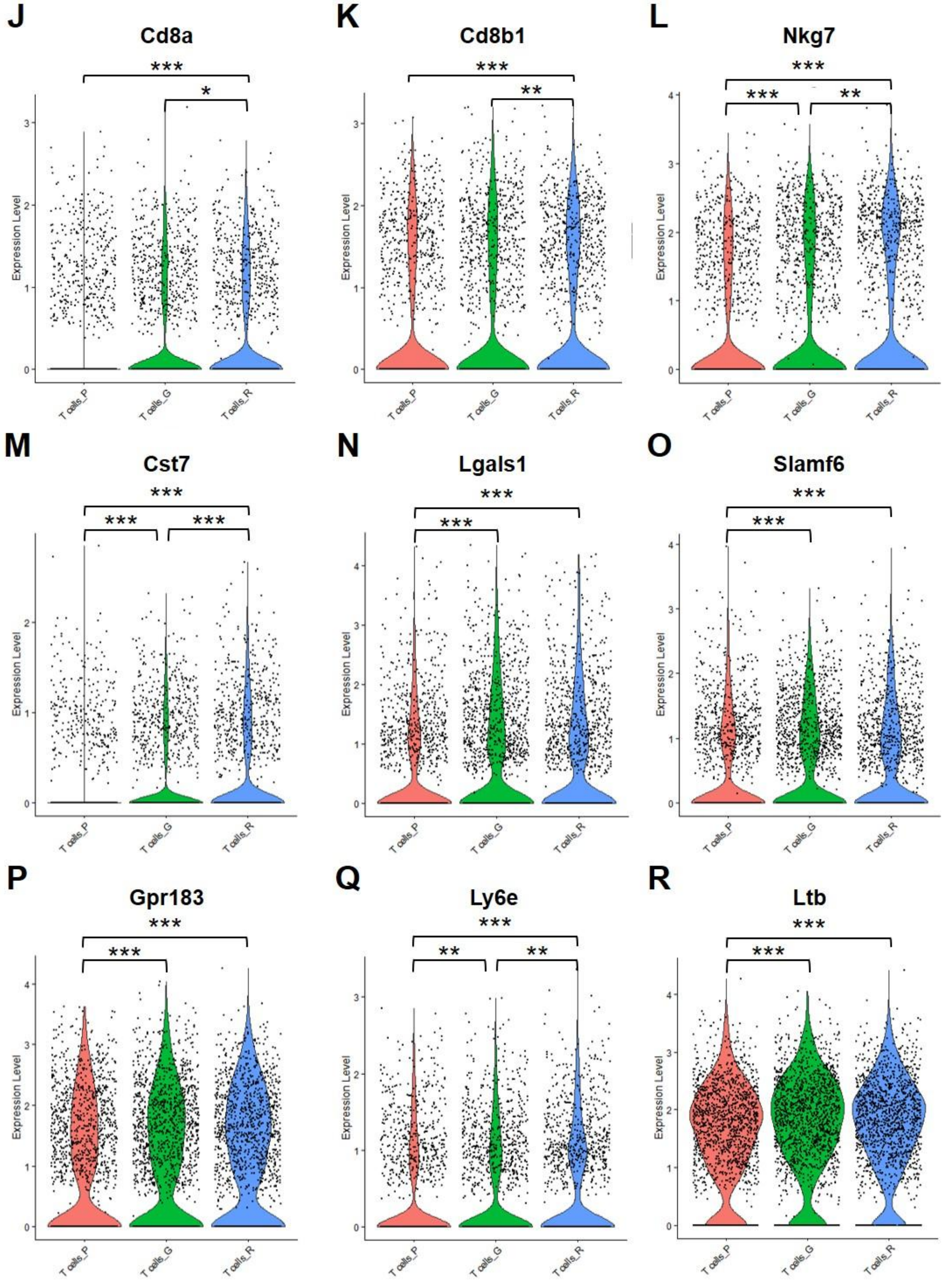

T cells_P: Control T Cells_G: CpG+OMVs T Cells_ R: CpG@MSN-PEG/PEI@OMVs

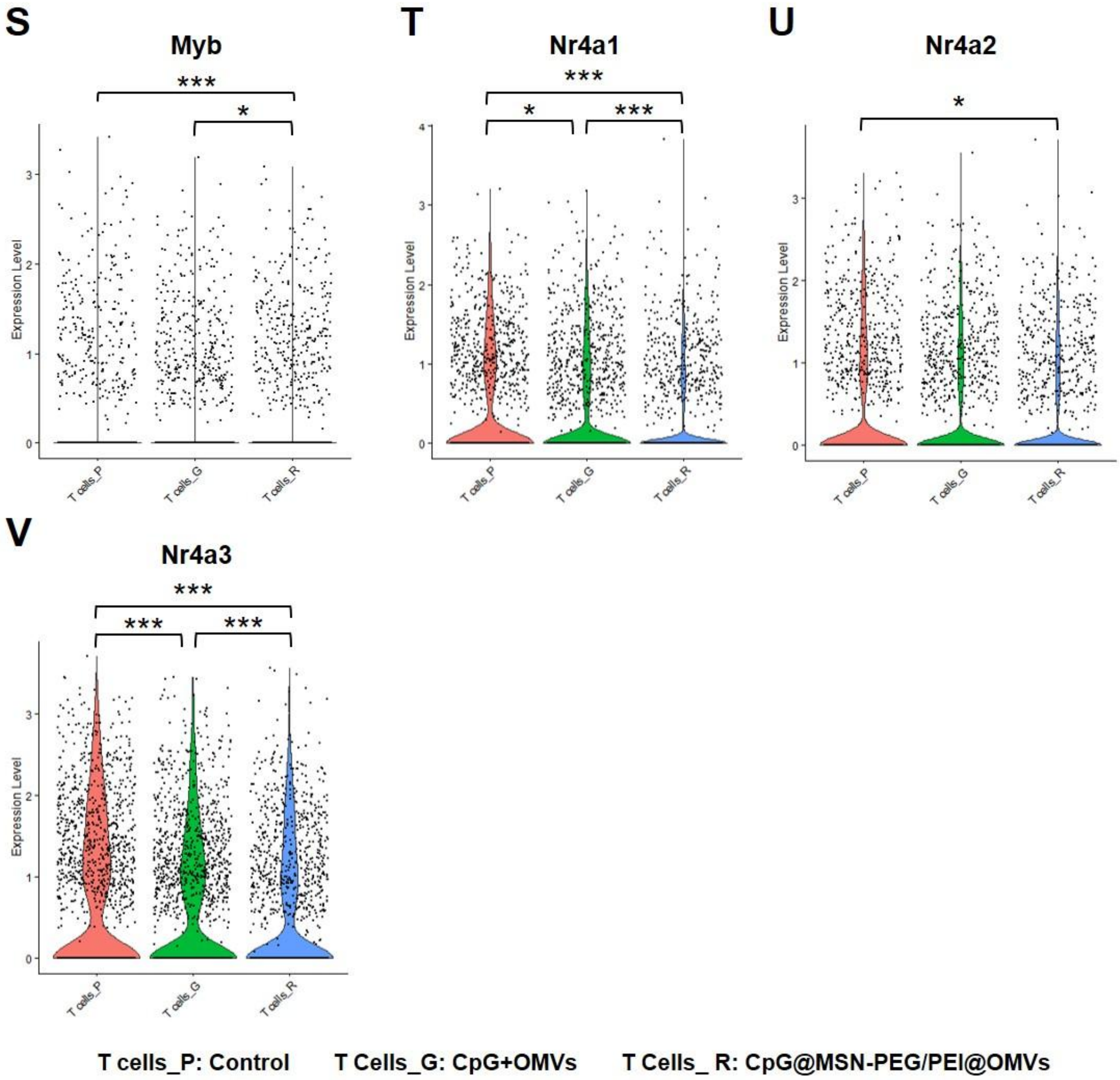

Figure S1-23. Violin plots of normalized expression levels in T cell subsets from TDLNs after OMV nanohybrid treatment identified by scRNA-seq integration dataset. **(A)** *Atp6v1g1*, **(B)** *Cox5a*, **(C)** *Cox7b*, **(D)** *Cox8a*, **(E)** *Ndufs4*, **(F)** *Ndufa5*, **(G)** *Atp5j2*, **(H)** *MPC1*, **(I)** *MPC2,* **(J)** *Cd8a,* **(K)** *Cd8b,* **(L)** *Nkg7*, **(M)** *Cst7*, **(N)** *Lgals1*, **(O)** *Slamf6*, **(P)** *Gpr183*, **(Q)** *Ly6e*, **(R)** *Ltb*, **(S)** *Myb*, **(T)** *Nr4a1*, **(U)** *Nr4a2*, and **(V)** *Nr4a3.*

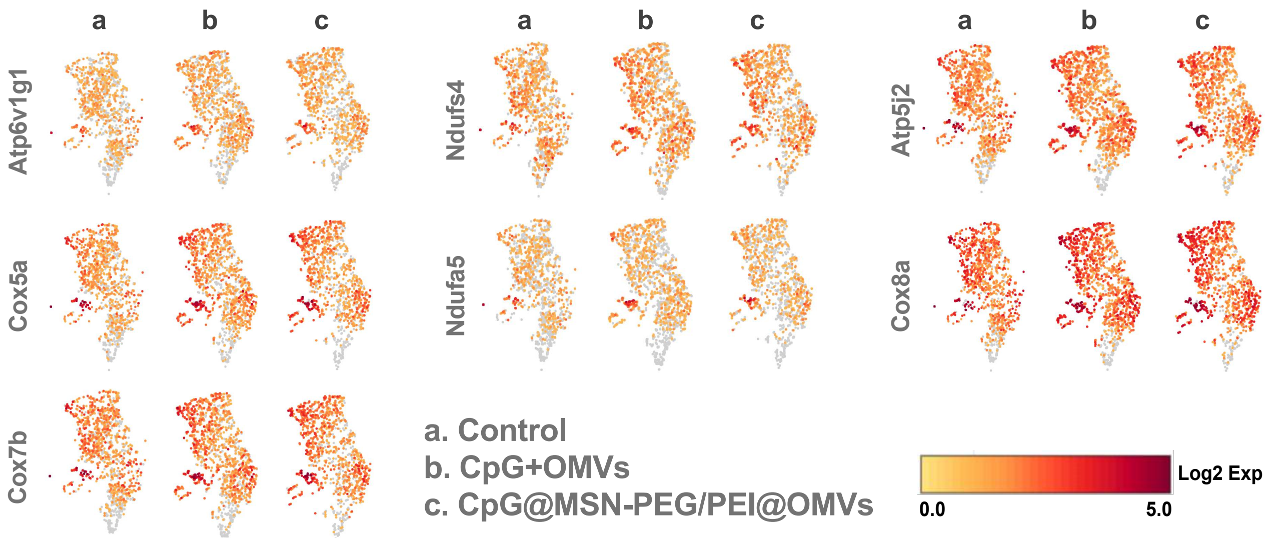

Figure S1-24. Normalized gene expression plot of *Atp6v1g1, Cox5a, Cox7b, Cox8a, Ndufs4, Ndufa5,* and *Atp5j2* in "ETC related to OXPHOS" pathways in T cell subsets from TDLNs after OMV nanohybrid treatment identified by scRNA-seq integration dataset.

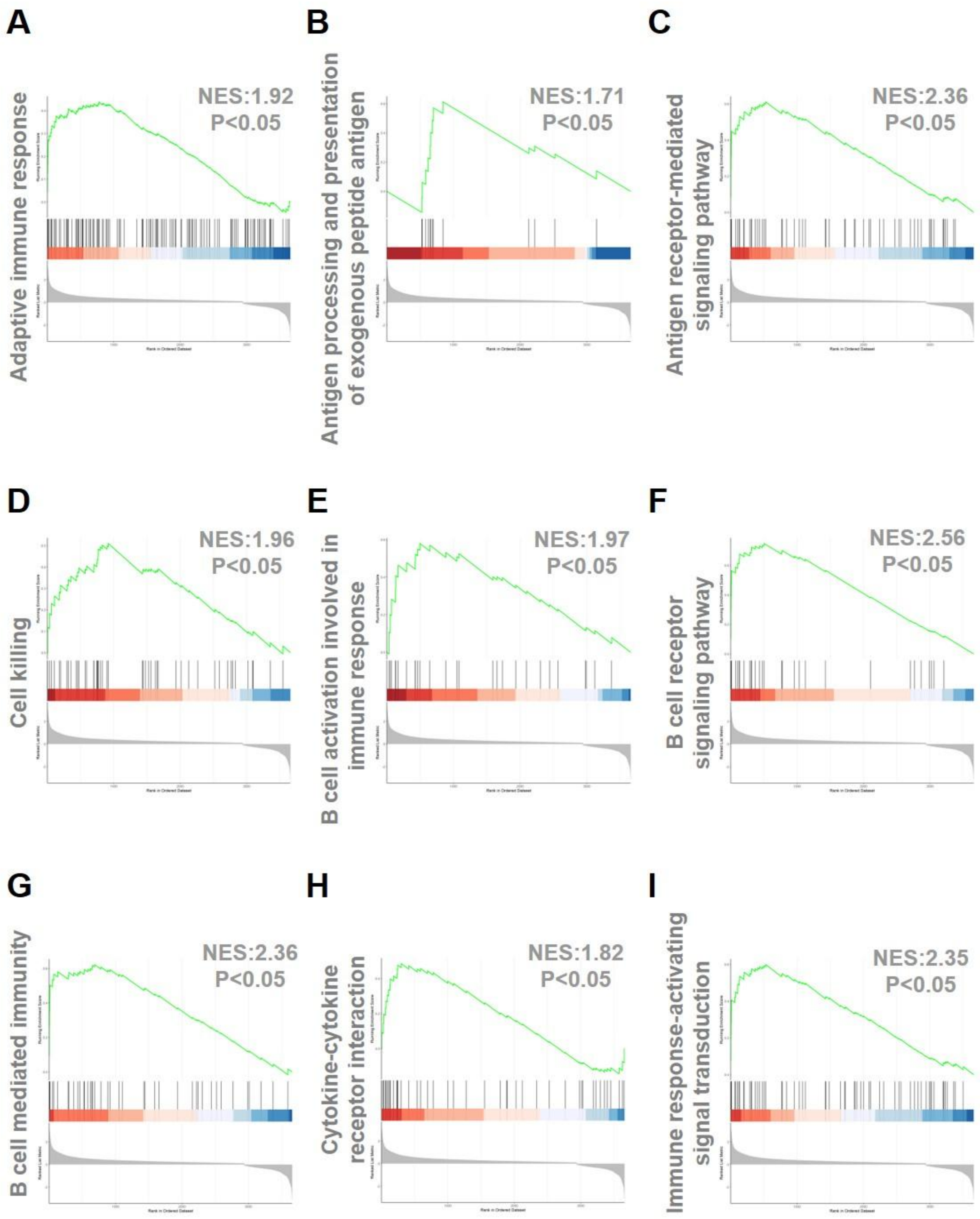
A
Adaptive immune response
NES:1.92
P<0.05
B
Antigen processing and presentation of exogenous peptide antigen
NES:1.71
P<0.05
C
Antigen receptor-mediated signaling pathway
NES:2.36
P<0.05
D
Cell killing
NES:1.96
P<0.05
E
B cell activation involved in immune response
NES:1.97
P<0.05
F
B cell receptor signaling pathway
NES:2.56
P<0.05
G
B cell mediated immunity
NES:2.36
P<0.05
H
Cytokine-cytokine receptor interaction
NES:1.82
P<0.05
I
Immune response-activating signal transduction
NES:2.35
P<0.05

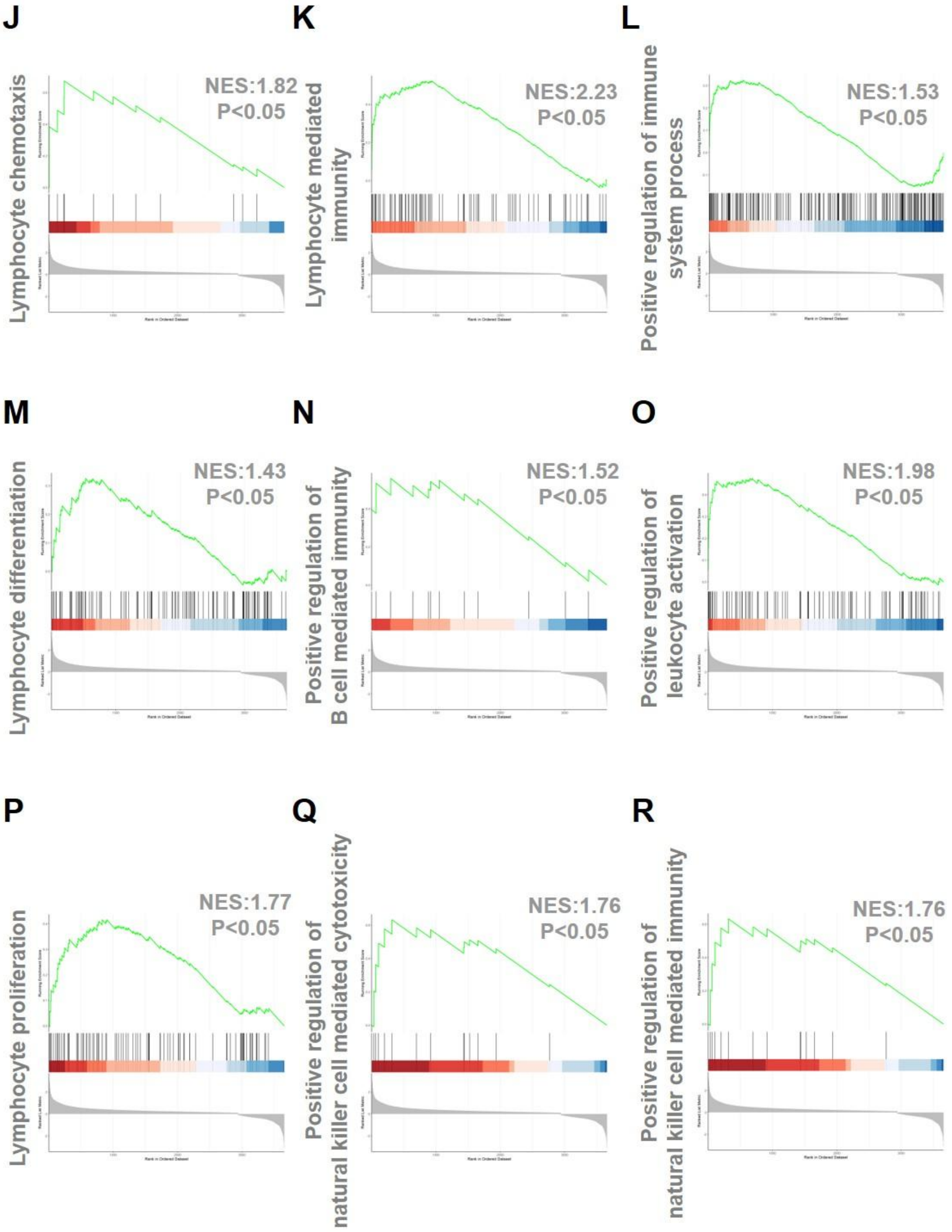

Figure S1-25. GSEA plots of T cell subsets from TDLNs after CpG@MSN-PEG/PEI@OMV treatment identified by scRNA-seq integration dataset and subsequently compared between CpG@MSN-PEG/PEI@OMVs versus control. NES: normalized enrichment score.

Table S1. Complete blood count (CBC). Blood collected via cardiac puncture performed in hematological parameters.

Table. Complete blood count (CBC)

CBC (Mean ± SD)	Naive (no tumor)	Control (with tumor)	MSN-PEG/PEI	MSN-PEG/PEI@OMVs	CpG@MSN-PEG/PEI@OMVs
WBC (K/μL)	2.6 ± 0.2	231.5 ± 61.8	302 ± 35.6	204.6 ± 42.5	185 ± 82.4
HGB (g/dL)	15.5 ± 0.2	13.4 ± 0.2	13.4 ± 1.1	13.1 ± 0.2	13.4 ± 0.2
RBC (M/μL)	10.3 ± 0.1	9.5 ± 0.2	9.9 ± 0.6	9.5 ± 0.2	9.7 ± 0.2
NEU (K/μL)	0.7 ± 0.3	155.7 ± 46.1	194.6 ± 30.1	134.6 ± 32	125.1 ± 61.7
LYM (K/μL)	1.9 ± 0.3	29.5 ± 3.3	40 ± 2.9	30.9 ± 2	26.3 ± 9.2
MONO (%)	0.7 ± 0.3	17.6 ± 1.7	20.5 ± 0.5	19.5 ± 0.7	18 ± 0.7
HCT (%)	51.7 ± 0.4	47.6 ± 0.1	47.3 ± 3	46.6 ± 1.2	48.4 ± 1.7
MCV (fL)	50 ± 0.3	51.3 ± 0.2	50.9 ± 0.5	48.9 ± 0.5	50 ± 0.8
MCH (pg)	15 ± 0.3	13.8 ± 0.5	13.7 ± 0.2	13.7 ± 0.06	13.8 ± 0.1
MCHC (g/dL)	29.9 ± 0.3	27.9 ± 0.6	27.1 ± 0.6	28.1 ± 0.4	27.6 ± 0.3
RDW (%)	25.3 ± 0.4	25.1 ± 0.2	25.6 ± 0.4	26.5 ± 0.5	26.2 ± 0.6
PLT (K/μL)	834.3 ± 112.7	827.7 ± 20	799 ± 58.5	958.3 ± 21	943 ± 17
PDW (fL)	10.3 ± 1.6	8.8 ± 0.3	8.9 ± 0.2	8.2 ± 0.6	8.4 ± 0.5
PCT (%)	0.8 ± 0.1	0.8 ± 0.02	0.7 ± 0.1	0.9 ± 0.02	0.8 ± 0.03
MPV (fL)	9.9 ± 0.4	9.3 ± 0.1	9.1 ± 0.1	9.1 ± 0.1	9 ± 0.2

Supporting Information 2

(Raw flow cytometry plots and gating strategy)

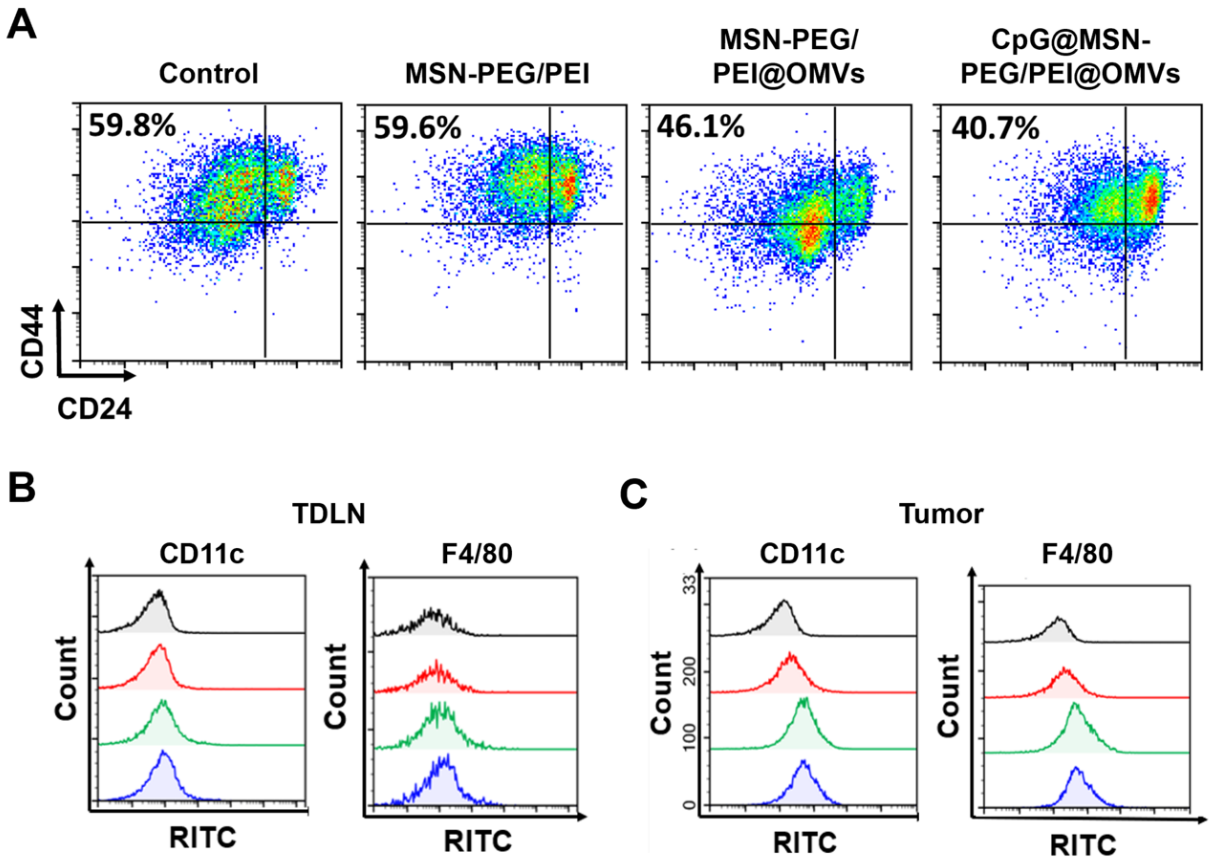

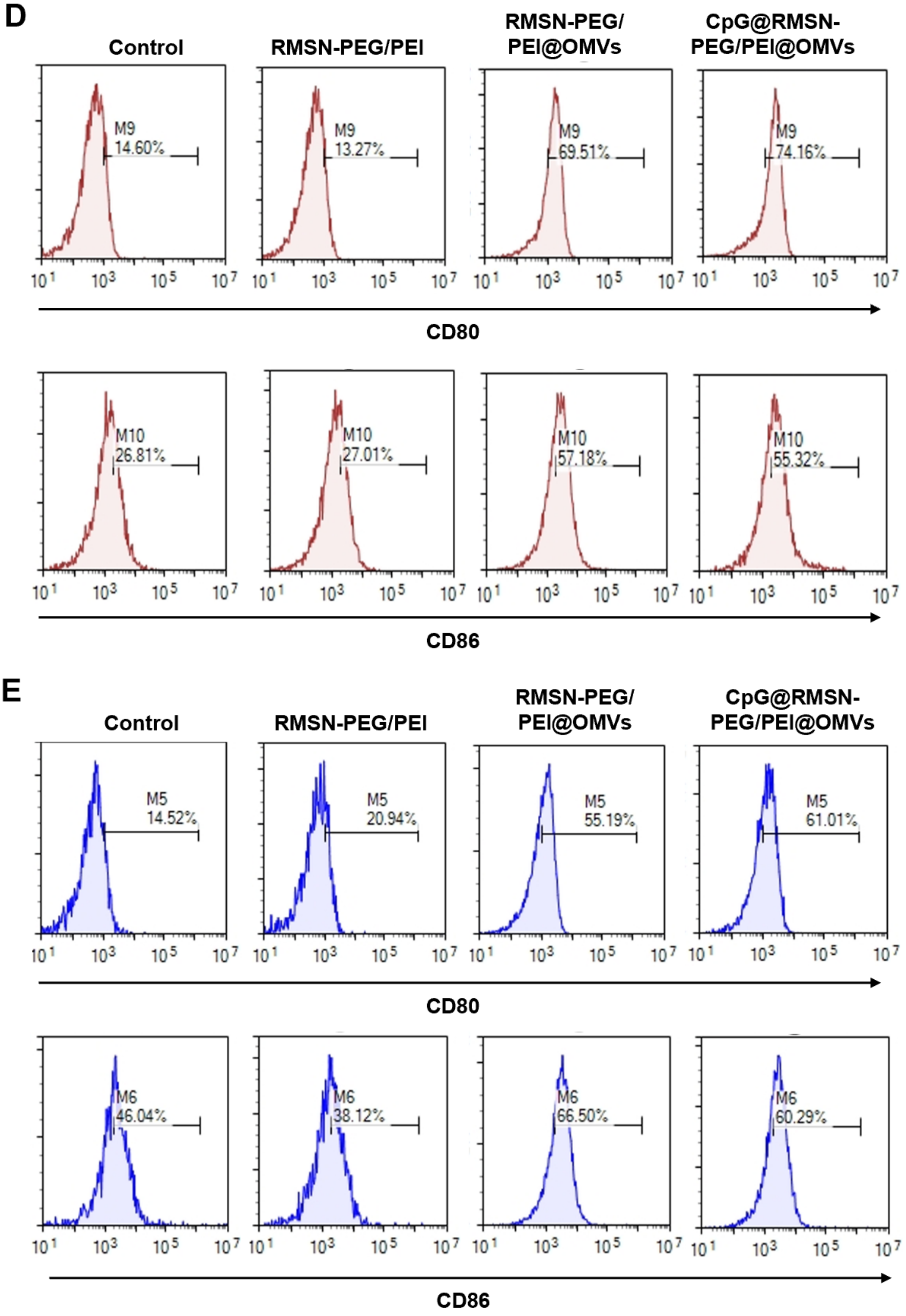
D
Control
RMSN-PEG/PEI
RMSN-PEG/PEI@OMVs
CpG@RMSN-PEG/PEI@OMVs
M9 14.60%
M9 13.27%
M9 69.51%
M9 74.16%
CD80
M10 26.81%
M10 27.01%
M10 57.18%
M10 55.32%
CD86
E
Control
RMSN-PEG/PEI
RMSN-PEG/PEI@OMVs
CpG@RMSN-PEG/PEI@OMVs
M5 14.52%
M5 20.94%
M5 55.19%
M5 61.01%
CD80
M6 46.04%
M6 38.12%
M6 66.50%
M6 60.29%
CD86

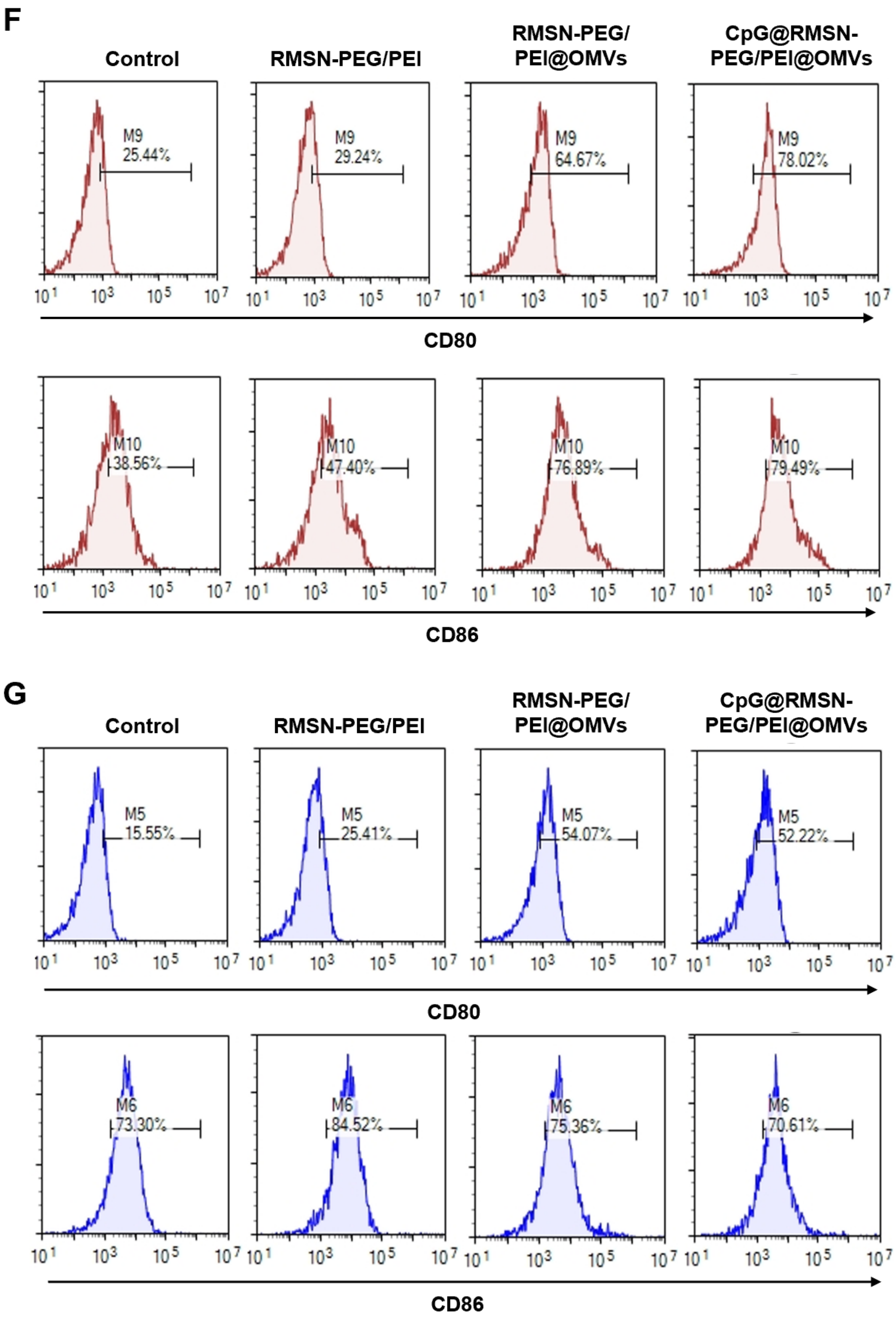

F
Control
RMSN-PEG/PEI
RMSN-PEG/ PEI@OMVs
CpG@RMSN-PEG/PEI@OMVs
M9 25.44%
M9 29.24%
M9 64.67%
M9 78.02%
CD80
M10 38.56%
M10 47.40%
M10 76.89%
M10 79.49%
CD86
G
Control
RMSN-PEG/PEI
RMSN-PEG/ PEI@OMVs
CpG@RMSN-PEG/PEI@OMVs
M5 15.55%
M5 25.41%
M5 54.07%
M5 52.22%
CD80
M6 73.30%
M6 84.52%
M6 75.36%
M6 70.61%
CD86

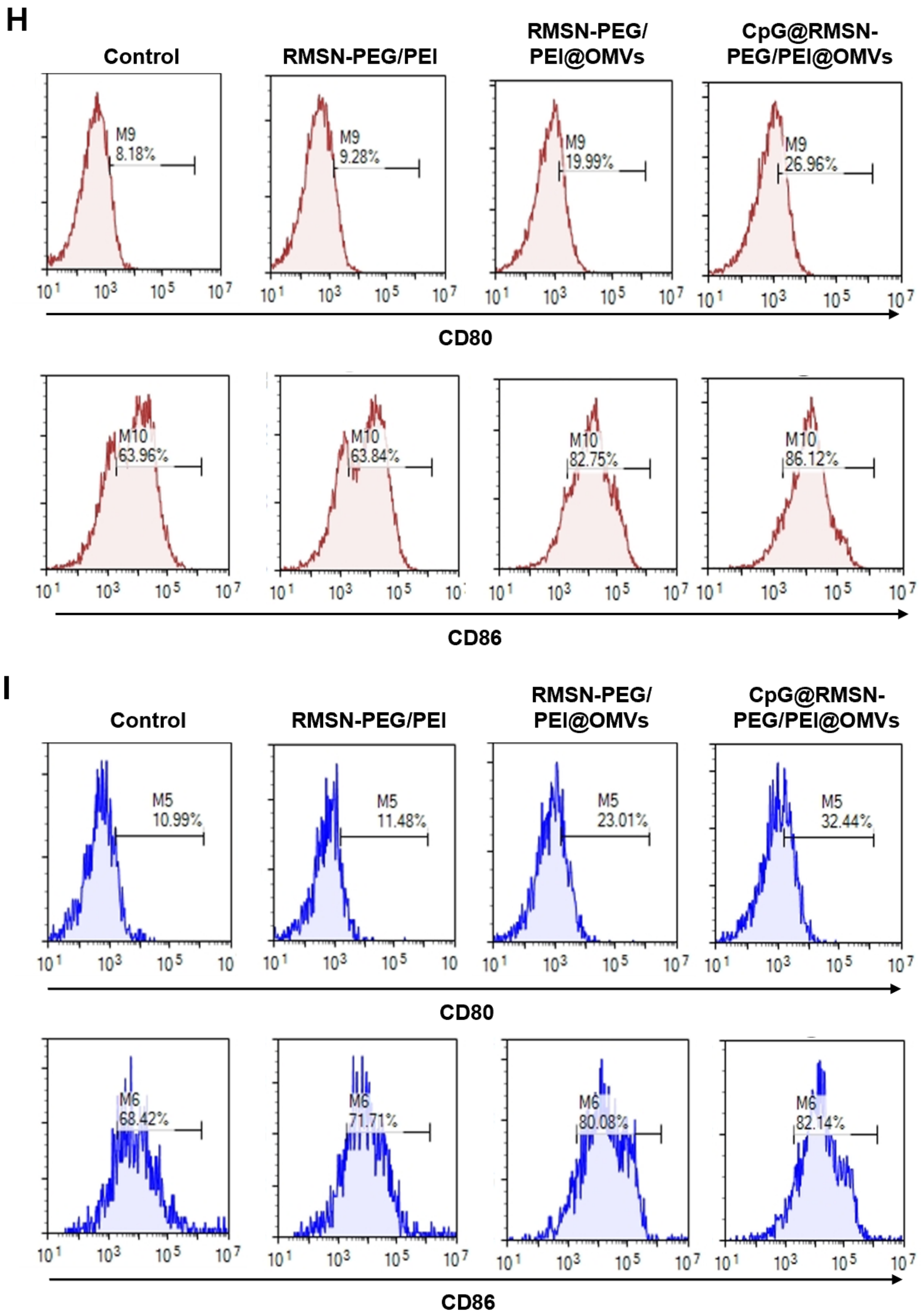
H
Control
RMSN-PEG/PEI
RMSN-PEG/ PEI@OMVs
CpG@RMSN- PEG/PEI@OMVs
M9 8.18%
M9 9.28%
M9 19.99%
M9 26.96%
CD80
M10 63.96%
M10 63.84%
M10 82.75%
M10 86.12%
CD86
I
Control
RMSN-PEG/PEI
RMSN-PEG/ PEI@OMVs
CpG@RMSN- PEG/PEI@OMVs
M5 10.99%
M5 11.48%
M5 23.01%
M5 32.44%
CD80
M6 68.42%
M6 71.71%
M6 80.08%
M6 82.14%
CD86

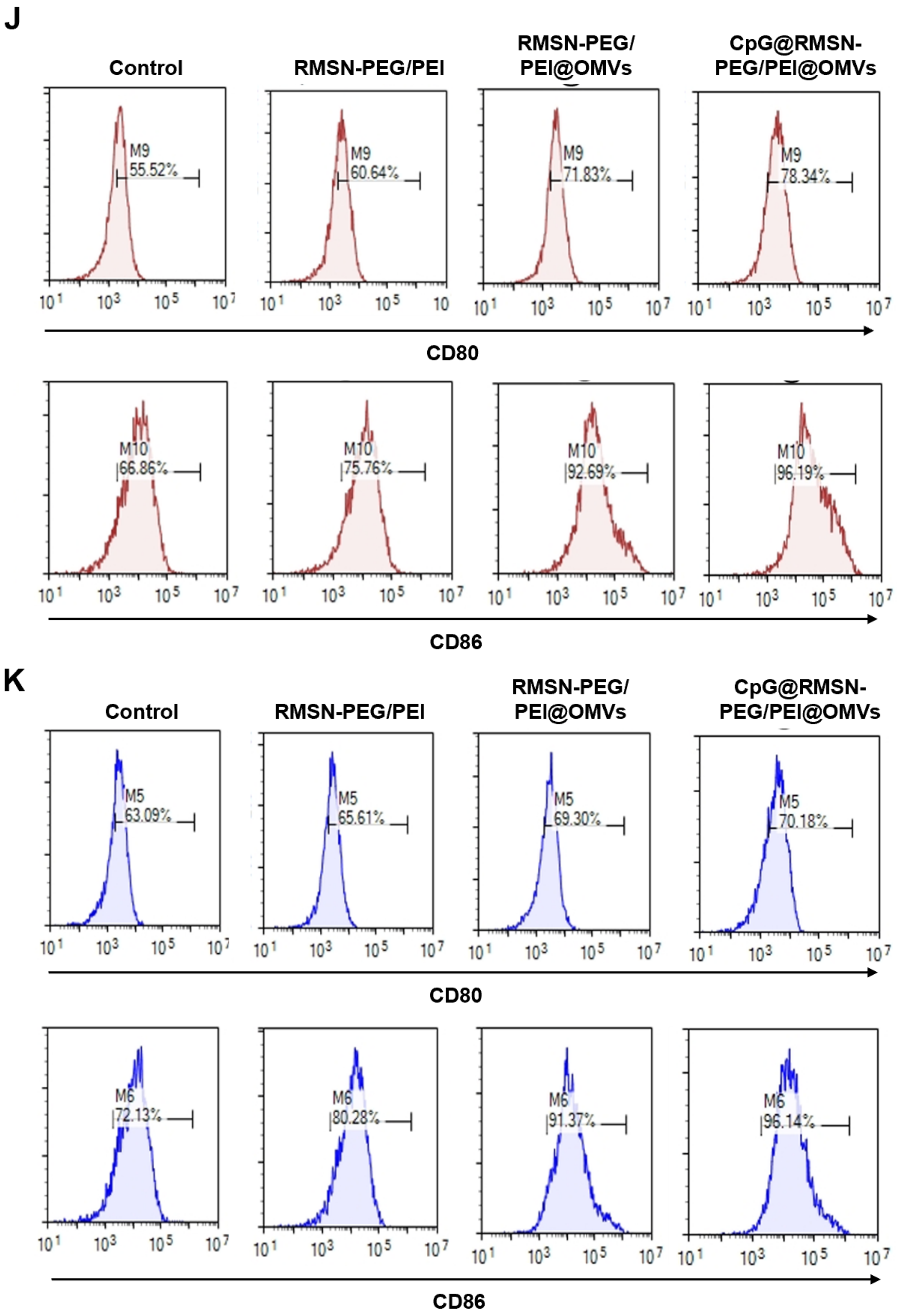
J
Control
RMSN-PEG/PEI
RMSN-PEG/PEI@OMVs
CpG@RMSN-PEG/PEI@OMVs
M9 55.52%
M9 60.64%
M9 71.83%
M9 78.34%
CD80
M10 66.86%
M10 75.76%
M10 92.69%
M10 96.19%
CD86
K
Control
RMSN-PEG/PEI
RMSN-PEG/PEI@OMVs
CpG@RMSN-PEG/PEI@OMVs
M5 63.09%
M5 65.61%
M5 69.30%
M5 70.18%
CD80
M6 72.13%
M6 80.28%
M6 91.37%
M6 96.14%
CD86

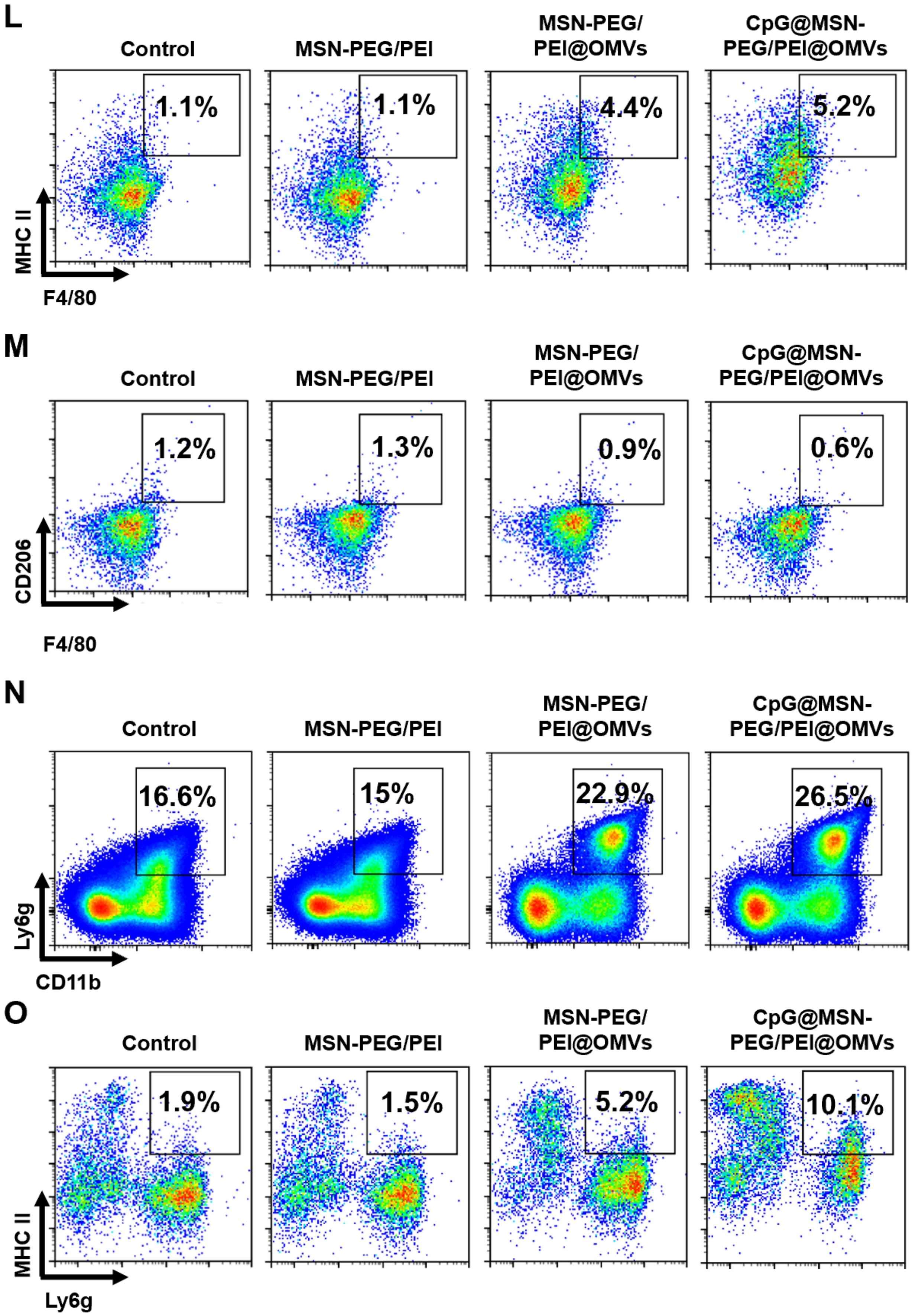
L
Control
MSN-PEG/PEI
MSN-PEG/PEI@OMVs
CpG@MSN-PEG/PEI@OMVs
1.1%
1.1%
4.4%
5.2%
MHC II
F4/80
M
Control
MSN-PEG/PEI
MSN-PEG/PEI@OMVs
CpG@MSN-PEG/PEI@OMVs
1.2%
1.3%
0.9%
0.6%
CD206
F4/80
N
Control
MSN-PEG/PEI
MSN-PEG/PEI@OMVs
CpG@MSN-PEG/PEI@OMVs
16.6%
15%
22.9%
26.5%
Ly6g
CD11b
O
Control
MSN-PEG/PEI
MSN-PEG/PEI@OMVs
CpG@MSN-PEG/PEI@OMVs
1.9%
1.5%
5.2%
10.1%
MHC II
Ly6g

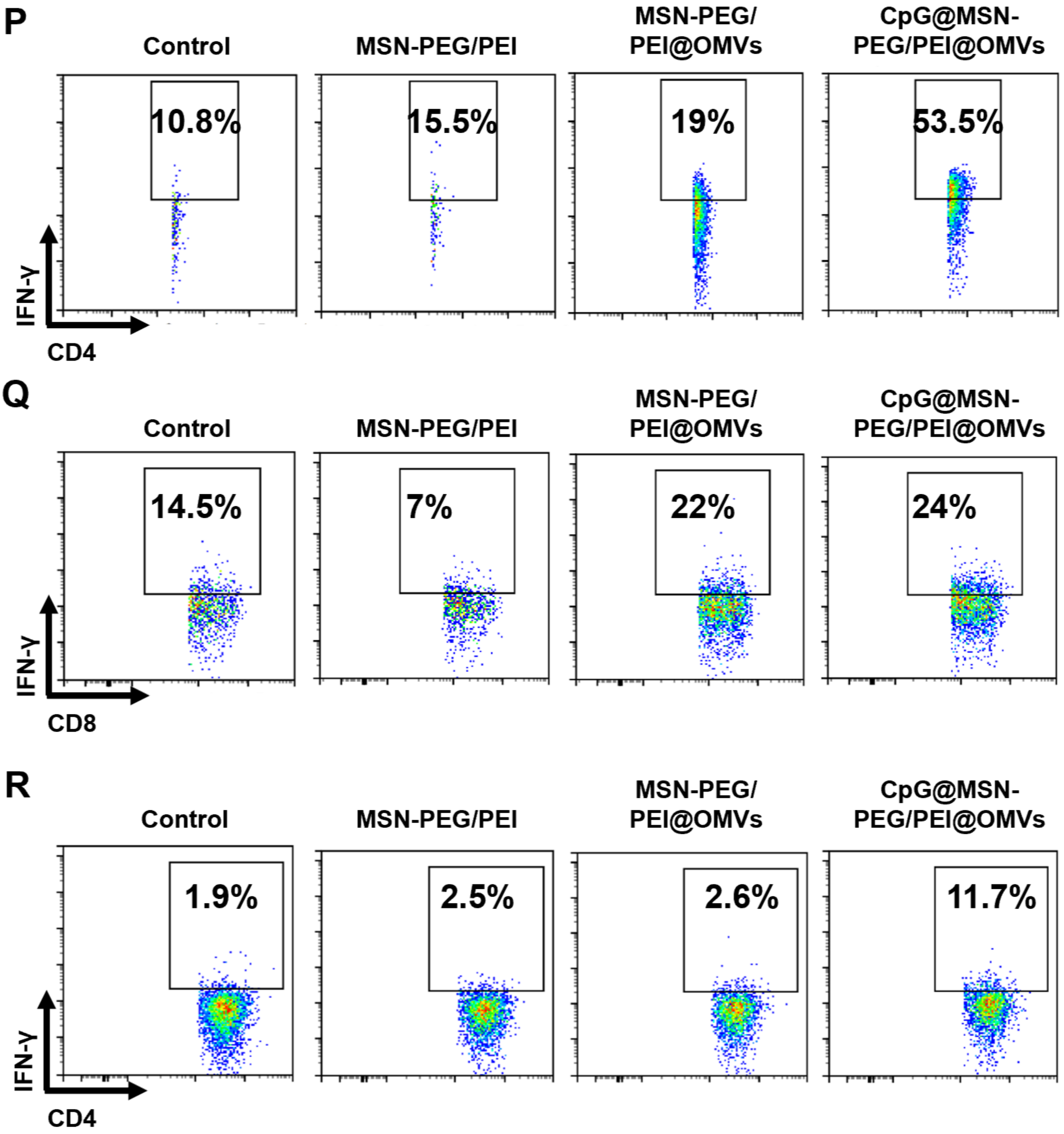
P
Control
MSN-PEG/PEI
MSN-PEG/PEI@OMVs
CpG@MSN-PEG/PEI@OMVs
10.8%
15.5%
19%
53.5%
IFN-γ
CD4
Q
Control
MSN-PEG/PEI
MSN-PEG/PEI@OMVs
CpG@MSN-PEG/PEI@OMVs
14.5%
7%
22%
24%
IFN-γ
CD8
R
Control
MSN-PEG/PEI
MSN-PEG/PEI@OMVs
CpG@MSN-PEG/PEI@OMVs
1.9%
2.5%
2.6%
11.7%
IFN-γ
CD4

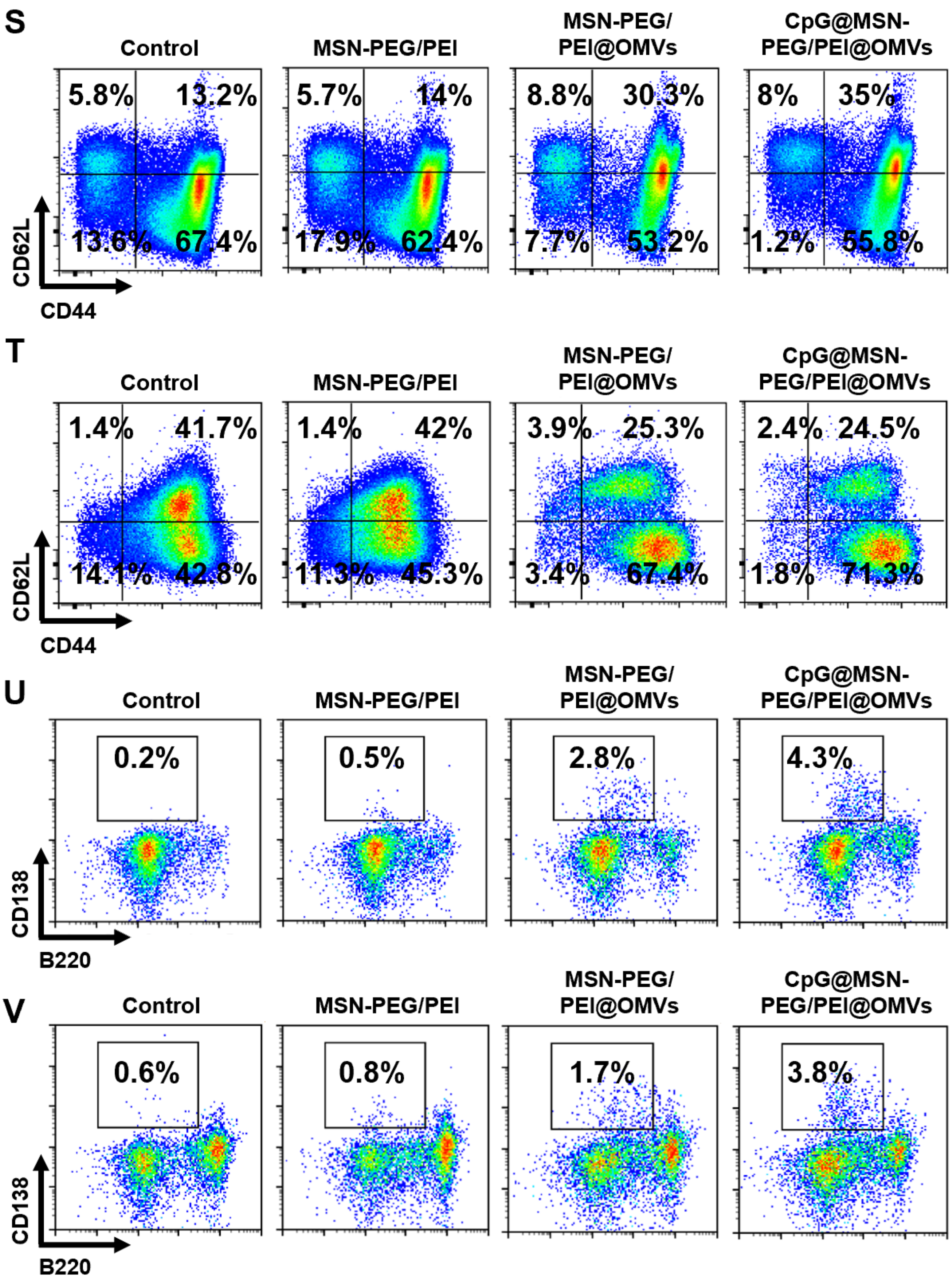

Figure S2-1. (A) Flow cytometry-based measurement of cancer stem-like cell ($CD44^{+}CD24^{-}$) populations in tumor collected at 23 days post two injections of the designated treatments on 4T1-implanted mice (n = 3 per group). **(B)** Representative histograms of nanoparticle internalization of

DCs ($CD11c^+$) and macrophages ($F4/80^+$) in TDLNs and **(C)** tumor collected 24 hours post treatments (n = 3 per group). **(D)** Percentages of $CD80^+$ and $CD86^+$ markers on $CD11c^+$ DCs and **(E)** $F4/80^+$ macrophages in blood (n = 3 per group). **(F)** Percentages of $CD80^+$ and $CD86^+$ markers on $CD11c^+$ DCs and **(G)** $F4/80^+$ macrophages in spleen (n = 3 per group). **(H)** Percentages of $CD80^+$ and $CD86^+$ markers on $CD11c^+$ DCs and **(I)** $F4/80^+$ macrophages in TDLNs (n = 3 per group). **(J)** Percentages of $CD80^+$ and $CD86^+$ markers on $CD11c^+$ DCs and **(K)** $F4/80^+$ macrophages in tumor (n = 3 per group). **(L)** Percentages of $F4/80^+MHC\ II^+$, **(M)** $F4/80^+CD206^+$, **(N)** $CD11b^+Ly6G^+$ and **(O)** $Ly6G^+MHC\ II^+$ cell populations in tumor (n = 3 per group). **(P)** Representative photographs of expression on $CD4^+$ subsets and **(Q)** $CD8^+$ and **(R)** $CD4^+$ subsets of $IFN\gamma^+$ secreting T lymphocytes in tumors collected at **(P)** 24 hours, **(Q)** and **(R)** 7 days post intravenous treatment with different nanoparticles (n = 3 per group). **(S)** splenic central memory T cells ($CD4^+CD44^+CD62L^+$) subset in spleen and **(T)** effector memory T cells ($CD4^+CD44^+CD62L^-$) subset in tumor collected from 4T1 tumor mice model (n = 3 per group) after second injections of various treatments on day 23. **(U)** Representative photographs of plasma cell populations ($CD138^+B220^-$) in spleen and **(V)** TDLNs collected post 23 days from 4T1 tumor-bearing mice (n = 3 per group) after two administrations of different treatments.
